# Discovery of Novel Biosynthetic Gene Cluster Diversity From a Soil Metagenomic Library

**DOI:** 10.3389/fmicb.2020.585398

**Published:** 2020-12-07

**Authors:** Alinne L. R. Santana-Pereira, Megan Sandoval-Powers, Scott Monsma, Jinglie Zhou, Scott R. Santos, David A. Mead, Mark R. Liles

**Affiliations:** ^1^Department of Biological Sciences, Auburn University, Auburn, AL, United States; ^2^Lucigen Corporation, Middleton, WI, United States; ^3^Varigen Biosciences Corporation, Madison, WI, United States

**Keywords:** metagenome, soil, biosynthetic ability, next-generating sequencing, biases

## Abstract

Soil microorganisms historically have been a rich resource for natural product discovery, yet the majority of these microbes remain uncultivated and their biosynthetic capacity is left underexplored. To identify the biosynthetic potential of soil microorganisms using a culture-independent approach, we constructed a large-insert metagenomic library in *Escherichia coli* from a topsoil sampled from the Cullars Rotation (Auburn, AL, United States), a long-term crop rotation experiment. Library clones were screened for biosynthetic gene clusters (BGCs) using either PCR or a NGS (next generation sequencing) multiplexed pooling strategy, coupled with bioinformatic analysis to identify contigs associated with each metagenomic clone. A total of 1,015 BGCs were detected from 19,200 clones, identifying 223 clones (1.2%) that carry a polyketide synthase (PKS) and/or a non-ribosomal peptide synthetase (NRPS) cluster, a dramatically improved hit rate compared to PCR screening that targeted type I polyketide ketosynthase (KS) domains. The NRPS and PKS clusters identified by NGS were distinct from known BGCs in the MIBiG database or those PKS clusters identified by PCR. Likewise, 16S rRNA gene sequences obtained by NGS of the library included many representatives that were not recovered by PCR, in concordance with the same bias observed in KS amplicon screening. This study provides novel resources for natural product discovery and circumvents amplification bias to allow annotation of a soil metagenomic library for a more complete picture of its functional and phylogenetic diversity.

## Importance

Soil microorganisms have been an important reservoir of antibiotic and other bioactive compounds, yet our knowledge of their biosynthetic gene clusters (BGCs) has been limited by culture- and PCR-biases. Direct cloning of soil metagenomic DNA can circumvent these limitations, yet identification of BGCs from metagenomic sequences can also be biased. The approach developed in this study used next-generation sequencing in a pooled format, enabling bioinformatic identification of gene content associated with individual metagenomic clones. This approach uncovered heretofore undiscovered BGC and phylogenetic diversity revealing that much of our current knowledge has been obscured by traditional culturing or targeted amplification strategies. We conclude that a more complete exploration of environmental metagenomes for their biosynthetic potential can be achieved using this approach. Its application can be extended to the detection of any genes of interest, thereby improving the power of functional metagenomic studies.

## Introduction

There is a tremendous degree of microbial diversity in soils (Torsvik and Ovreas, 2002), and soilborne microorganisms are key to biogeochemical and ecological processes. Interactions between microorganisms are, to a large extent, mediated by bioactive natural products that are encoded by BGCs (Reddy et al., 2012; Hill et al., 2014; Wang et al., 2014). Natural products from soil microorganisms have historically been a rich resource for antibiotics (Schatz et al., 1944; Ling et al., 2015; Hover et al., 2018); however, the vast majority of encoded BGCs have yet to be characterized since most soil microbes are recalcitrant to cultivation (Janssen, 2006; Howe et al., 2014; Soliman et al., 2017; Orellana et al., 2018). Culture-independent, amplification-based surveys have resulted in significant insights into microbial ecology and population dynamics (Schöler et al., 2017), while having their own inherent biases that obscure and distort our view of microbial diversity due to PCR amplification biases (Eloe-Fadrosh et al., 2016). Therefore, adoption of metagenomic approaches that avoid amplification biases, such as cloning of nucleic acids from natural environments (Rondon et al., 2000) or shotgun sequencing (Howe et al., 2014), have provided access to hitherto unknown reservoirs of microbial diversity and their encoded natural products (Daniel, 2004; Owen et al., 2013; Sharrar et al., 2020).

Shotgun metagenomic sequencing has allowed new insights into the functional and phylogenetic composition of complex microbial assemblages. However, assembly of short-read metagenomic sequences is especially challenging from phylogenetically diverse microbial assemblages present in as soils, and this had precluded the assembly of complete genomes and their encoded BGCs (Howe et al., 2014; Kang and Brady, 2014b). The advent of genome-resolved metagenomics through binning approaches has now enabled recovery of intact BGCs from shotgun sequenced metagenomic DNA (Crits-Christoph et al., 2018; Sharrar et al., 2020). Despite these developments in uncovering BGC diversity, direct sequencing does not lend itself to heterologous expression of BGCs in order to identify the encoded secondary metabolites. In order to access and express complete BGCs, bacterial artificial chromosome (BAC) vectors can be used to clone contiguous genomic fragments that can exceed 300 Kb, thereby increasing the probability of obtaining intact BGCs from metagenomic sourced DNA, while also enabling inducible copy number in *E. coli* and conjugal transfer to heterologous expression hosts (Wild et al., 2002; Aakvik et al., 2009; Liu et al., 2010; Kakirde et al., 2011; Liu et al., 2016; Nasrin et al., 2018). Functional screening of metagenomic clones typically results in very low hit rates for enzymatic activities (Healy et al., 1995; Thies et al., 2016; Lewin et al., 2017) and even lower rates for discovering bioactive metabolites (Tulp and Bohlin, 2005); thus, it is desirable to first identify clones containing the target gene or BGC of interest. Screening metagenomic libraries using degenerate oligonucleotides targeting conserved BGC domains by PCR or other molecular methods can be an effective strategy to identify BGCs of interest (Owen et al., 2013; Owen et al., 2015; Charlop-Powers et al., 2016); however, because these methods rely on previously described BGCs for primer design, we hypothesized that probe or PCR-based approaches for BGC discovery would be biased and identify only a subset of the BGCs present within a metagenomic library. This hypothesized bias in BGC discovery using a PCR-based approach is analogous to the incomplete phylogenetic diversity observed using “universal” 16S rRNA gene-specific primers as compared to the vast phylogenetic diversity observed for the candidate phyla radiation (CPR) that has been recovered only through non-amplification strategies (Hong et al., 2009).

Among the classes of BGCs of greatest interest for producing diverse secondary metabolites are PKS and NRPS clusters. These BGCs encode modular enzyme complexes that produce molecules of incredible chemical diversity that include metabolites with antibacterial, antifungal, anti-cancer, cholesterol-lowering and/or insecticidal bioactivities (Cragg and Newman, 2013). The modular arrangement of these mega-synthases and their large number of domains can result in long, contiguous clusters that can be challenging to reconstruct by direct sequencing or are too long to be captured intact by fosmid or cosmid metagenomic libraries (Kallifidas and Brady, 2012).

In this study we screened a large-insert soil metagenomic BAC library hosted in *E. coli* (Nasrin et al., 2018) using multiple sequence-based approaches, including PCR amplification of 16S rRNA genes and KS domains associated with PKS clusters, as well as using a novel next-generation sequencing (NGS) strategy to mine the entire library for BGCs. A multiplexed pooling strategy was used to allow bioinformatic identification of sequences associated with each of the 19,200 clones formatted in 384-well plates. The collection of metagenomic clones of interest, identified by PCR or by NGS, were then compared both from phylogenetic and biosynthetic perspectives, demonstrating the power of the NGS approach in providing a source of previously undescribed BGCs that may be heterologously expressed to produce secondary metabolites with potential therapeutic applications.

## Materials and Methods

### Bacterial Strains

The metagenomic library was cloned using the *E. coli* DH10B BAC-Optimized Cells (Lucigen Corporation, Middleton, WI), which are competent cells developed specifically for BAC library construction. These cells contain an arabinose-inducible *trfA* gene for BAC vector copy-induction (Wild and Szybalski, 2004).

### Soil DNA Isolation and Metagenomic Library Construction

Bulk soils were sampled from 10 to 30 cm below the soil surface, in a plot that had not received fertilizer for at least 100 years in the Cullars Rotation (Auburn, AL, United States) and was at the time planted with a soybean crop. The soil samples were transported to the laboratory where sub-samples were immediately frozen at −80°C, and the majority of the soil samples were maintained at 4°C until processed within one week for DNA isolation. A 10 g soil sample was processed for high molecular weight (HMW) DNA isolation and purification as previously described (Liles et al., 2008), and the metagenomic DNA was randomly sheared. The fragmented DNA was end-repaired, and *Bst*XI adaptors ligated on to the DNA, followed by gel-fractionation and ligation into the pSmartBAC-S vector as previously described (Nasrin et al., 2018). The vector:insert ligation was transformed by electroporation into BAC-optimized *E. coli* DH10B (Lucigen Corp, Middleton, WI, United States), and transformants were selected on LB agar (10 mg/mL of Tryptone, 10 mg/mL of NaCl, 5 mg/mL of Yeast Extract, 15 mg/mL of Agar in 1L of water) containing 12.5 μg/ml chloramphenicol. The soil metagenomic library consisted of 19,200 clones arrayed into 384 format (50 plates) with an average size of 113 Kb (Nasrin et al., 2018).

### Metagenomic Library Pooling and Sequencing

The library was divided into 5 sets of 10 plates: Set 1 (Plates 1–10); Set 2 (Plates 11–20); Set 3 (Plates 21–30); Set 4 (Plates 31–40) and Set 5 (41–50). For Set 5, the initial pooling strategy merged all 384 clones from each original library plate into a single plate pool (10 plate pools); row clones from the 10 Set 5 library plates into single row pools (16 row pools A-P); and column clones from the 10 Set 5 library plates into single column pools (24 column pools) (Supplementary Figure 1). For the remainder of the library (Sets 1 through 4), the 384-well plates from each Set were divided into 4 quadrants and replicated into 96-well plates, for a total of 40 × 96-well plates from each original Set. The resulting 96-well plates were pooled according to their Set of origin, resulting in 40 plate pools, 8 row pools A-H and 12 column pools (Supplementary Figure 1). For all pooling approaches, individual clones were grown in triplicate in 96-well plates using 1 ml LB containing 0.01% arabinose to amplify BAC copy number^21^. Pools were made by combining the liquid cultures as appropriate, pelleting the cells, and purifying BAC DNA, as previously described (Wu et al., 1999). Fragment libraries for sequencing on Illumina were constructed with 100 ng purified BAC DNA from each pool using the multichannel protocol of the NxSeq^®^ UltraLow DNA Library Preparation Kit (Lucigen, Middleton WI, United States). Unique indexes were used for each library pool within each batch of 10 library plates (Sets). Libraries were multiplexed and sequenced on Illumina HiSeq 2500 with v3 chemistry at 2 × 150 bp.

### Assembly *de novo* of Metagenomic Contigs

The raw HiSeq reads for each column, plate, or row pool were transferred to the Alabama Supercomputer (ASC) for processing. Reads were filtered for high quality reads (*Q* score > 30), trimmed, and clipped, and reads smaller than 30 bp discarded using the software Trimmomatic (Bolger et al., 2014). To remove host and vector DNA sequences, all processed reads were mapped against *E. coli* DH10B and the vector pSmartBAC-S sequences using BWA (Li and Durbin, 2009), and those un-mapped to the reference were then assembled using metaSPAdes implementation of SPAdes 3.9.0 software^74^. Reads corresponding to each respective sequencing pool were assembled individually, resulting in a set of contigs from each one of the 210 metagenomic library pools. In addition, pools from Set 3 of the Cullars metagenomic library were assembled using biosyntheticSPAdes available with SPAdes 3.14.1 (Meleshko et al., 2019) software, in order to compare the ability of each assembler in assembling BGCs from the metagenomic library. Since no significant differences were found in the total number of BGCs predicted, metaSPAdes assembly was used for downstream analyses.

### Library Deconvolution From Contig to Clone

All contigs generated from the metaSPAdes assembly were tentatively deconvoluted to a clone location using a custom bash script. Briefly, the deconvolution process consisted of renaming each individual contig to include their pool of origin and a unique number identifier. Contigs from the plate pools were compared to those in the column or row pools via BLASTn with 95% identity and a 10^–6^ e-value cut-off. The BLAST hits were extracted and annotated into three categories: (1) completely deconvoluted – plate contigs with hits in both column and row pools; (2) partially deconvoluted – plate contigs with hits in only one other dimension; or (3) singletons – contigs with no significant hits. After each contig was annotated, the location information in the contig name was used to generate coordinates (plate, column, and row) for the respective clone of origin.

### Statistical Analysis of Library Sequencing Data

A table containing contig name, length, coverage, pool dimension, deconvolution status, and set number for all contigs from the library were analyzed in R studio version 1.2.1335-1. Normality checks were performed using Q-Q plots while correlation between contig length and contig coverage was evaluated using Spearman’s test. Differences in contig length and coverage across sets and deconvolution status were evaluated using Kruskal-Wallis and Dunn tests. The analysis was conducted with and without outliers removed using the non-parametric Tukey’s method with a factor of 1.5xIQR, in order to determine the impact of outliers on some correlations.

### antiSMASH Prediction of BGCs From Library Contigs

A local version of antiSMASH 4.0 (Blin et al., 2017) with prodigal (meta) for gene prediction was used to predict BGCs from plate pools, which had the greatest coverage per pool. The program was run in the Bioconda environment on the ASC to afford high-throughput detection. Annotations for the library contigs containing PKS and/or NRPS clusters were conducted by importing the BiosynML antiSMASH 4.0 output of assembled library contigs and individually re-sequenced inserts into the Geneious software suite.

### Comparison of Conserved Domains From PKS and NRPS Clusters With Domains From Databases

KS domains and A domains from predicted PKS and NRPS clusters, respectively, were extracted and translated using the bacterial translation table. For comparison to known NRPS or PKS clusters, the MIBiG database (Epstein et al., 2018) (accessed 06/14/2019, version 1.4) was used to obtain a well-curated database of KS and A-domains. Prior to comparison, the KS and A domains database was clustered using MMseqs2 (Steinegger and Soding, 2017) to clusters of 75% amino acid similarity to decrease computational requirements for multiple alignment. Library sequences and MIBiG database sequences were aligned using MUSCLE (Edgar, 2004) and trimmed using trimAl (Capella-Gutierrez et al., 2009) automated algorithm to optimize signal x noise ratio. Following automated trimming additional sequences were dropped to ensure all alignments had the same set of library sequences. A maximum likelihood tree with 1000 iterations for bootstrap support was generated using RAxML 8.0 (Stamatakis, 2014). The annotations generated by antiSMASH 4.0 were combined with MIBiG GenBank files and analyzed using BiG-SCAPE (Navarro-Munoz et al., 2020) with clustering set to 0.3. The obtained networks were imported into Cytoscape (Smoot et al., 2011) for network visualization.

### Taxonomic Origin of PKS/NRPS Containing Library Contigs

For taxonomic origin inference, k-mer profiles and taxonomic binning of all contigs containing BGCs and all the validated insert sequences was performed using Kraken (Wood and Salzberg, 2014). The predicted taxonomic origin of validated insert sequences was further assessed by matching gene annotation information and k-mer profiles to achieve a consensus taxonomic classification.

### Re-sequencing Identified Clones, Annotation, and Taxonomic Origin

Selected clones identified as containing PKS and/or NRPS clusters were individually grown from the *E. coli* cryostock and the presence of the targeted BGC confirmed by insert-specific PCR (data not shown). The isolated BAC DNA was re-sequenced using a MiSeq sequencer (Illumina, San Diego, CA, United States). Originally, trimming and assembly was conducted with CLC Genomics Workbench 8.5 followed by manual inspection and reassembly, and antiSMASH 4.0 was used for prediction of BGCs from fully-assembled clone insert sequences. Inserts with antiSMASH annotation matching that of their associated contig were considered validated.

Additionally, processed reads were reassembled using biosynthetic-SPAdes available in the package SPAdes 3.14.1 and BGCs were predicted by antiSMASH 5.0 (Blin et al., 2019), in order to compare BGC assembly and detection from resequenced inserts with recently updated analysis tools. Annotations for the resequenced inserts were extracted from antiSMASH 5.0 output using the package Palantir (Meunier et al., 2020) available from metaCPAN. Specifically, PKS and NRPS domains were extracted from the clusters to facilitate BGC annotations. The use of biosyntheticSPAdes for assembly performed better for BGC annotations and was therefore used for downstream annotations. For in-depth annotation of three BGCs, ORFs and domains encoded within each ORF as predicted by antiSMASH 5.0 were visualized using the R package gggenes v0.4.0 (Wilkins, 2019).

### Screening Libraries for PKS Clusters via PCR

All 19,200 BAC clones were screened for the presence of KS domains using degenerate PCR primers 5LL and 4UU (Parsley et al., 2011; Supplemental Table 1). Each 25 μl PCR reaction contained 10 pmol of 5LL and 4UU, 12.5 μl CloneID 1X colony PCR master mix with Taq DNA polymerase (Lucigen Corp.), and 1 μ1 overnight growth of supernatant containing *E. coli* BAC DNA.

**TABLE 1:** Biosynthetic gene clusters identified from the soil metagenomic library by PCR and NGS approach.

Biosynthetic Gene Cluster Type	PCR Screening	NGS Screening	NGS Screening Deduplicated	Deconvoluted (%)
Type I PKS	17	75	39	73.3
Type I PKS-NRPS	27	100	33	86.0
Type II PKS	0	22	12	90.9
Type III PKS	1	114	71	84.2
Transatpks	1	6	3	100.0
Other KS	4	14	9	78.6
NRPS	0	509	160	68.4
Other	0	213	110	70.0
Terpene	0	371	201	80.9
Bacteriocin	0	224	127	77.2
Aryl polyene	0	70	36	67.1
Lassopeptide	0	61	32	83.6
Lantipeptide	0	33	23	84.8
Hserlactone	0	27	16	74.1
Resorcinol	0	17	14	88.2
Phosphonate	0	15	11	93.3
Indole	0	13	5	46.2
Ladderane	0	17	16	82.4
Acyl Amino acids	0	10	8	90.0
Butyrolactone	0	4	3	75.0
Microviridin	0	8	4	100.0
Siderophore	0	4	4	100.0
Cyanobactin	0	2	1	100.0
Thiopeptide	0	3	2	100.0
Linaridin	0	1	1	100.0
Phenazine	0	1	1	100.0
Ectoine	0	1	1	100.0
Hybrid Pathways*	0	147	72	100.0
Total	50	2082	1015	77.5

*“NGS Screening Deduplicated” refers to the corrected hit count after hits from contigs deconvoluted to the same clone of origin were deduplicated into a single hit. ^∗^Hybrid clusters included all clusters of mixed types except T1PKS-NRPS.*

Amplification was performed by conducting an initial denaturation at 94°C for 1 min, followed by 30 rounds of thermal cycling at denaturation at 94°C for 30 s, annealing temperature of 60°C for 30 s and extension at 72°C for 1 min, followed by 5 min of extension at 72°C. Reactions were considered positive if a ∼750 bp amplicon was visible upon agarose gel electrophoresis.

### Identification of 16S Ribotypes

16S rRNA gene candidates were mined from the Cullars soil metagenomic library by BLASTn search against the SILVA SSU nr database (Quast et al., 2012) and top hits with E-value lower than 1 × 10^–5^ and minimal initial alignment of 45 bp were computed. All hits smaller than 300 bp were discarded, and the remaining were annotated to the phyla level according to SILVA taxonomy at 75% identity.

### PCR Amplification and Sequencing of 16S rRNA Genes From the Cullars Metagenomic Library

All *E. coli* metagenomic library clones were grown separately in 96-well format overnight at 37°C and then the *E. coli* cultures were pooled into a single flask prior to DNA isolation using a Qiagen Large construct kit (Germantown, MD, United States). 16S rRNA genes were PCR amplified from the pooled Cullars metagenomic library DNA template with the “universal Bacteria-specific” primer set 27F and 1492R (Weisburg et al., 1991), using the following conditions: 94°C for 2 min, followed by 30 cycles of 94°C for 30 s, 55°C for 15 s and 72°C for 1 min, after which a final elongation step at 72°C for 5 min was performed. PCR products were visualized through gel electrophoresis, and the 16S rRNA gene amplicons were purified using the EZNA Cycle Pure kit (Omega Bio-tek, Norcross, GA, United States) and cloned in *E. coli* using the TOPO-TA cloning kit (Invitrogen, Carlsbad, CA, United States). Transformants were picked into a total number of eight 96-well plates and Sanger sequencing reactions were conducted with primer 27F (Lucigen Corp., Middleton, WI, United States). Sequences were trimmed using the CLC Genomics Workbench (CLC bio, Cambridge, MA, United States) and a BLASTn search conducted against the SILVA SSU nr database. All 16S rRNA gene sequences with a top affiliation to *E. coli* were presumed to have been derived from host genomic DNA that contaminated the BAC DNA isolation and were eliminated from the analysis.

### PCR Amplification and Sequencing of 16S rRNA Genes From Cullars Soil

The original Cullars Rotation soil sample used for library construction (stored at −80°C) was used for the 16S rRNA gene survey. Metagenomic DNA was isolated from 0.25 g of the soil sample using an EZNA Soil DNA kit (Omega Bio-tek, Norcross, GA, United States) and the gDNA was used as a template for bar-coded 16S rRNA gene sequencing targeting the V4 variable region with PCR primers 515F and 806R (Liu et al., 2007) and were used in a single-step 30 cycle PCR using the HotStarTaq Plus Master Mix Kit (Qiagen, United States) under the following conditions: 94°C for 3 min, followed by 28 cycles (5 cycle used on PCR products) of 94°C for 30 s, 53°C for 40 s and 72°C for 1 min, and final elongation step at 72°C for 5 min. Sequencing was performed at Molecular Research (Shallowater, TX, United States) on an Ion Torrent PGM following the manufacturer’s guidelines. Sequence data were processed by removing barcode and primer sequences, then ambiguous sequences, sequences with less than 150 bp or that had homopolymer runs exceeding 6 bp, were removed. The remaining sequences were denoised, OTUs generated and chimeras removed. Operational taxonomic units (OTUs) were defined by clustering at 97% similarity. Final OTUs were taxonomically classified using BLASTn against the SILVA SSU nr database.

## Results

A large-insert metagenomic library was constructed in the shuttle vector pSMART-BAC-S using high molecular weight DNA isolated from the Auburn University’s Cullars Rotation agricultural soil (Nasrin et al., 2018). A total of 19,200 independent clones were picked as isolated *E. coli* DH10B colonies into 50 plates in 384-well format. The insert size of 215 randomly selected BAC clones was 12 Kb to >200 Kb, with an average insert size of 113 Kb (Nasrin et al., 2018).

### PCR Amplification and Sequencing of KS Domains From the Library

Degenerate primer sets targeting the conserved beta-ketoacyl synthase (KS) domain were used to screen for PKS clusters. Each KS domain primer set (Supplementary Table 1) was tested for its ability to generate a PCR product using a pooled metagenomic library BAC DNA template, in which all 19,200 clones were pooled, and only the primer set 5LL/4UU was observed to give a PCR product (data not shown). The 5LL/4UU primer set was therefore used to amplify each of the 19,200 clones, in two rounds of screening to eliminate false positives, resulting in 925 clones selected, pooled, and sequenced. Contigs from the assembled pooled amplicons were evaluated by antiSMASH2.0, resulting in identification of 50 clones carrying PKS or PKS/NRPS hybrid gene clusters, for a final PCR-based screening hit rate of 0.26%.

### NGS of the Library Using a 3D Pooling Strategy

Two three-dimensional pooling strategies were devised in order to generate bar-coded libraries for NGS of the 19,200 clone library. In each strategy, every clone in the library was sequenced three times in bar-coded pools for each plate, column, and row, with each pool assembled individually for a total of 210 pools of contigs (Figure 1). By comparing the occurrence of each assembled contig in each dimension, a three-coordinate system was generated to associate each clone with a specific well. The two different pooling approaches, one using 384-well formatted plates and the other using 96-well formatted plates, allowed the evaluation of the effects of different numbers of clones per pool for BGC detection. For each pooling strategy, the library was screened by pooling of (a) ***each plate separately***; (b) all wells of ***each column across plates*** to create columns pools; and (c) all wells of ***each row across plates*** to create row pools (Supplementary Figure 1). A bar-coded library was constructed for each respective pool, and all libraries were sequenced together in a single Illumina HiSeq lane. The metagenomic library was divided into 5 sets that each contained 10 plates: Set 1 (Plates 1–10); Set 2 (Plates 11–20); Set 3 (Plates 21–30); Set 4 (Plates 31–40); and Set 5 (41–50). In the first pooling strategy we used a 384-well format for Set 5, while for Sets 1–4 we used a 96-well format in order to reduce the number of clones per each plate. This latter method significantly increased the coverage per plate pool, since each pool was composed of a smaller number of clones.

**FIGURE 1:**
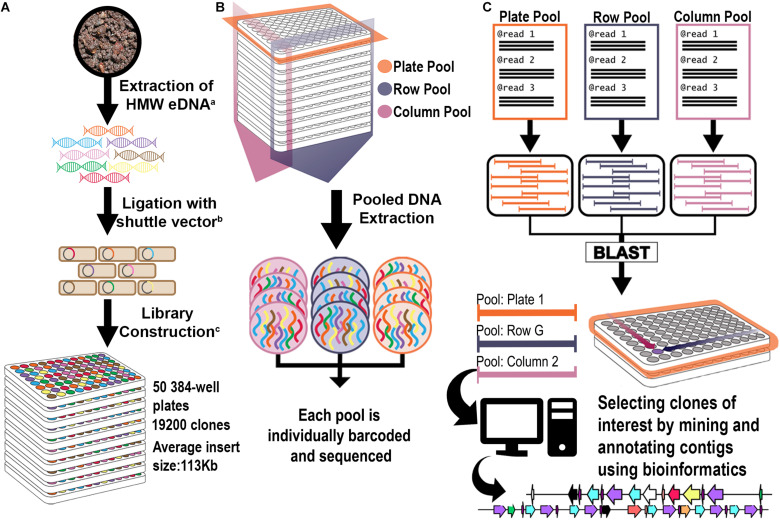
Library construction and 3D pooling strategies. **(A)** Library construction strategy. Superscript letters correspond to citations: a: (Liles et al., 2008); b: (Kakirde et al., 2011); c: (Nasrin et al., 2018). **(B)** 3D Library Pooling strategy: Plate, Column, and Row are barcoded before sequencing. **(C)** Contig to clone deconvolution and *in silico* mining strategy. Reads from each pool are assembled separately into pool contigs. Highly homologous contigs from each dimension (Plate, Column, and Row) form triplets. Since their pool of origin is known, they form a coordinate system that points to their clone of origin. Contigs were analyzed bioinformatically for rational identification of candidates for functional screening.

A significant number of reads (average of 33%) were removed that corresponded to host or vector sequences. Since the library represents a collection of different genomes, *de novo* assembly was primarily conducted using metaSPAdes. Additionally, a parallel assembly of 1/5th (Set 3) of the metagenomic library was done using biosynthetic-SPAdes (“Bio SPAdes”), a BGC assembler designed for BGC assembly from metagenomic/genomic sequences. The contigs resulting from these two assemblers run in parallel were compared in order to evaluate the adequacy of our assembly strategy. Contigs from each Set 3 assembly were analyzed by antiSMASH 5.0. BioSPAdes led to the prediction of 8 additional NRPS clusters compared to metaSPAdes; however, BLAST comparisons between BGCs carrying contigs from both assemblies revealed that in each case the additional NRPS clusters predicted from the bioSPAdes assembly were contained within a larger contig produced from the metaSPAdes assembly (Supplementary Figure 2). Since both assemblers performed similarly, but with metaSPAdes generating longer clusters in some instances, the metaSPAdes assembler was deemed most appropriate for library assembly. The contigs obtained from each library set were then deconvoluted to a specific BAC clone position in the 384-well plate by comparing the occurrence of each contig in each dimension (plate × column × row) using an automated BLAST analysis (Figure 1C). From the 3,157,354 contigs generated, 38% were successfully deconvoluted to a specific BAC clone location, corresponding to 14,976 clones (78% of the library).

The effect of sequencing coverage on assembly statistics was determined. As expected, higher sequencing coverage obtained with the second pooling approach using 96-well plate pools provided larger contigs. This strong positive correlation (ρ = 0.398, *P* < 2.2e-16) was even stronger when outliers were removed (ρ = 0.477, *P* < 2.2e-16). Scatter plots indicated that increasing clone sequencing coverage resulted in increased contig length until approximately 55X coverage was achieved (Supplementary Figure 3), while increasing sequencing coverage above 55X did not result in increased contig length. In fact, the majority of contigs with the greatest observed coverage had very small lengths, suggesting they correspond to repetitive or highly conserved sequence elements occurring in many microbial genomes.

We hypothesized that longer contigs would be easier to deconvolute to specific BAC clones. Both the Kruskal-Wallis test (analogous to an ANOVA) and the Dunn test showed that contig length was a significant (*P* < 2.2e-16) predictor of deconvolution success, with completely deconvoluted contigs having a much higher median and average length compared to singletons (Supplementary Table 2). Thus, the use of the second pooling strategy, which increased clone coverage, also generated larger contigs and in turn improved final BGC reconstruction from the metagenomic library.

### Mining the Library for BGCs

Contigs longer than 1 Kb were mined for BGCs using antiSMASH4.0, predicting the presence of 2,082 BGCs within the soil metagenomic library contigs. After deduplicating hits, referring to correcting the number of contigs belonging to the same clone when possible, there were a total of 1,015 BGCs predicted to be present in library clones, corresponding to 5.3% of all library clones (Table 1). Of these, 160 of the predicted BGCs corresponded to NRPS clusters, 134 were predicted as PKS clusters, among which were 39 Type I PKS, 12 Type II PKS, 71 Type III PKS, 3 *Trans*-PKS, and 9 Other KS; and 33 PKS-NRPS hybrid BGCs. From the Type I PKS and/or NRPS hits, 489 (71.5%) were successfully deconvoluted to 223 BAC clones, encompassing all but 9 PCR hits for PKS (the coordinates for which were already known, and these PCR-identified clones were also included within the NGS-discovered contigs). Screening the library for PKS-containing clones using NGS provided a greater number of clones (*n* = 158) compared to PCR (*n* = 50), which is to be expected given that any single PCR primer set would not be expected to amplify the broad diversity of PKS clusters identified by a homology-based search. Furthermore, NGS screening of the library allowed for parallel mining for different BGC types, leading to a much larger number of BGCs (*n* = 1,015) and a diversity of BGC classes (*n* = 27) that were recovered from the metagenomic library.

### Diversity of NRPS and PKS Clusters Identified From the Library

**FIGURE 2:**
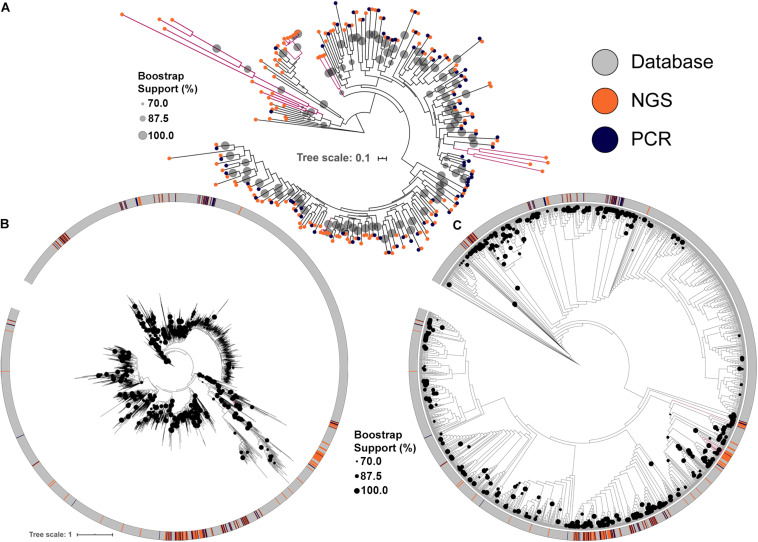
Maximum likelihood tree of KS domains recovered from the soil metagenomic library. Clades in magenta are formed uniquely by domains recovered by NGS. **(A)** KS domains recovered either by PCR or by NGS. **(B)** KS domains recovered from the soil metagenomic library and from the MIBiG database showing branch lengths. **(C)** KS domains recovered from the soil metagenomic library and from the MIBiG database showing topology only to facilitate visualization. Circles in the tree represent branch bootstrap support >70%.

The conserved KS domains from PKS clusters were used to build maximum likelihood (ML) dendrograms for direct comparisons between PKS clusters identified either by PCR, by NGS or present in the MIBiG database, a curated and comprehensive repository of fully annotated and characterized BGCs. NGS screening increased the number of BGCs identified and allowed the identification of more diverse PKS clusters (Figure 2A). Importantly, some KS domains identified by NGS formed clades with strong bootstrap support that cluster only with library KS sequences, and were not discovered by PCR. These clades also typically had longer branch lengths, indicating greater divergence in their KS domains among each other and compared to representatives of clades identified by PCR (Figure 2A).

Comparing the library KS domains to a curated database of known complete PKS clusters confirmed the trend of novelty among the BGCs identified by NGS (Figures 2B,C). Four of the KS domains identified by NGS are maintained as monophyletic clusters that are divergent from sequences present in the MIBiG database (Figures 2B,C). Interestingly, the deepest branching KS domains from the soil metagenomic library were solely identified by NGS screening. The library KSs also greatly expanded the diversity of some clades in the database, which correspond to BGCs that may produce novel secondary metabolites.

**FIGURE 3:**
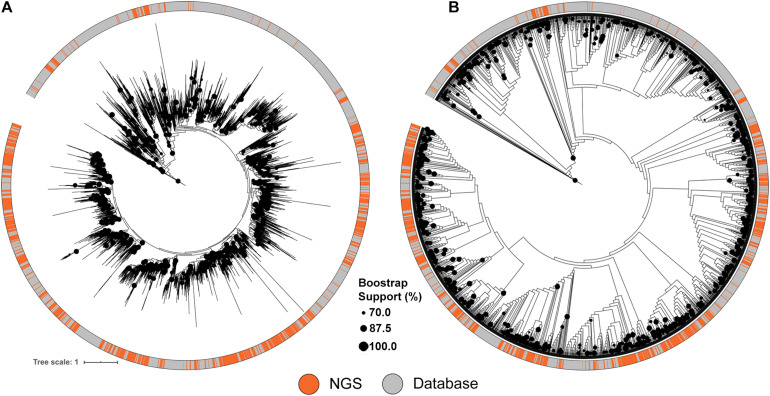
Maximum likelihood tree of A-domains recovered from NRPS clusters identified from the soil metagenomic library and the MIBiG database. **(A)** ML Tree showing branch lengths. **(B)** ML Tree ignoring branch lengths to facilitate topography visualization. Circles in the tree represent branch bootstrap support >70%.

A similar approach was used to compare the NRPS clusters to the MIBiG database, using conserved adenylation (A) domain sequences. The diversity of A domain sequences identified from the metagenomic library were affiliated with almost every database clade of the tree (Figure 3). Despite the comprehensiveness of the MIBiG database, monophyletic clades formed only by library-derived A domains further expand the diversity of described NRPS clusters.

**FIGURE 4:**
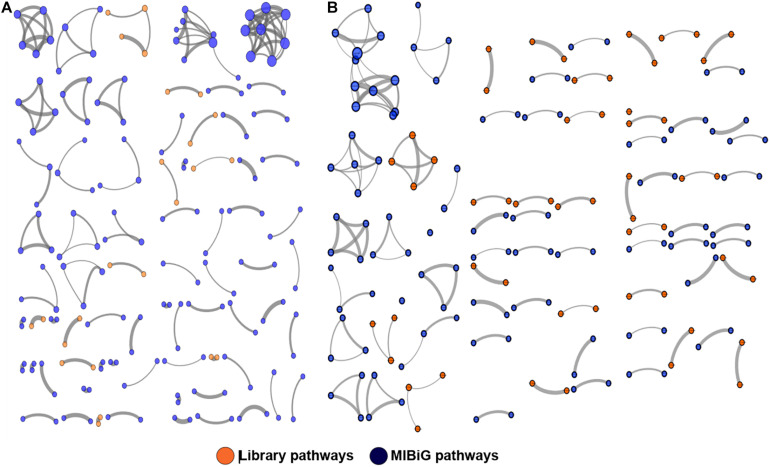
Clustering of PKS **(A)** and NRPS **(B)** pathways against the MIBiG database using BiG-SCAPE. Library pathways are shown in orange and database pathways in blue. Circle size is proportional to the number of connections, and strike width is proportional to the similarity between pathways.

To better visualize the relationships between library PKS and NRPS clusters versus those contained in the MIBiG database, the library-derived BGCs were clustered using the program BiG-SCAPE and visualized in Cytoscape. The library BGCs were shown to affiliate with diverse Pfam families and did not form links with MIBiG database entries, further highlighting their uniqueness relative to characterized BGCs (Figure 4).

The taxonomic origin of contigs containing PKS and/or NRPS gene clusters were inferred by comparing K-mer profiles using Kraken (Supplementary Figure 4), since only one clone (P35L08) also carried a 16S rRNA gene, impeding the general use of this gene for clone taxonomic classification. In the case of clone P35L08, it was predicted by Kraken to be derived from bacteria in the genus *Pseudolabrys* within the *Rhizobiales* order of α-Proteobacteria, which aligns with the prediction of the 16S rRNA gene annotation. This result supports the use of k-mer profiling of BGC-containing clones to infer their taxonomic origin. Further taxonomic resolution may be possible for some metagenomic clones depending upon the availability of phylogenetically informative gene sequences.

The largest taxonomic category for BGC origin was indicated as “unclassified,” with the phyla Proteobacteria and Actinobacteria being the most frequent among the 11 phyla identified, followed by the phyla Bacteroidetes and Firmicutes (Supplementary Figure 4). Interestingly, members of the phylum Actinobacteria were the most commonly predicted source of NRPS clusters, whereas Gamma-Proteobacteria were the most common predicted source of PKS clusters. Bacteroidetes and Firmicutes were also frequently predicted to be sources of NRPSs.

### Recovery of PKS and NRPS Clusters From the Metagenomic Library

From the deconvoluted clones predicted to contain PKS or NRPS clusters, BAC inserts of 221 clones were resequenced and annotated with antiSMASH 4.0 to validate the predictions from the metagenomic library contigs. Originally, inserts were assembled using CLC Genomics Workbench and BGCs were predicted using antiSMASH 4.0; however, in order to compare these results using the most recently available tools for BGC discovery, the same insert sequences were reassembled using bioSPAdes and these BGCs were then annotated using antiSMASH 5.0. The latter approach generated BGCs with more domains than the previous assembly in many cases, and therefore were preferred (Supplementary Figure 5). Validation was done by comparing antiSMASH 5.0 predictions for each clone and its associated contigs, and by comparing sequence similarity using BLAST. The BGCs from validated clones were then manually inspected and deemed viable if they contained at least 3 complete adjacent modules (for Type I PKS and NRPS). A total of 195 clones from the 221 clones resequenced were validated, with 117 of the inserts carrying viable PKS or NRPS BGCs while the rest were predicted by antiSMASH5.0 to contain truncated modules (Supplementary Table 3 and Supplementary Figure 6).

**FIGURE 5:**
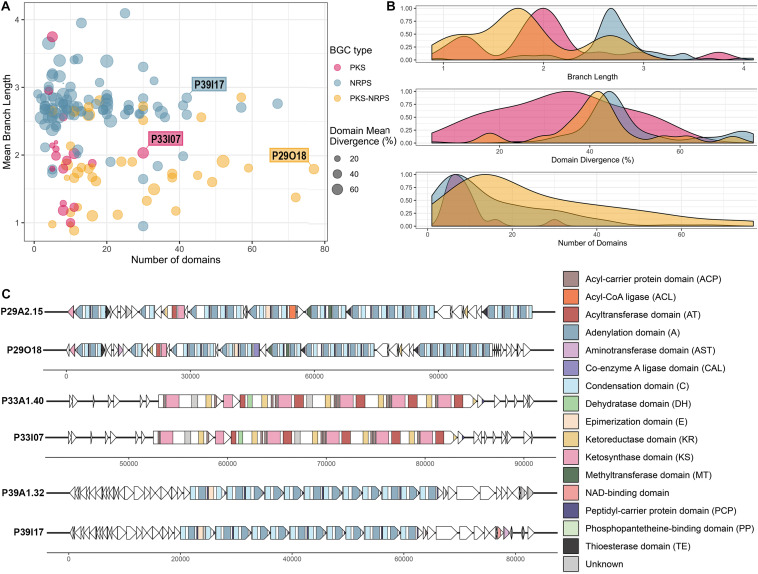
Analysis of the clones containing viable PKS and NRPS pathways reconstructed and validated from the Cullars metagenomic library. **(A)** Viable PKS and/or NRPS clusters recovered from the library compared by size (number of domains) and divergence from known BGCs (mean branch length and domain mean divergence). Each plot point represents a clone containing a viable BGC. Mean branch length for each clone is calculated as the average branch length of all the clones’ KS and/or A domains, as extracted from the ML trees against the MIBiG database. Mean domain divergence for each clone is calculated as the average domain divergence of all clones’ KS and/or A domains. Domain divergence is the complementary percentage to the% identity of the domain to the NCBI nr database (if a domain has% identity of 60, then the domain divergence is 40%). **(B)** Density distribution of branch length, domain divergence and number of domains across the viable PKS and NRPS BGCs. **(C)** Annotation of three interesting representative BGCs, representing the longest BGC from each type (PKS, NRPS, PKS-NRPS) to be recovered successfully *in silico* from the metagenomic library NGS contigs and validated upon clone resequencing. Annotation from the library contig was compared to the annotation from their corresponding validated insert sequence. ORFs are depicted by arrows and the PKS and/or NRPS domains within each ORF are represented by colored stripes.

Visualization of library BGCs in terms of their divergence from BGCs in the MIBiG database and their size can aid the process of selecting clones of interest that encode viable PKS/NRPS clusters (Figure 5A). The majority of the viable BGCs recovered had 10–20 domains with larger clusters being less frequent (Figure 5B). Hence, the importance of increased insert size, which allowed for the recovery of very large clusters (i.e. the largest having 77 predicted PKS-NRPS domains) was demonstrated despite their relative sparsity in the metagenomic library. The A domains and KS domains from the library BGCs were compared to the GenBank nr/nt database by BLASTp and the majority showed moderate similarity (mean of 54.7% identity, ranging from 23.8 to 95.0% identity) to other A and KS domains in the nr/nt database (Figure 5B and Supplementary Table 3). This moderate similarity can also be observed in terms of the branch lengths of the conserved KS and A domains carried by each viable BGC when compared to the MIBiG database. Most of the KS domains had a branch length of around 2, whereas with the A domains a longer branch length around 3 was more frequently observed (Figure 5B).

The A domain and KS domain dendrograms allowed for selection of three interesting representatives for more detailed annotation, including PKS-containing clone P33I07, NRPS- containing clone P39I17, and PKS-NRPS-containing clone P29O18. These three clones were the largest recovered BGCs of their type to be identified. The PKS-NRPS hybrid representative, clone P29O18, harbored the longest BGC recovered (>100 Kb) and the synteny between the contig and the insert clusters were highly similar (Figure 5C). The BGC consisted of 22 complete modules and 4 incomplete modules. Most of the modules were assigned as NRPS machinery, however, one complete PKS module was included. Several methyltransferase (MT) domains were predicted throughout the BGC which could have an important role in modification of the peptide intermediate. Additionally, the presence of thioesterase (TE) domains in the BGC may drive the release of the putative product.

The largest PKS recovered was a 30 Kb Type I PKS BGC from clone P33I07 which, if active, is predicted to synthesize a linear polyketide. Annotation of this gene cluster found that it contained 4 complete modules consisting of the minimum three catalytic domains: an acyltransferase (AT), a KS domain, and an acyl carrier protein (ACP). Each module carried the characteristic reductive ketoreductase (KR) domains which often double as epimerization domains and can alter the linear structure predicted by antiSMASH. In addition, one module carries a dehydrogenase (DH) domain to further tailor the nascent polyketide.

Clone P39I17 was selected as an NRPS representative for annotation based on the completeness of the BGC and >40% domain divergence. This BGC spanned 40 Kb with 12 complete modules consisting of the three core domains-a condensation (C) domain, an A domain, and a thiolation/peptidyl-carrier protein (PCP) domain. The starter module also carries an additional enzymatic, epimerization (E) domain capable of converting amino acids between the L- and D- isomers (Felnagle et al., 2008). One incomplete module lacking an A domain was predicted by antiSMASH 5.0 and no tailoring domains were evident on the BGC. Core structures predicted from antiSMASH 5.0 suggests that a linear NRP could be produced with low similarity to the antitumor peptide hemiasterlin B (Anderson et al., 1997).

### Phylogenetic Diversity of Cullars Soil and the Cullars Soil Metagenomic Library

**FIGURE 6:**
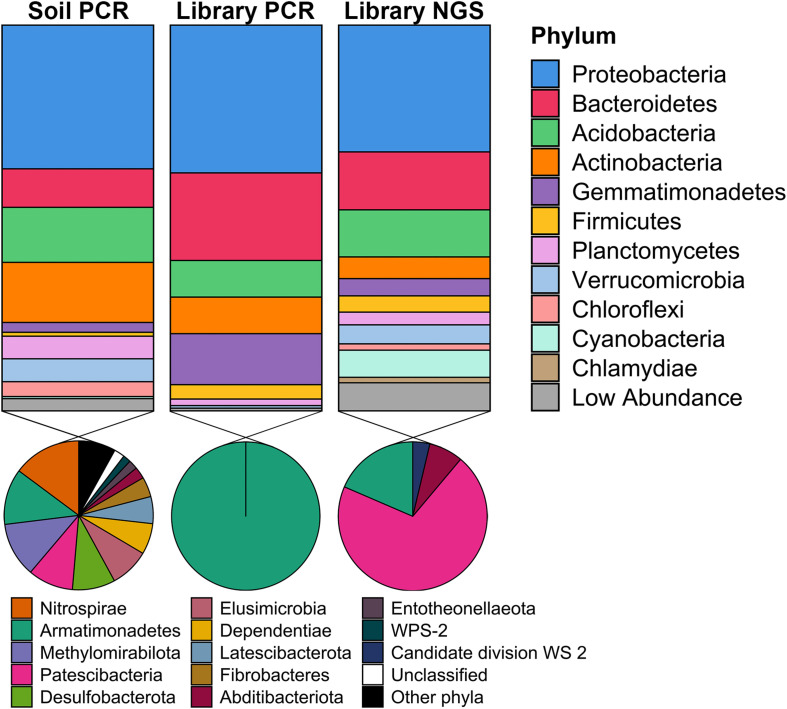
Taxonomic diversity of the Cullars metagenomic library and of the original soil sample used for library construction. Soil PCR: 16S rRNA gene PCR amplification of DNA isolated from the original Cullars soil sample used to construct the library; Library PCR: 16S rRNA gene PCR amplification of the DNA template from all metagenomic library clones pooled together; Library NGS: *In sillico* mining for 16S rRNA genes from the metagenomic library NGS contigs. The phyla contained in the section “Other phyla” are: RCP2-54, SAR324, Spirochaetes, Sumerlaeota, WPS-2; all with relative frequencies below 0.05%.

To determine the phylogenetic diversity captured in the metagenomic library, 16S rRNA genes were obtained from three sources: (1) PCR amplification of DNA isolated from the original Cullars soil sample used to construct the library (Original Cullars soil survey); (2) PCR amplification of the pooled DNA template from all metagenomic library clones (Cullars metagenomic library survey); and (3) Screening for 16S rRNA genes from the metagenomic library NGS contigs (Cullars metagenomic library NGS). The Original Cullars soil survey revealed a microbiota composition dominated by Proteobacteria (32.4%), Actinobacteria (15.6%), Acidobacteria (14.3%) and Bacteroidetes (10.0%), with a significant presence of Planctomycetes, Verrucomicrobia, Mixococcus, Chloroflexi and, at lower frequencies, Gemmatimonadetes (2.5%), Firmicutes (1.0%) and Cyanobacteria (0.5%) (Figure 6). In addition, 23 low abundance phyla were identified in the original soil sample including most notably Nitrospirae, Armatimonadetes, Methylomirabilota and Patescibacteria, in addition to several other understudied candidate taxa (Lynch and Neufeld, 2015; Figure 6).

For the Cullars metagenomic library survey, 295 16S rRNA gene amplicons were annotated after excluding *E. coli* host amplicons. The library 16S rRNA gene amplicon sequences were affiliated with 10 bacterial phyla, with Acidobacteria, Actinobacteria, Bacteroidetes, Gemmatimonadetes, and Proteobacteria accounting for 97.0% of the total observed ribotypes. Chloroflexi, Firmicutes, Planctomycetes, Armatimonadetes, and Verrucomicrobia constituted the remaining ribotypes (Figure 6). It should be noted that, while the phyla representation was generally similar for the PCR amplified 16S rRNA gene sequences obtained from the original soil, or the metagenomic library, that two different primer sets were used (due to the need for short amplicons for NGS in the case of the original soil), and that these two 16S amplicon data sets are not directly comparable.

16S rRNA sequences recovered from NGS of the metagenomic library identified 457 ribotypes affiliated with 20 different phyla. There was a predominance of Proteobacteria (32.9%), Bacteroidetes (22.7%), Acidobacteria and Actinobacteria (9.5%). NGS of the library was also more sensitive in detecting members of the PVC superphylum (Planctomycetes, Verrucomicrobia, and Chlamydiae), Cyanobacteria and Chloroflexi, as well as revealing that genomic inserts from rare soil taxa were captured, albeit in low abundance (Figure 6). Due to the differences in numbers of 16S rRNA genes sequenced from the library PCR amplicons vs. the library NGS, it is not surprising that a greater number of phyla were observed; however, these ribotypes identified sole from library NGS are affiliated with six classes of Patescibacteria, in addition to Armatimonadetes, Abditibacteria and Candidate division WS 2, all of which were also not identified from the original soil 16S survey and are typically missed by PCR amplicon surveys and only observed via direct metagenomic sequencing (Brown et al., 2015). Hence, just as we had observed based on BGC diversity, using NGS provided a more exhaustive picture of the phylogenetic diversity contained within the Cullars soil metagenomic library.

When compared to the original soil sample, the metagenomic library successfully captured representatives of the most common phyla in roughly similar proportions, with a noticeable reduction in the relative abundance of Actinobacteria when compared to the original soil. Recovery of rare taxa is a challenge when building metagenomic libraries, however the Cullars metagenomic library was able to capture some representatives of lower abundance phyla present in the original soil sample (Figure 6).

## Discussion

There is no better crucible for the generation of novel natural products than microbial competition in natural environments, especially among soil microbiota from which many and diverse natural products have been derived (Wohlleben et al., 2016). *In situ* culturing techniques have been successful in cultivating previously uncultured microorganisms that can produce bioactive metabolites (Ling et al., 2015). Nevertheless, the vast majority of environmental microbiota can only be exploited for their natural products via culture-independent approaches (Milshteyn et al., 2014). Indeed, functional screening of metagenomic clone libraries has led to the discovery of structurally and functionally diverse antimicrobial compounds (Kallifidas et al., 2012; Kang and Brady, 2013, 2014a; Hover et al., 2018; Nasrin et al., 2018), bioactive compounds (Fisch et al., 2009; Zimmermann et al., 2009; Chang and Brady, 2013; Owen et al., 2015), novel enzymes (Wang et al., 2010; Cheng et al., 2017; Kusnezowa and Leichert, 2017; Lewin et al., 2017; Nasseri et al., 2018), and novel promoters (Westmann et al., 2018).

Unfortunately, many technical hurdles such as inadequate metagenomic clone insert sizes, biased detection of BGCs, and poor heterologous expression render functional screening suboptimal, laborious and inefficient (Palazzotto and Weber, 2018). Therefore, rational selection of clones that are known to encode BGCs of interest for subsequent targeted heterologous expression could greatly improve the efficiency of identifying secondary metabolites expressed from metagenomic BGCs. Targeted screening of metagenomic libraries based on arrayed PCR or other amplification methods has been developed to enrich clones containing genes of interest, which can then be heterologously expressed and screened for bioactivity (Owen et al., 2013). Other approaches relying on functional complementation can further isolate clones of interest (Charlop-Powers et al., 2013; Bitok et al., 2017). However, these ingenious approaches do not provide insight into the BGCs or other functional genes carried by the metagenomic inserts in advance of their expression, so that selection of an ideal heterologous host for a specific BGC of interest is not possible.

This study demonstrates a bioinformatic pipeline for complete metagenomic library sequencing and mining for BGC-containing clones, providing full annotation of inserts prior to targeted heterologous expression. Attempts to leverage NGS capabilities to sequence collections of metagenomic clones have so far relied on a hybrid approach, for example using “tags” for each clone that are created via amplification and Sanger sequencing of individual clones, then pooling the clones and performing sequencing using NGS (Dzunkova et al., 2012; Calderon et al., 2019). In contrast, this study presents a strategy capable of directly sequencing large numbers of clones, while maintaining insert positional information within the library, a critical step in targeting specific cloned BGCs for conjugal transfer to a compatible heterologous host and functional screening for bioactive secondary metabolites. Thus, this pipeline expands the capabilities of previous targeted screening strategies by circumventing PCR bias and individual tagging, while allowing the use of bioinformatics to mine for diverse genomic features of interest. Given the known biases associated with PCR, it is plausible to assume that this approach would hasten the discovery of other classes of BGCs as well. Not only does NGS-based screening generate more hits, but this strategy results in discovery of highly diverse BGCs, which was evident for the PKS and NRPS clusters that are very different from those currently characterized. Hence, it is evident that our current exploitation of bioactive natural products from metagenomic libraries have been limited by earlier methods, and environmental BGCs remain a largely understudied and untapped resource for natural product discovery.

Furthermore, BGC annotation can be extended to prediction of metabolite structure, prior to heterologous expression. Indeed, we were able to annotate and deconvolute complete PKS and NRPS clusters, the longest of which spanned over 77 domains, and predict the linear structure of their encoded metabolites. While the metabolites expressed from these metagenome-derived BGCs in a heterologous host may be significantly different from the metabolites expressed in the original host, and the heterologous host may significantly affect BGC expression and metabolite chemistry (Gillespie et al., 2002), this approach does allow targeted, rational selection of BGC and expression host combinations. This approach can also improve the ability to detect and express other relevant classes of gene products, such as biotechnologically relevant enzymes. While this study focused on characterizing NRPS and/or PKS clusters, future research will explore the diversity of enzymes and other natural products encoded within the soil metagenomic library.

Most of the library PKS and NRPS clusters were not affiliated with any particular phylum, stressing the biotechnological potential of as-yet-uncultured bacteria such as Patescibacteria or Acidobacteria taxa, as potentially novel sources of BGCs to be further explored (Parsley et al., 2011; Crits-Christoph et al., 2018; Sharrar et al., 2020). Among those clusters that were classified, we observed a predominance of well-studied phyla, such as Proteobacteria, Actinobacteria, Bacteroidetes, and Firmicutes, whereas abundant yet less studied phyla, such as Acidobacteria, were underrepresented. The paucity of BGCs previously obtained from Acidobacteria and other abundant but poorly characterized soil microbiota is expected to have biased the prediction of BGC origin. In support of that hypothesis, manual inspection of the complete insert sequence for BGC-containing clones that have been shown to express antibacterial metabolites in a *Streptomyces coelicolor* host have suggested an Acidobacteria origin (data not shown). Nonetheless, there are notable shifts in relative abundance between phyla within the metagenomic library when compared to the original soil, which suggests there were biases associated with library construction (Lam and Charles, 2015). Improvements in DNA extraction and cloning techniques may increase the representativeness of microbial genomes accessed from a metagenomic library, widening the scope of BGCs available for functional screening. In spite of such biases, the Cullars soil metagenomic library was found to contain cloned genome fragments from diverse bacteria, including underrepresented taxa such as the Patescibacteria.

The Cullars metagenomic library survey of 16S rRNA genes using PCR amplification failed to recover many phyla that were identified by library NGS. The 16S survey of the metagenomic library therefore had the same biases encountered with KS amplicon screening, which were likewise overcome using a NGS approach. While efforts to revise universal primers have been proven useful (Frank et al., 2008; Caporaso et al., 2011; Klindworth et al., 2013), the inherent biases associated with PCR amplification will continue to plague efforts to catalog BGC and phylogenetic diversity. Akin to the unveiling of microbial diversity such as CPR and the Asgard superphylum of Archaea by shotgun metagenomic sequencing (Zaremba-Niedzwiedzka et al., 2017), the application of NGS to a soil metagenomic library has exposed an unprecedented diversity of BGCs from soil microorganisms that have been previously overlooked because of culture- and PCR-biases. The application of this NGS strategy to other metagenomic libraries would therefore be predicted to reveal heretofore undiscovered functional and phylogenetic diversity that have been captured in these genomic resources.

This pipeline provides a powerful way to annotate metagenomic libraries for rational exploration and screening of genomic features of interest. In the context of BGCs, we demonstrated that library sequencing can significantly improve hit rate, BGC diversity, and elucidation of pathways that may be then targeted for heterologous expression and production of bioactive compounds. The ability to fully leverage NGS and bioinformatic tools will enable a more complete assessment of the functional and phylogenetic diversity among metagenomic libraries and permit their full exploitation for biotechnological research.

## Data Availability Statement

The datasets presented in this study can be found in online repositories. The names of the repository/repositories and accession number(s) can be found below: NCBI BioProject, accession no: PRJNA669376.

## Author Contributions

DM and ML conceived of the research and secured funding for this project. AS-P, MS-P, SM, and JZ conducted the research experiments and analyzed the results. AS-P, JZ, and SS conducted the bioinformatics analyses. AS-P was primarily responsible for writing the first draft and all authors edited and approved the manuscript for submission. All authors contributed to the article and approved the submitted version.

## Conflict of Interest

ML and DM are the cofounders of the Varigen Biosciences Corporation. A licensing agreement between Auburn University and the Varigen Biosciences Corporation has been established for commercial development of the Cullars soil metagenomic library described in this manuscript. The remaining authors declare that the research was conducted in the absence of any commercial or financial relationships that could be construed as a potential conflict of interest.

## Supplementary Material

Supplementary Figure 1 Pooling strategies used in sequencing the Cullars metagenomic library. **(A)** The Cullars metagenomic library composed of 50 384-well plates were divided into 5 sets of 10 plates that were pooled for Plate × Row × Column individually, for a total of 210 pools. **(B)** Initial pooling strategy used in Set 5. All clones in each plate were combined to generate a plate pool, all clones from each row across the 10 plate set were combined to generate a row pool, and finally all clones from each column across the 10 plate set were combined to generate a column pool. Set 5 generated 50 pools that were individually barcoded and sequenced. **(C)** Second pooling strategy applied to Set 1, 2, 3 and 4 individually. For each Set, each 384-well plate was arrayed into quadrants composed of 96 clones that were replicated into 96-well plates. Thus, the initial 10 384-well plates were divided into 4 subsets of 10 96-well plates. All subsets are combined to generate the pools. All clones in each 96-well plate were combined to generate a plate pool, all clones from each row across the 96-well plate subsets were combined to generate a row pool, and finally all clones from each column across the 96-well plate subsets were combined to generate a column pool. Each Set from 1 through 4 generated 60 pools that were individually barcoded and sequenced.

Supplementary Figure 2 Comparison between metaSPAdes and biosynthetic-SPAdes (BioSPAdes) assemblies of 1/5th (Set 3) of the metagenomic library. **(A)** Number and type of detected BGCs in each assembly. Biosynthetic-SPAdes identified 8 extra NRPS hits. **(B)** BGC-carrying contig correspondence between assemblies as established by BLAST. Each biosynthetic-SPAdes contig matched only a single metaSPAdes contig identified by numbers from 1 to 152. When multiple biosynthetic-SPAdes contigs match the same metaSPAdes contig, a plateau is observed in the line graph. **(C)** Manual inspection of biosynthetic-SPAdes contigs matching the same metaSPAdes contig by BLAST, shows that contiguous sequences containing BGCs were fragmented by biosynthetic-SPAdes resulting in the increased hit count.

Supplementary Figure 3 Plots of contig Length x Coverage, both indicated as a log transform. No increase in contig length was observed past 55× coverage (Tcoverage = 4), with a very strong influence in final contig length up to 25X. **(A)** Length × Coverage separated by SET (groups of 10 Library plates). **(B)** Length × Coverage separated by deconvolution status. Higher amount of long contigs amongst the completely deconvoluted set was observed.

Supplementary Figure 4 Taxonomic classification of the contigs carrying PKS and/or NRPS pathways by kmer frequencies.

Supplementary Figure 5 Increase in the domain count of PKS and/or NRPS clusters recovered from the resequenced clones by assembly with either SPAdes or biosynthetic-SPAdes (bioSPAdes). Both assemblers showed cases where more domains were predicted within individual BGCs, however bioSPAdes performed better overall with respect to the overall total number of additional domains predicted.

Supplementary Figure 6 AntiSMASH 4.0 annotations for each contig and clones predicted to carry a PKS and/or NRPS pathway. **(A)** Contigs predicted to carry NRPS pathways; **(B)** Contigs predicted to carry PKS pathways; **(C)** Resequenced clones.

Supplementary Table 1 List of oligonucleotides sequences used in this study.

Supplementary Table 2 Basic statistics of contig lengths according to deconvolution status.

Supplementary Table 3 PKS and/or NRPS contigs and resequenced clones information. “BL mean” refers to the mean Branch Length of all KS and/or A domains from the BGC; “Domain Ident” is the mean percent identity for the top BLASTp hit for all A domain and/or KS domains identified from re-sequenced metagenomic clones compared to the NCBI nr/nt database.“Dom. Div” refers to domain divergence calculated as the opposite of Domain %ID (1-Domain %ID).
