# Supplementary figures and images for: Discovery of Novel Biosynthetic Gene Cluster Diversity From a Soil Metagenomic Library
Source: Front Microbiol. 2020 Dec 7;11:585398. doi: 10.3389/fmicb.2020.585398 (PMC7750434; doi:10.3389/fmicb.2020.585398)

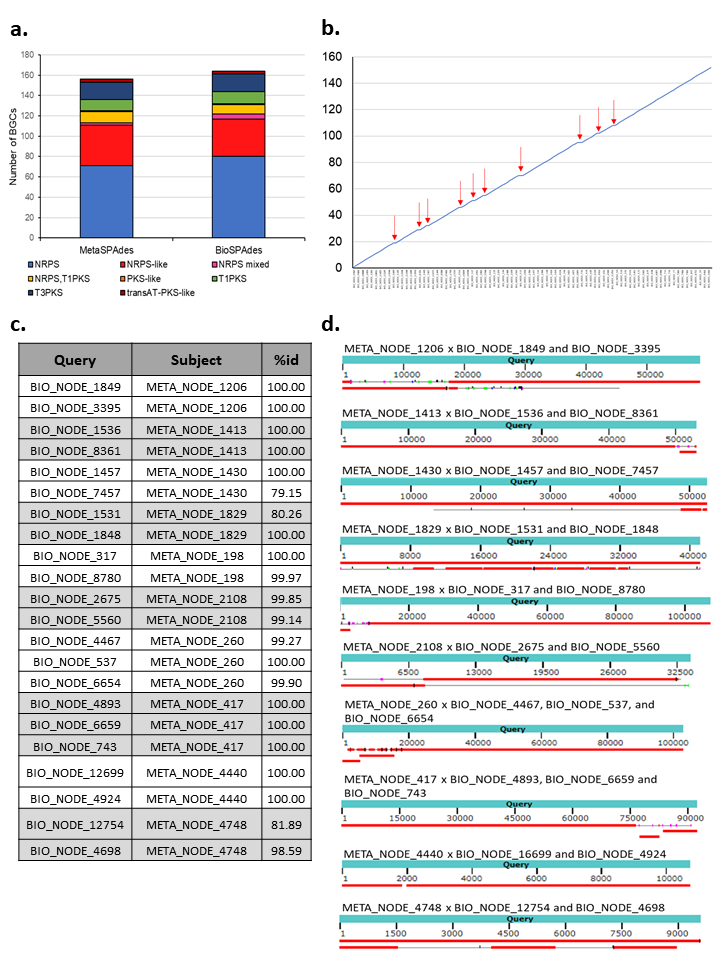

Supplement: Supplementary Figure 2 — Comparison between metaSPAdes and biosynthetic-SPAdes (BioSPAdes) assemblies of 1/5th (Set 3) of the metagenomic library. (A) Number and type of detected BGCs in each assembly. Biosynthetic-SPAdes identified 8 extra NRPS hits. (B) BGC-carrying contig correspondence between assemblies as established by BLAST. Each biosynthetic-SPAdes contig matched only a single metaSPAdes contig identified by numbers from 1 to 152. When multiple biosynthetic-SPAdes contigs match the same metaSPAdes contig, a plateau is observed in the line graph. (C) Manual inspection of biosynthetic-SPAdes contigs matching the same metaSPAdes contig by BLAST, shows that contiguous sequences containing BGCs were fragmented by biosynthetic-SPAdes resulting in the increased hit count. [file Image_2.TIF]

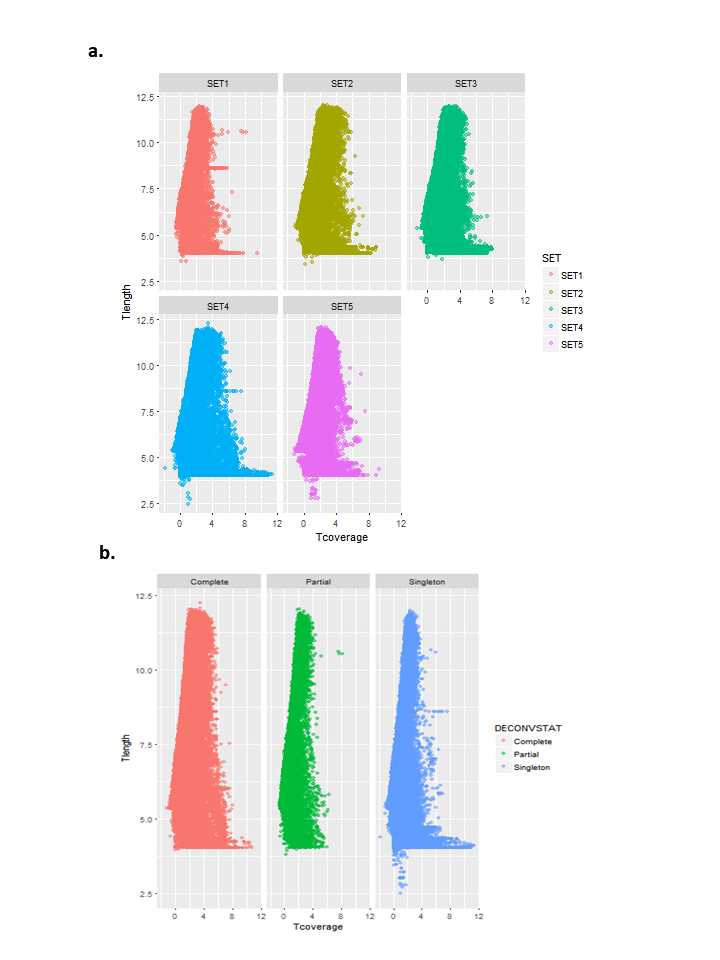

Supplement: Supplementary Figure 3 — Plots of contig Length x Coverage, both indicated as a log transform. No increase in contig length was observed past 55× coverage (Tcoverage = 4), with a very strong influence in final contig length up to 25X. (A) Length × Coverage separated by SET (groups of 10 Library plates). (B) Length × Coverage separated by deconvolution status. Higher amount of long contigs amongst the completely deconvoluted set was observed. [file Image_3.TIF]

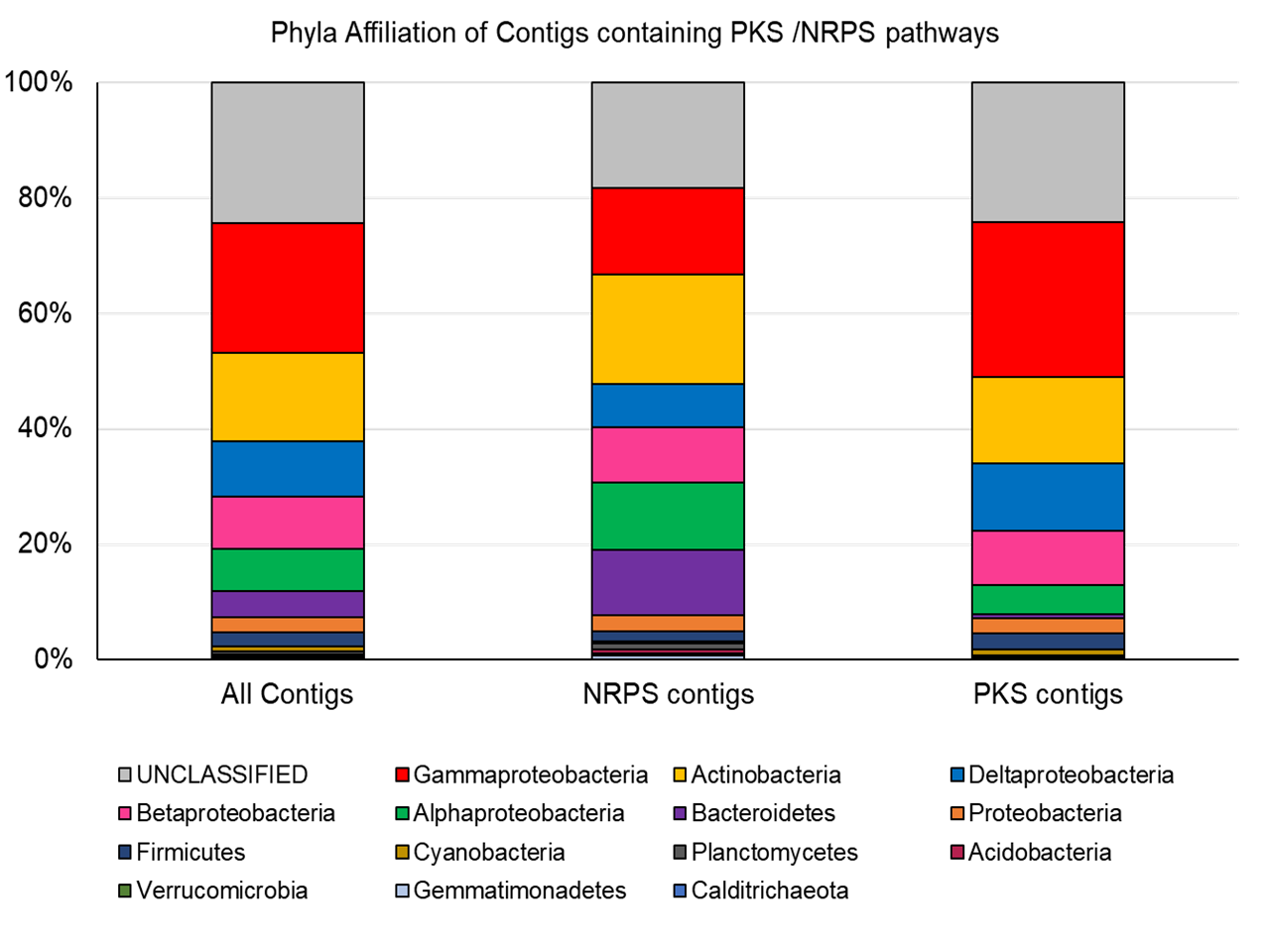

Supplement: Supplementary Figure 4 — Taxonomic classification of the contigs carrying PKS and/or NRPS pathways by kmer frequencies. [file Image_4.TIF]

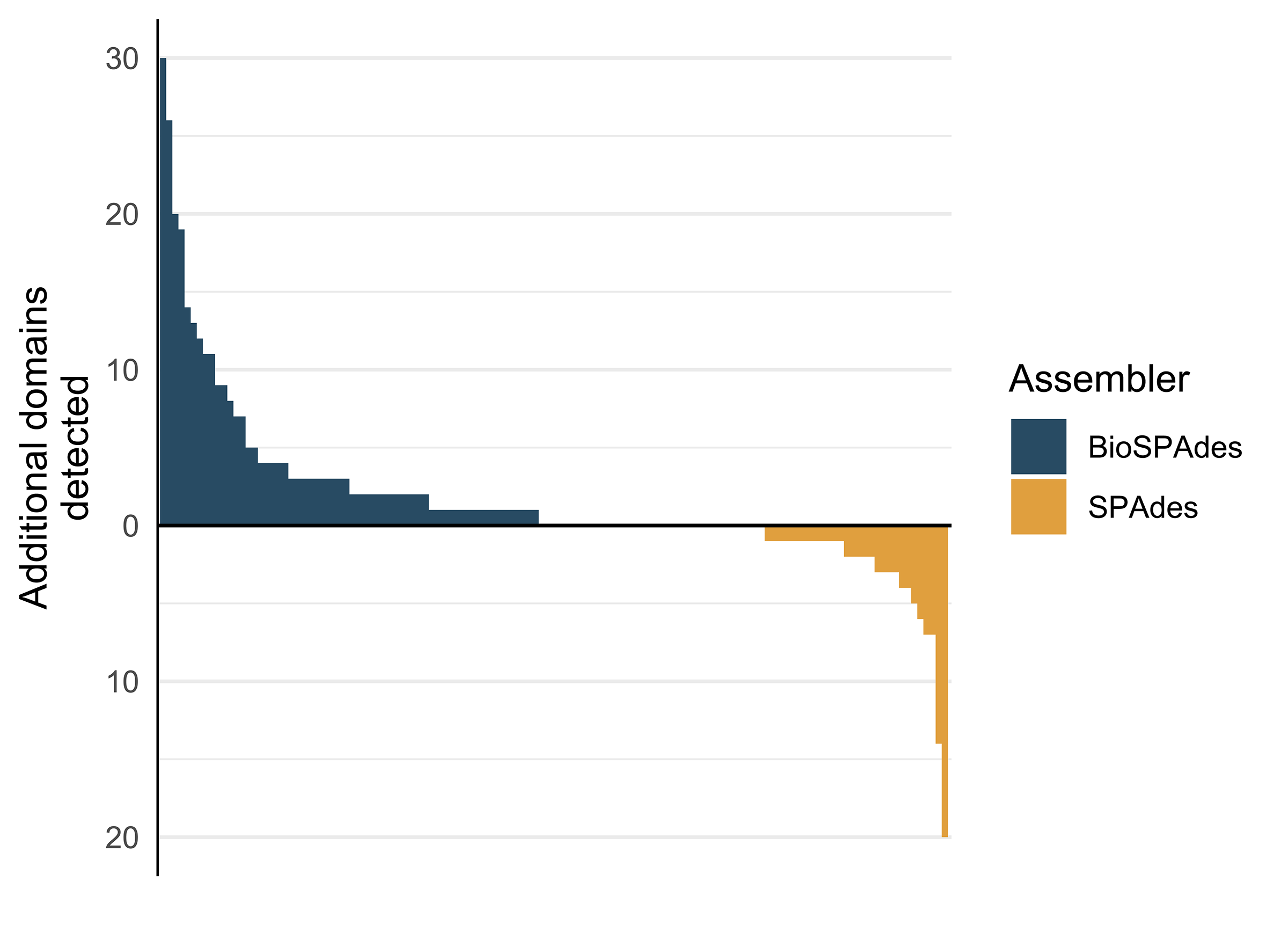

Supplement: Supplementary Figure 5 — Increase in the domain count of PKS and/or NRPS clusters recovered from the resequenced clones by assembly with either SPAdes or biosynthetic-SPAdes (bioSPAdes). Both assemblers showed cases where more domains were predicted within individual BGCs, however bioSPAdes performed better overall with respect to the overall total number of additional domains predicted. [file Image_5.TIF]

a.

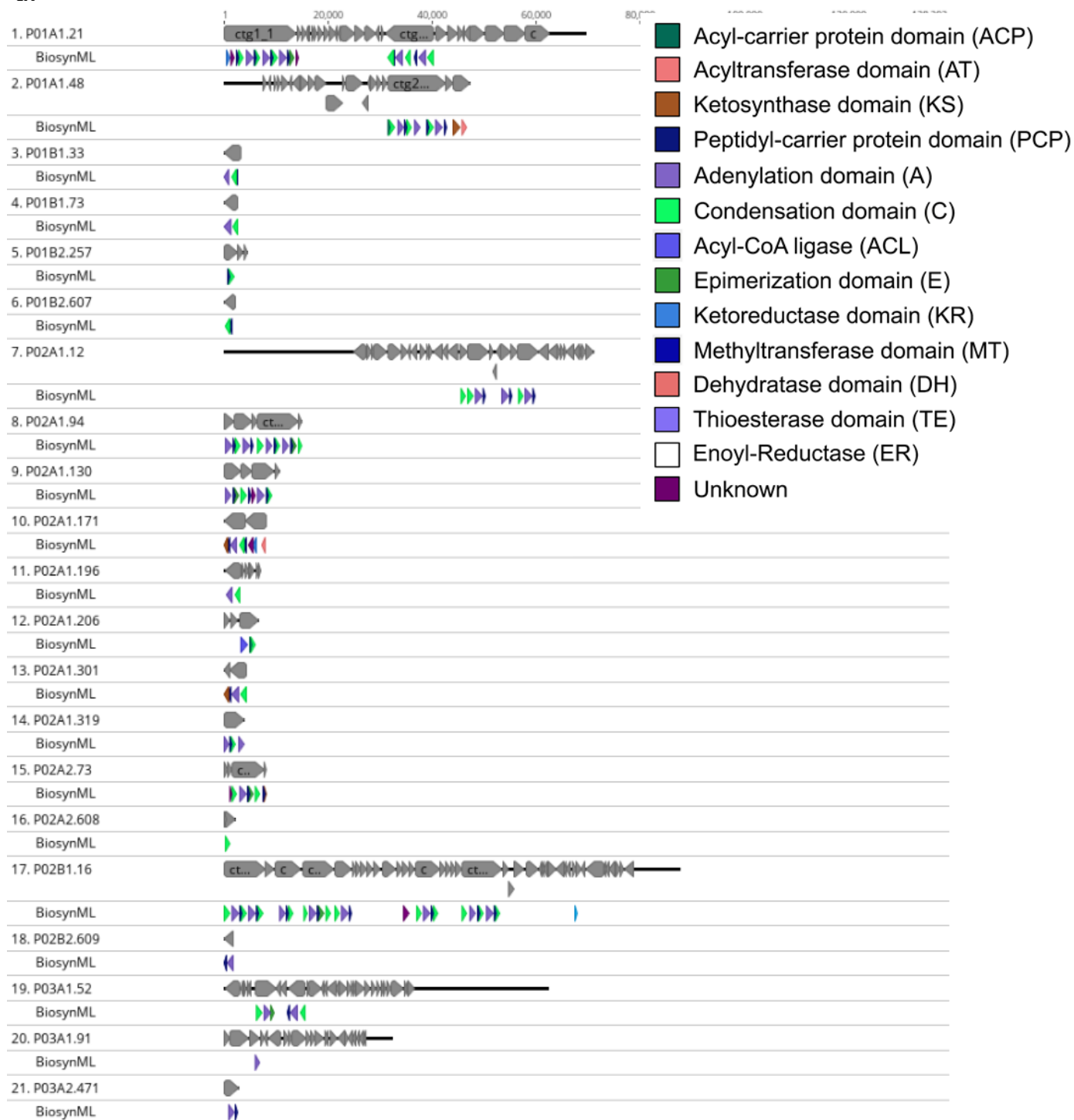

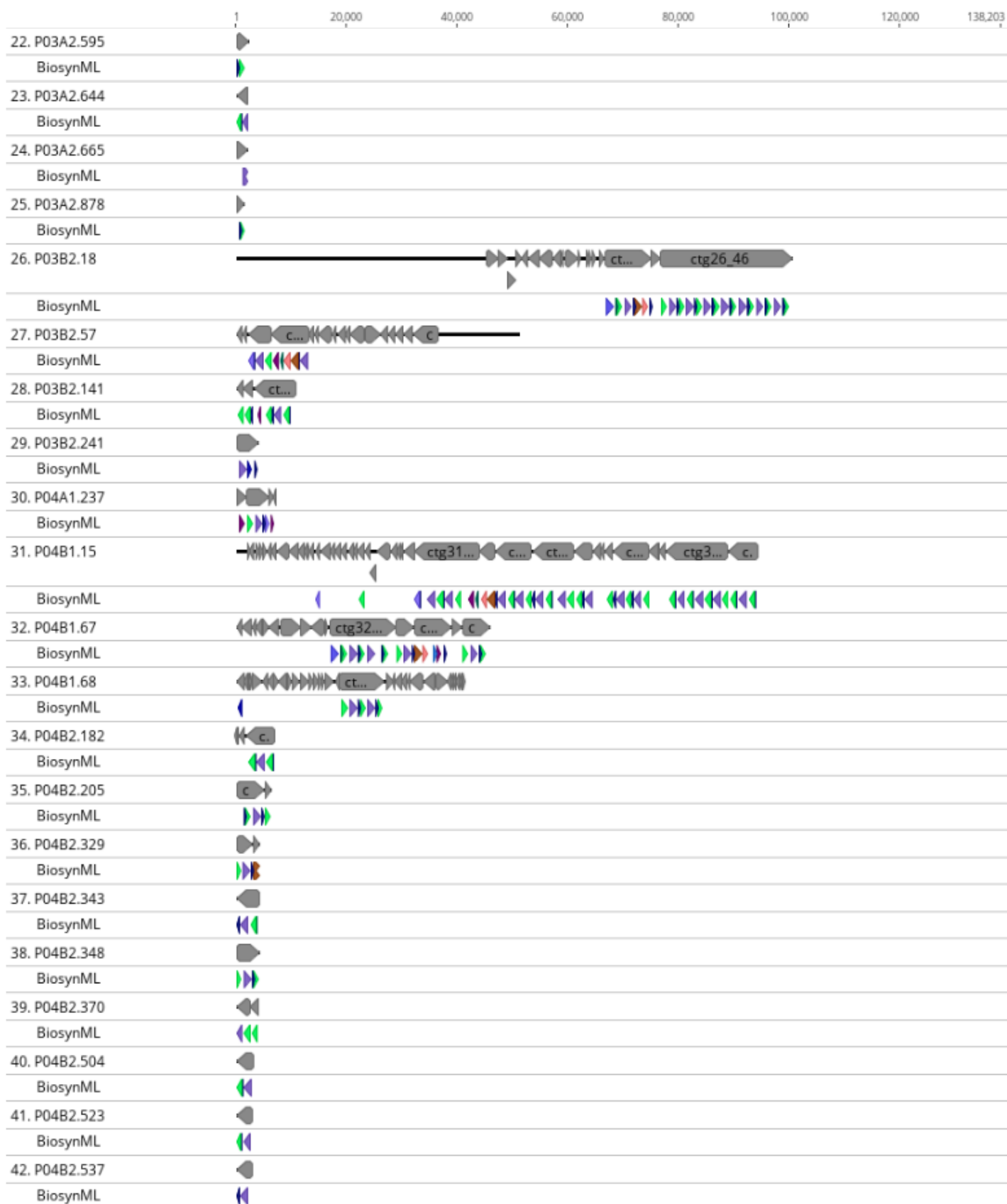

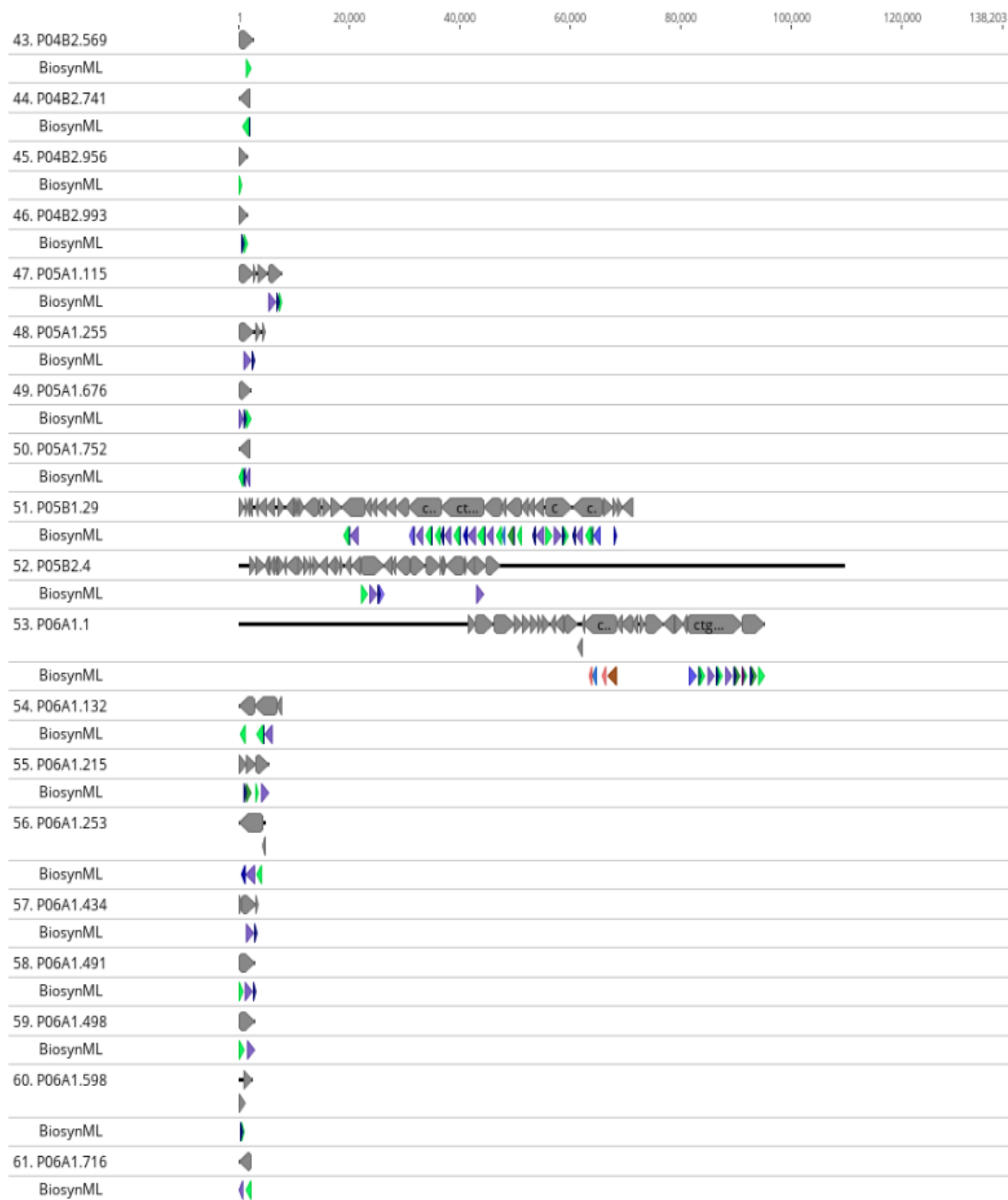

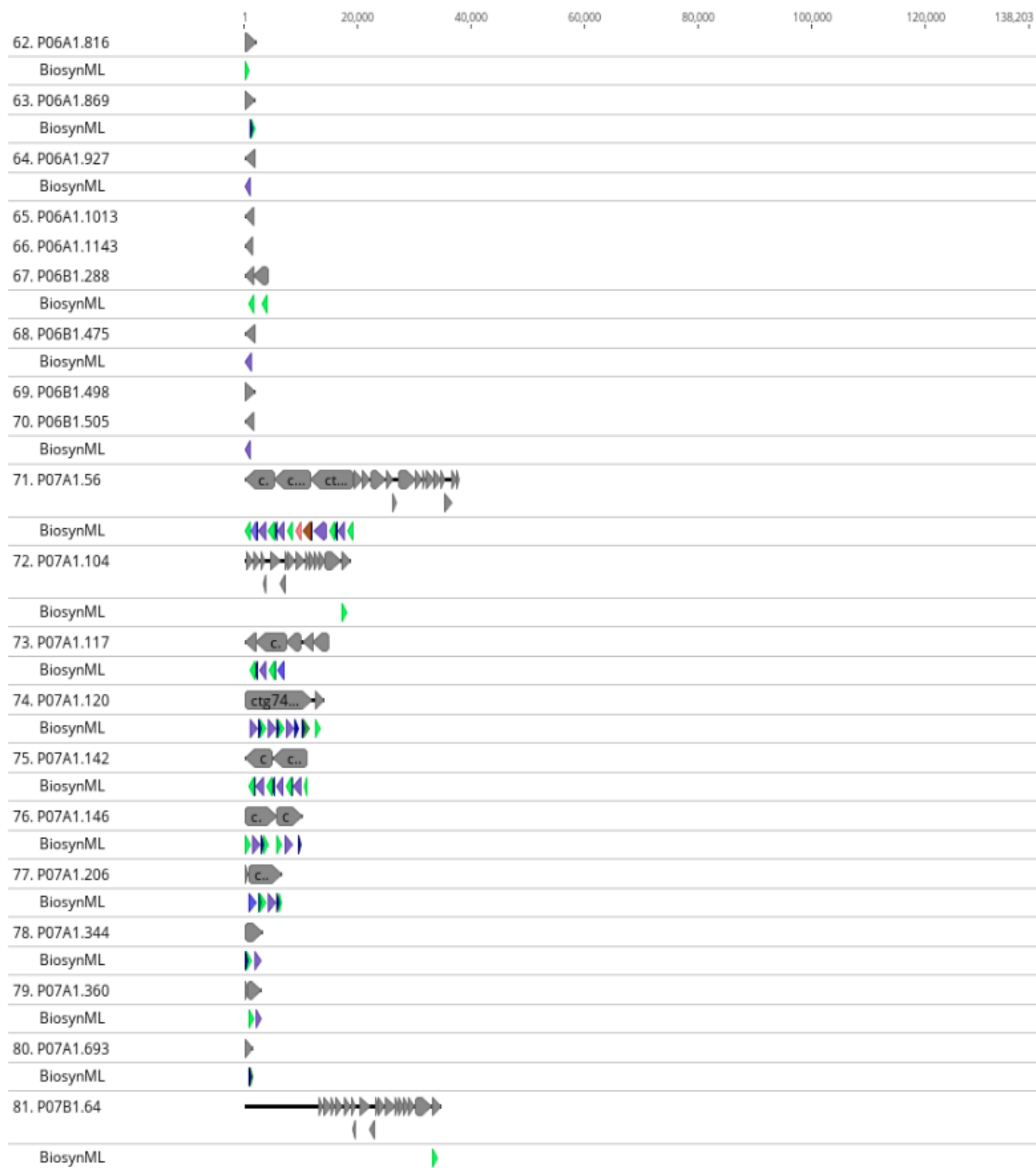

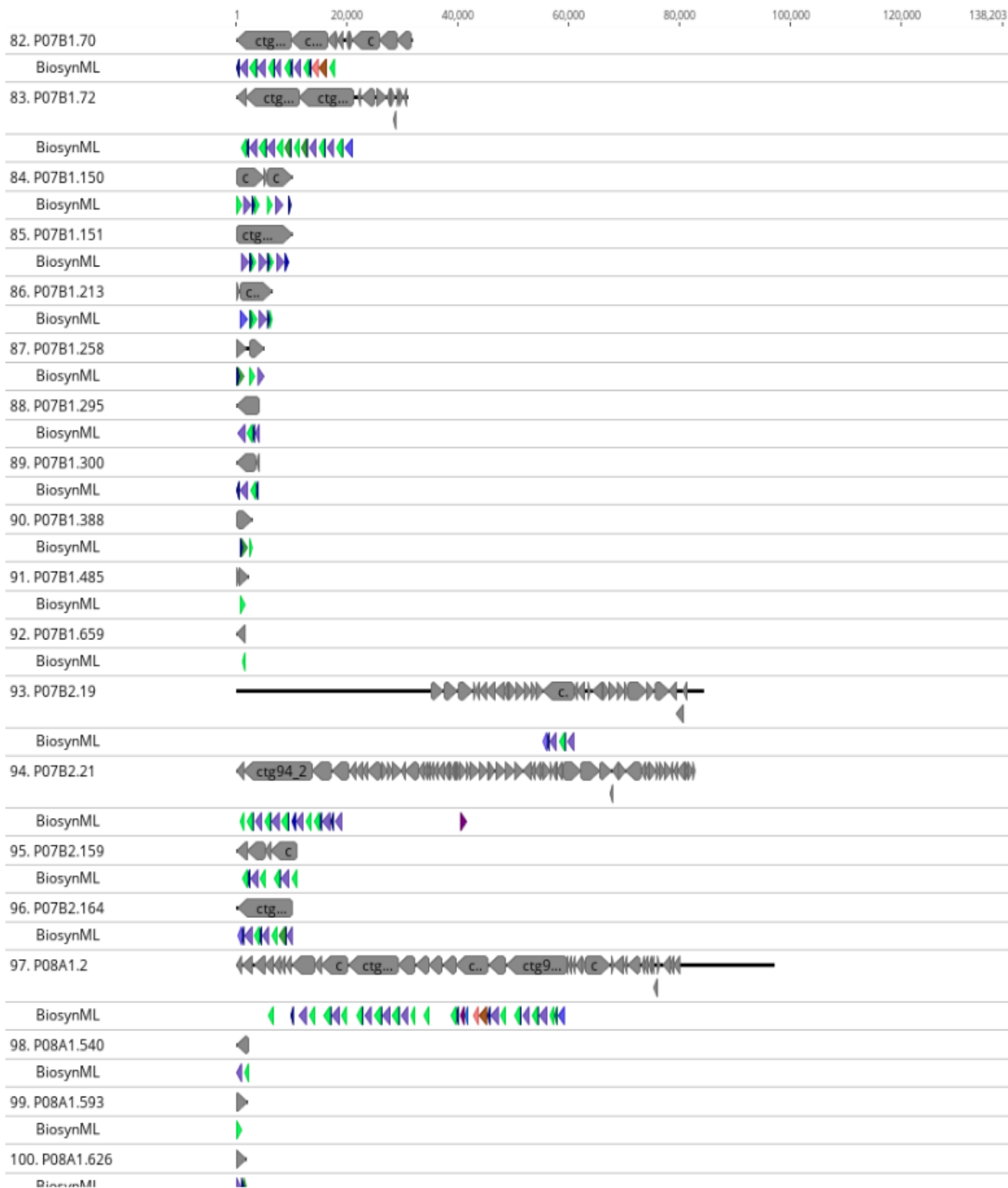

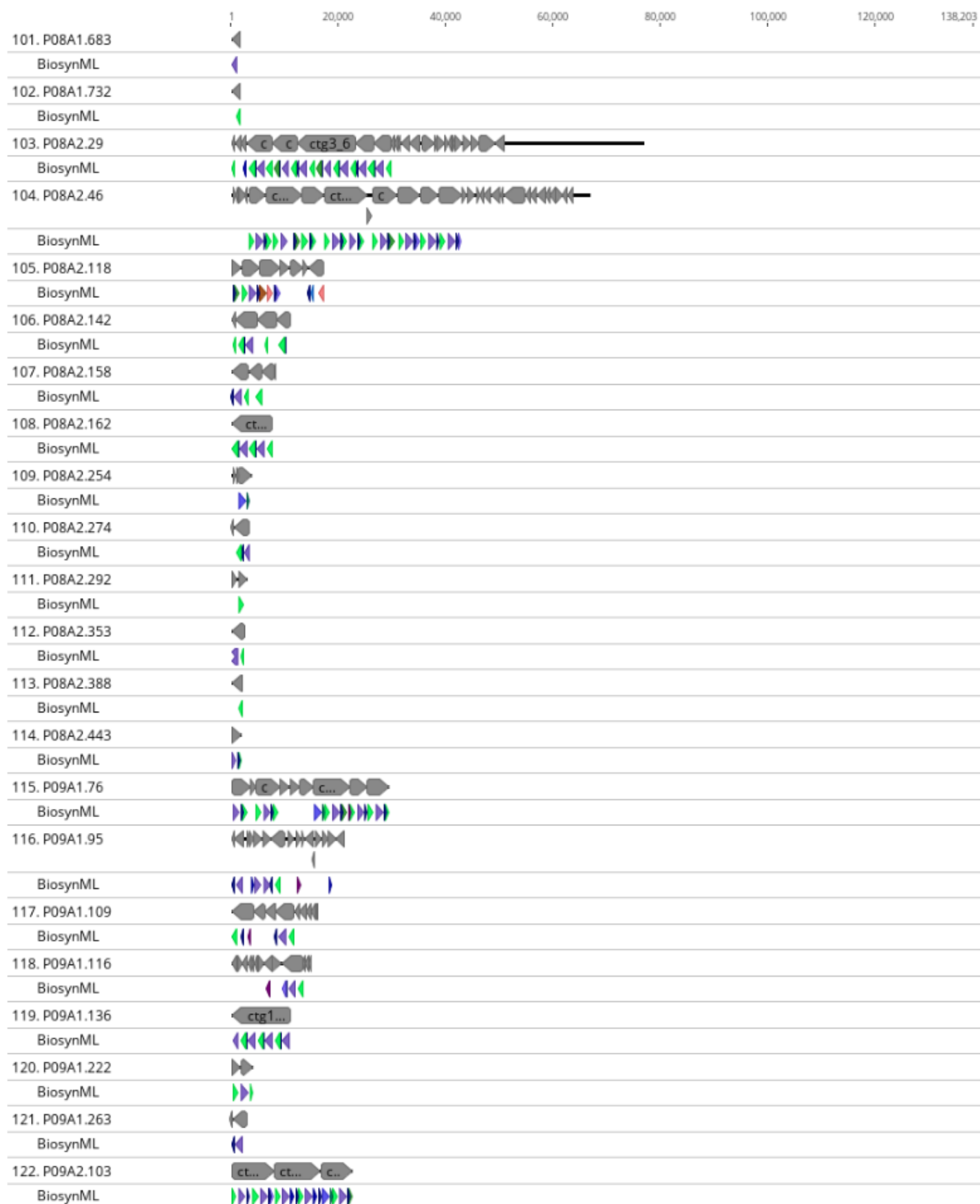

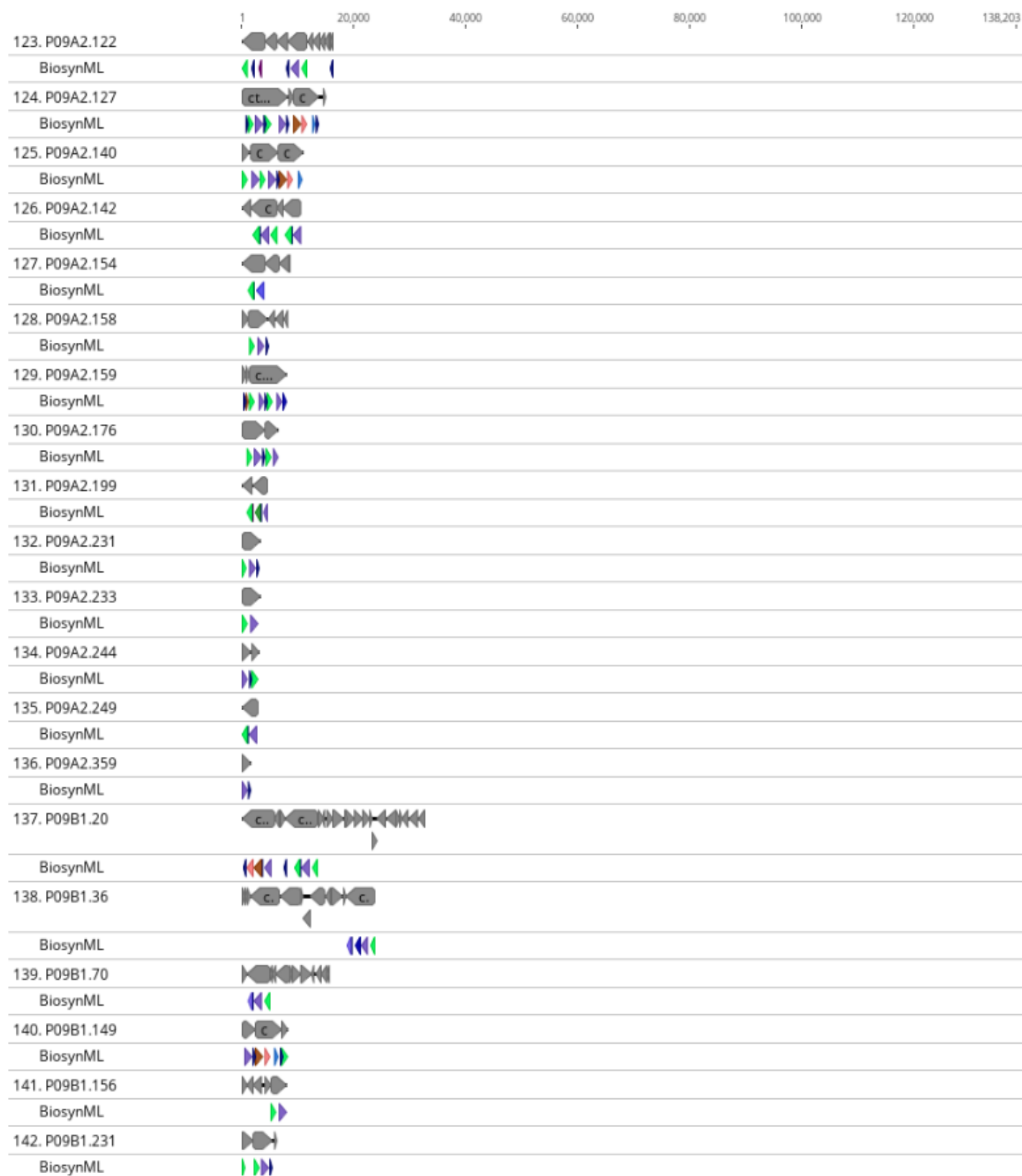

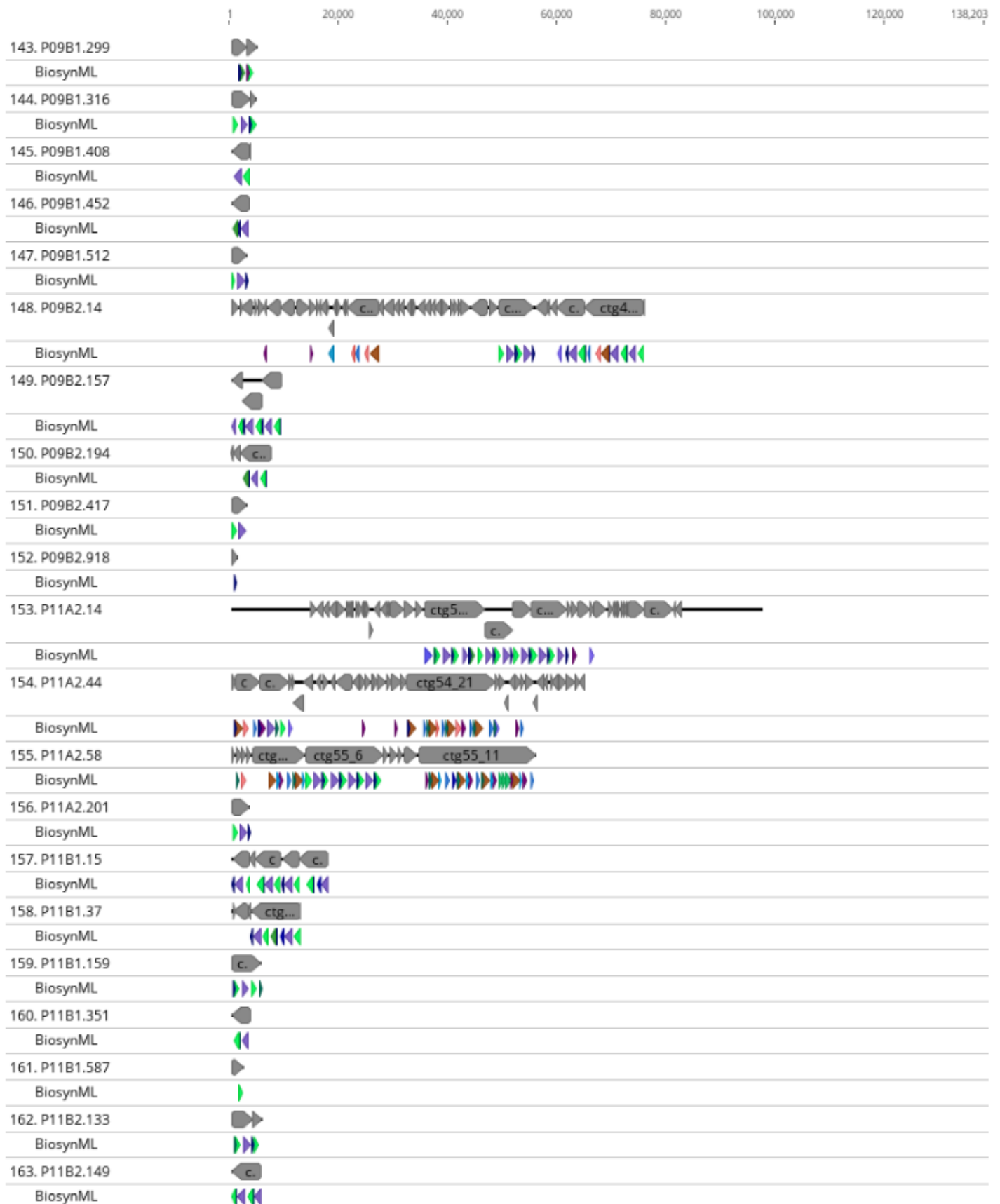

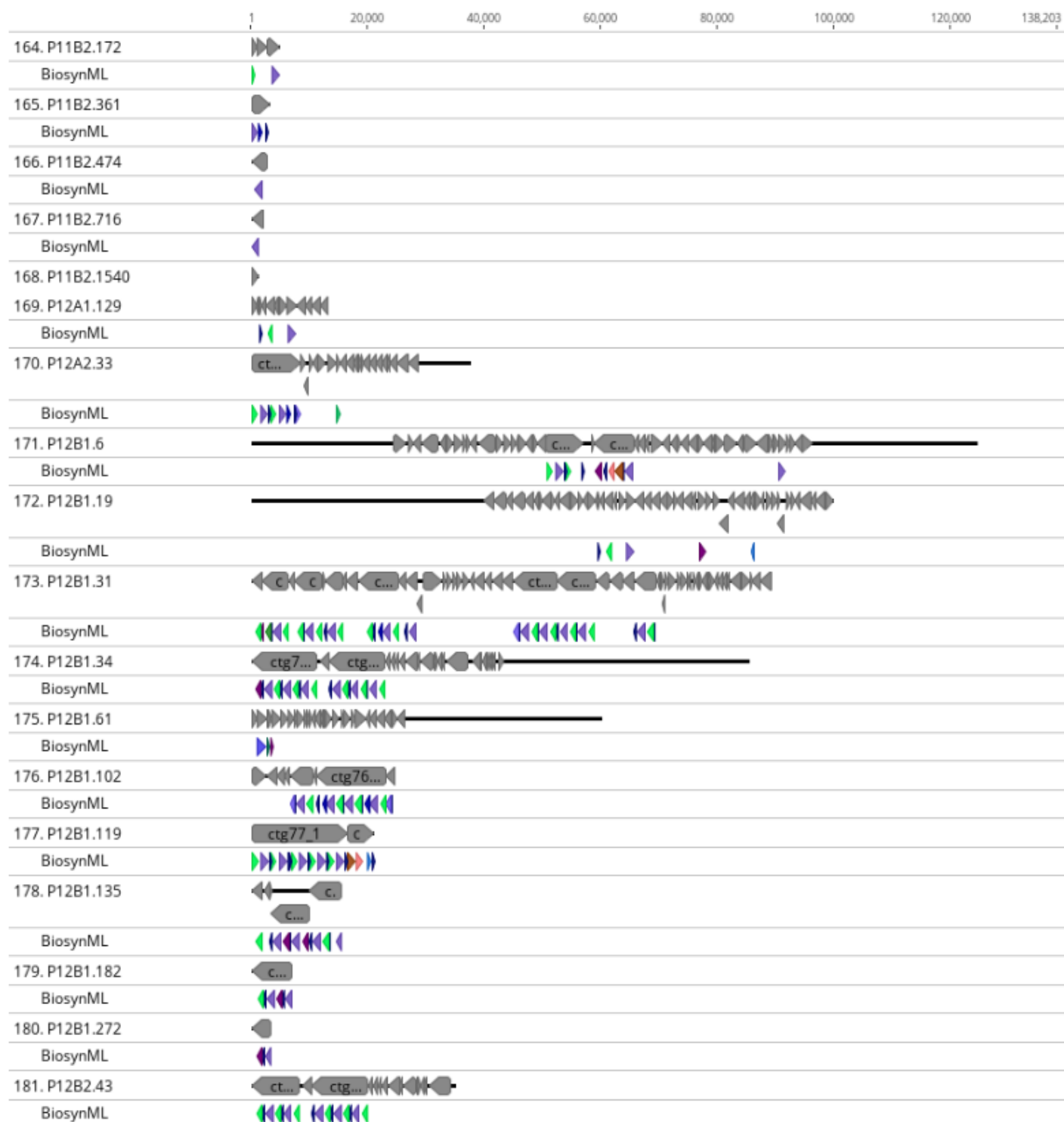

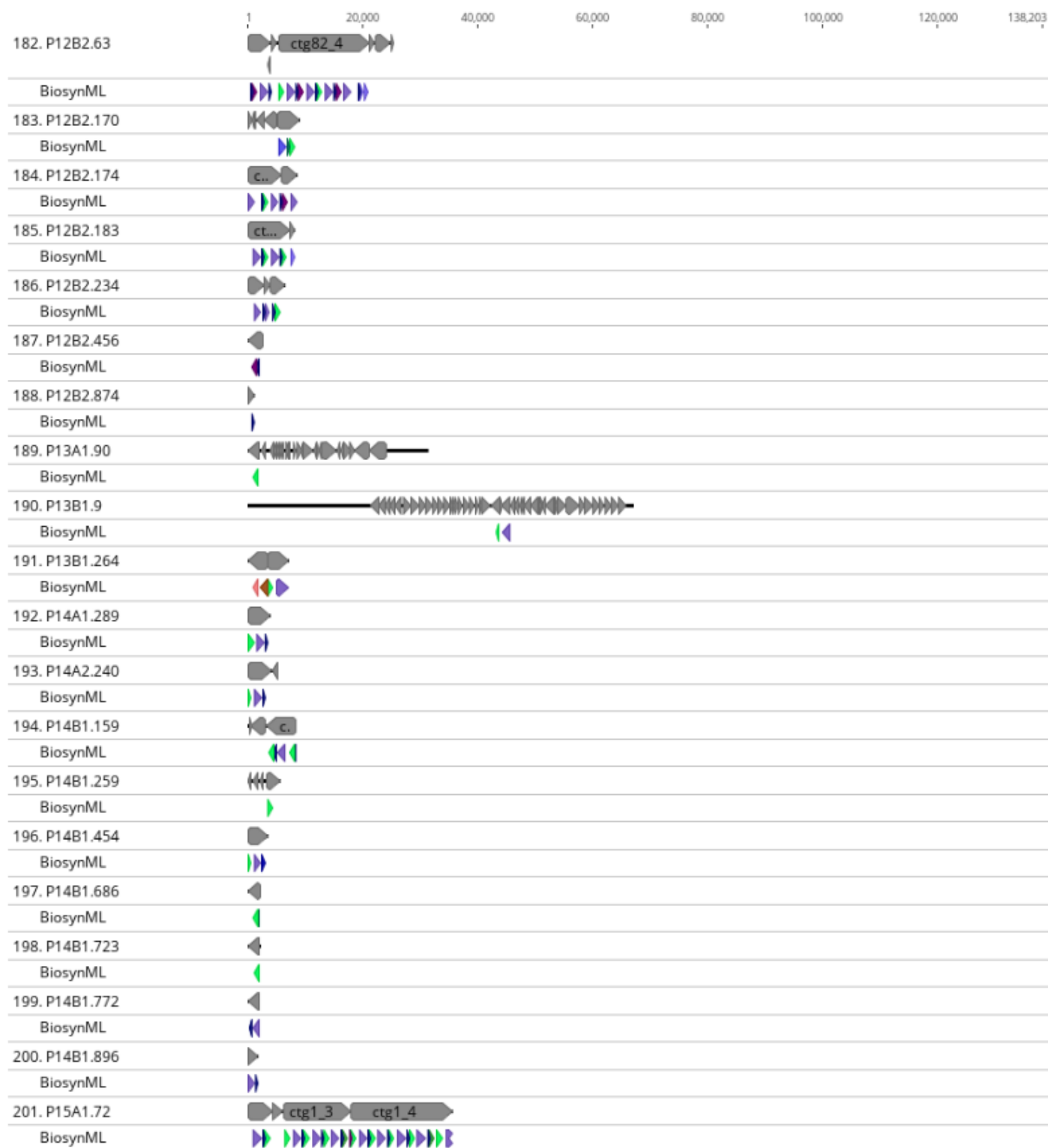

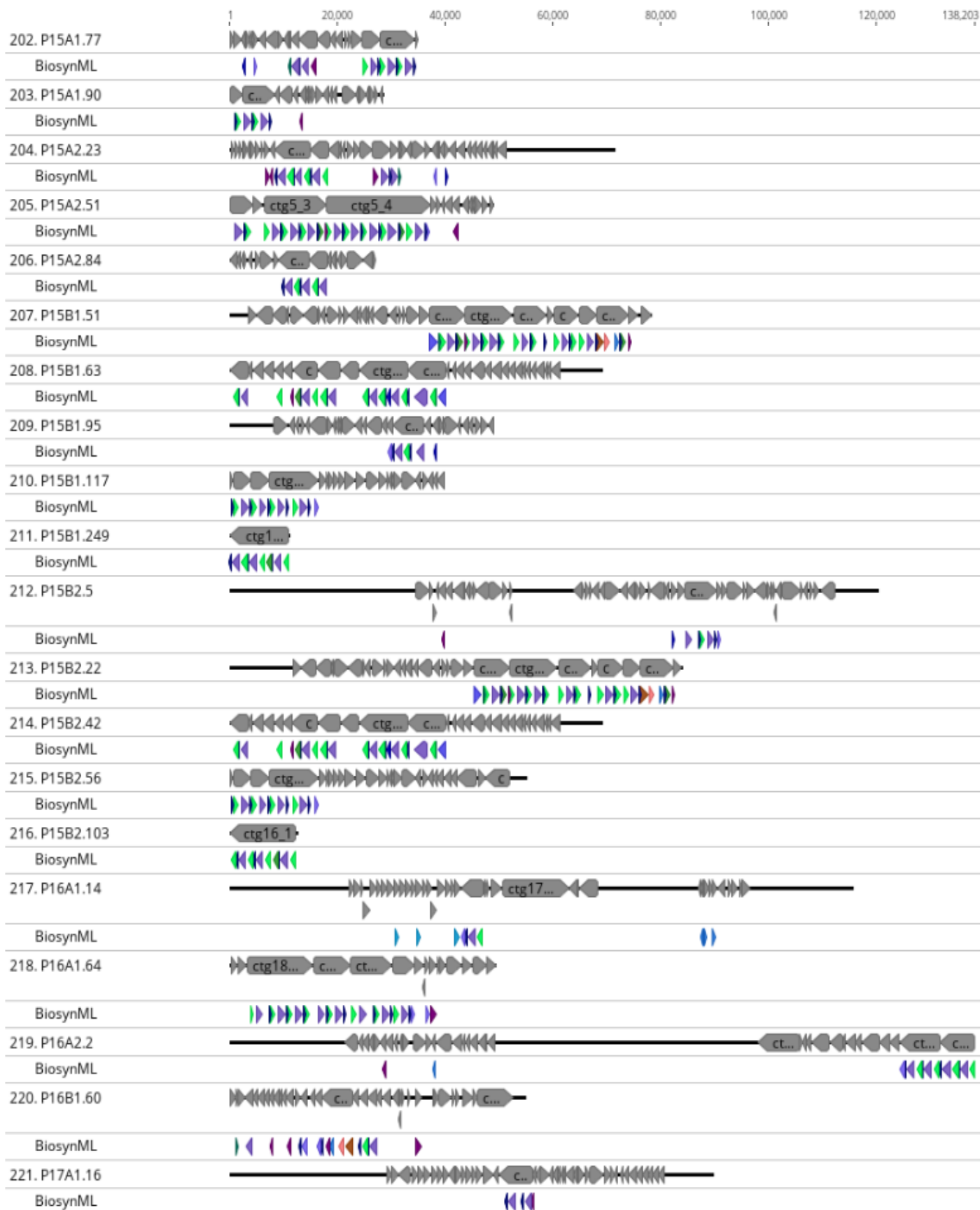

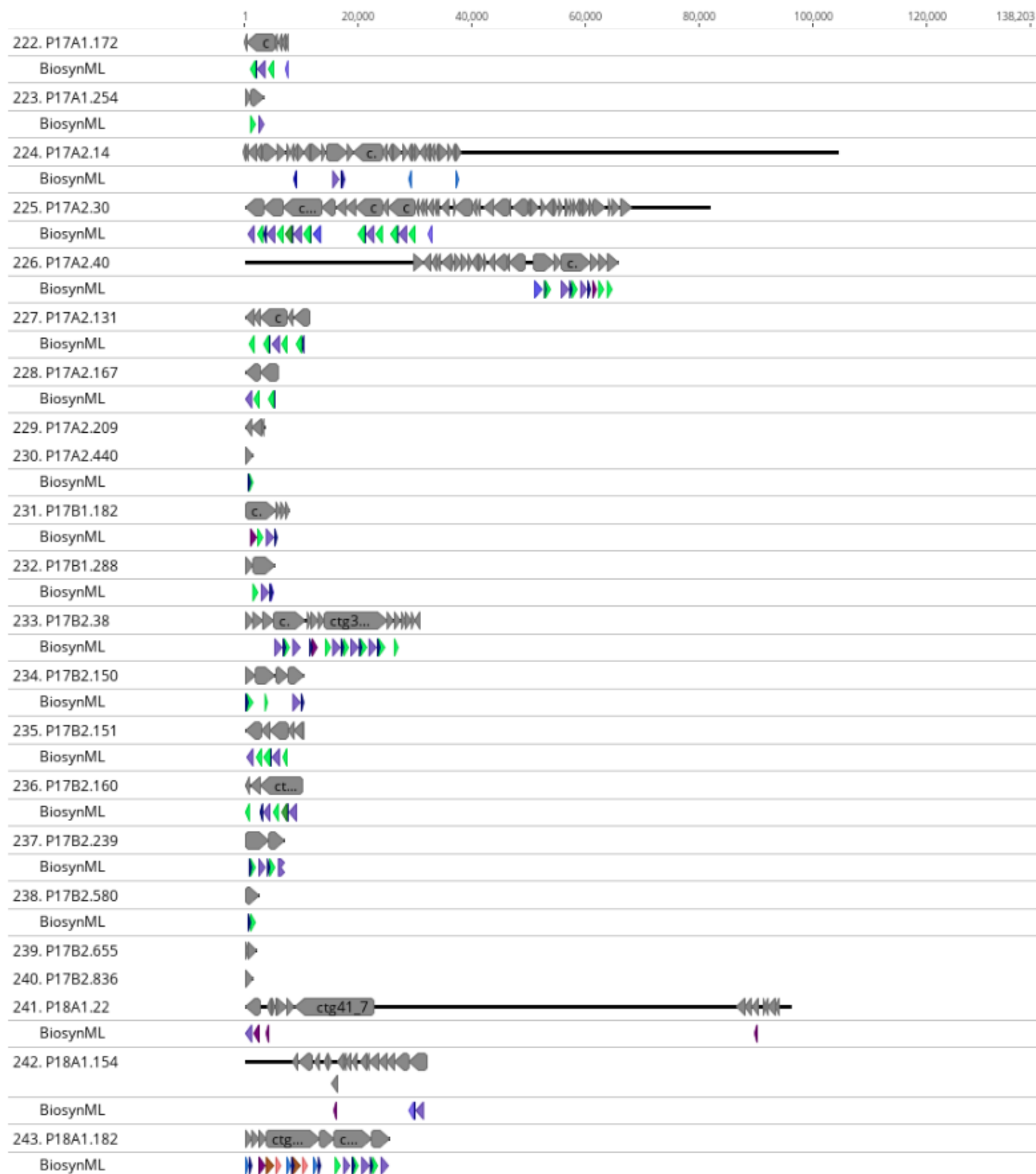

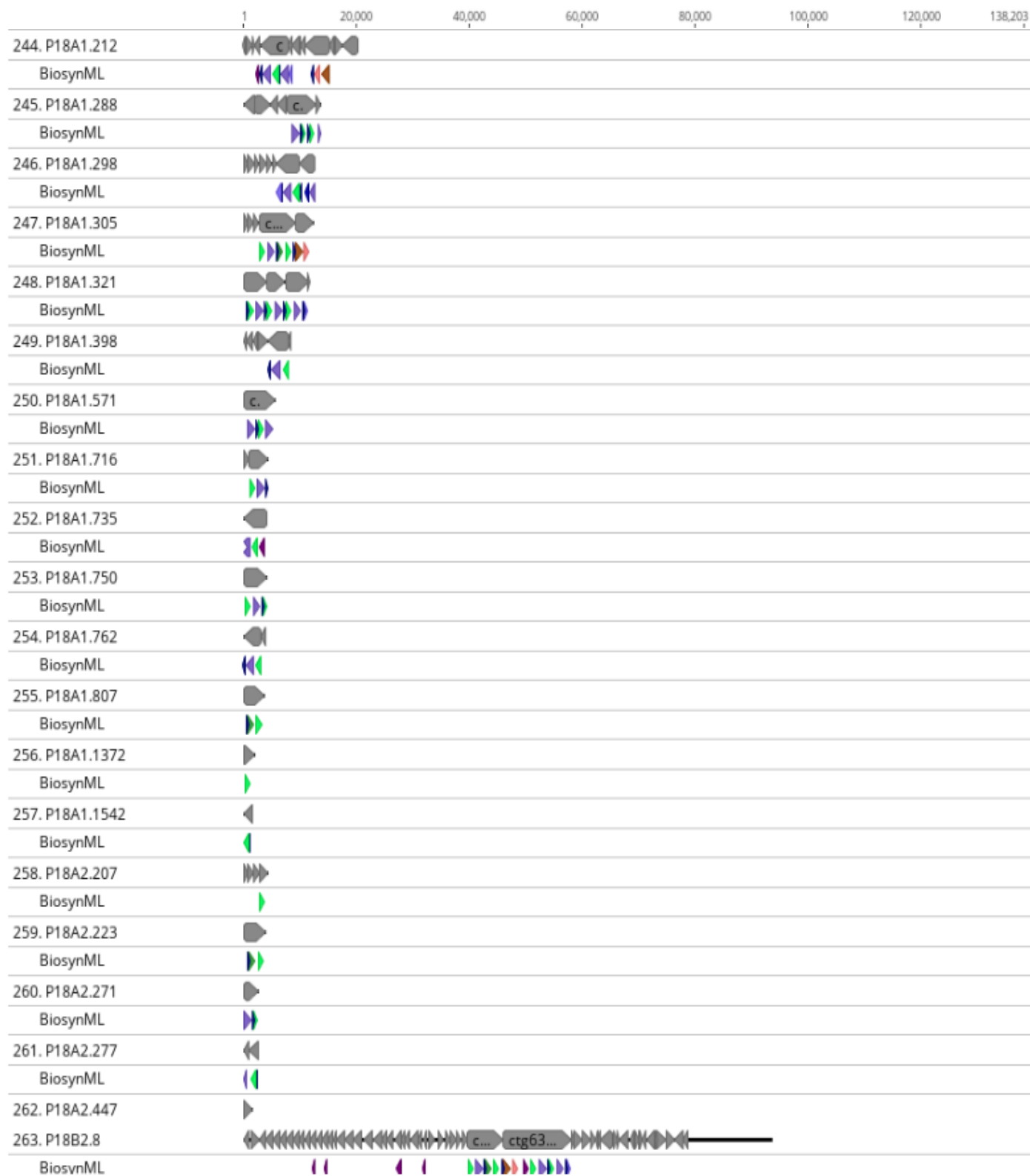

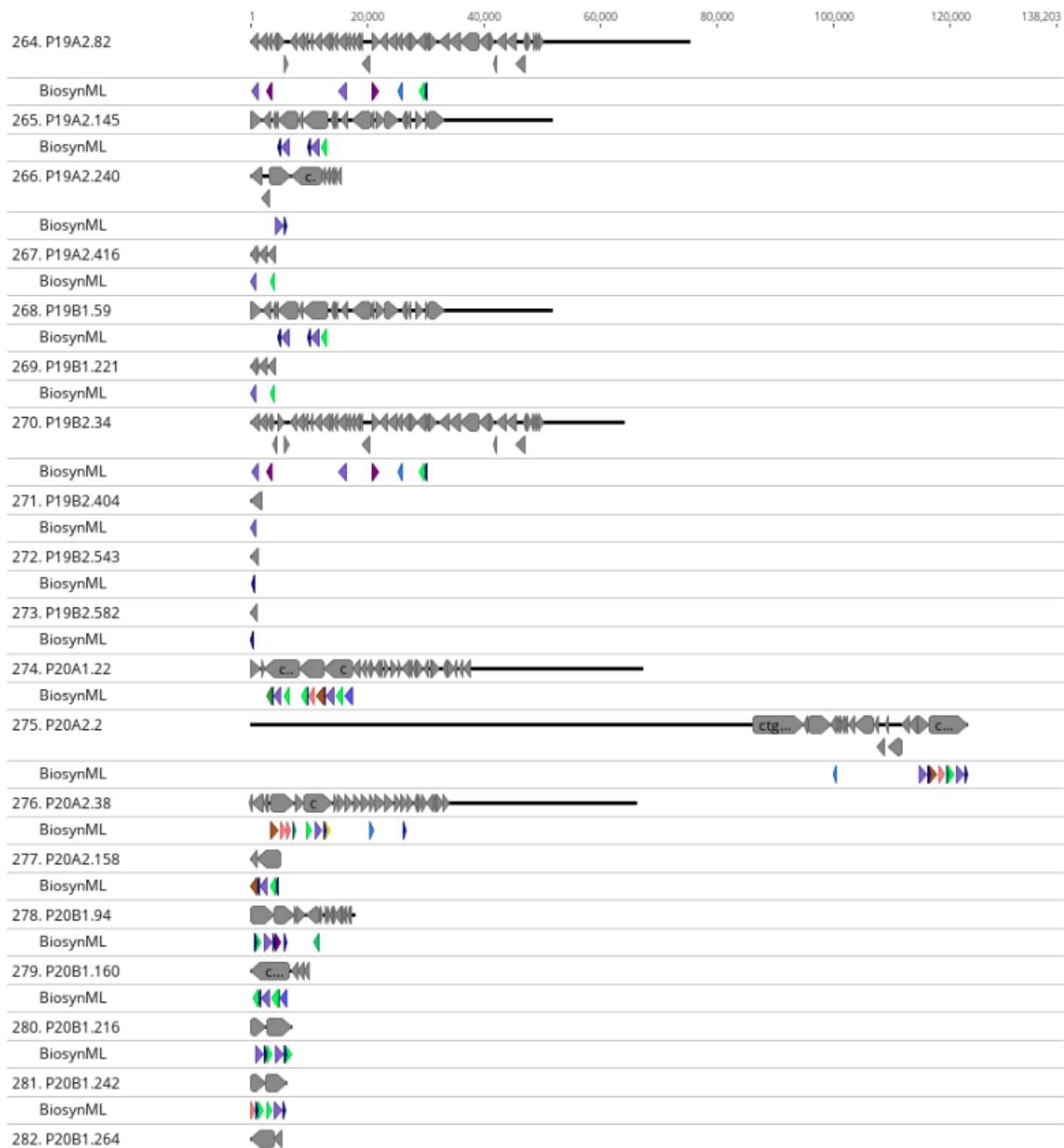

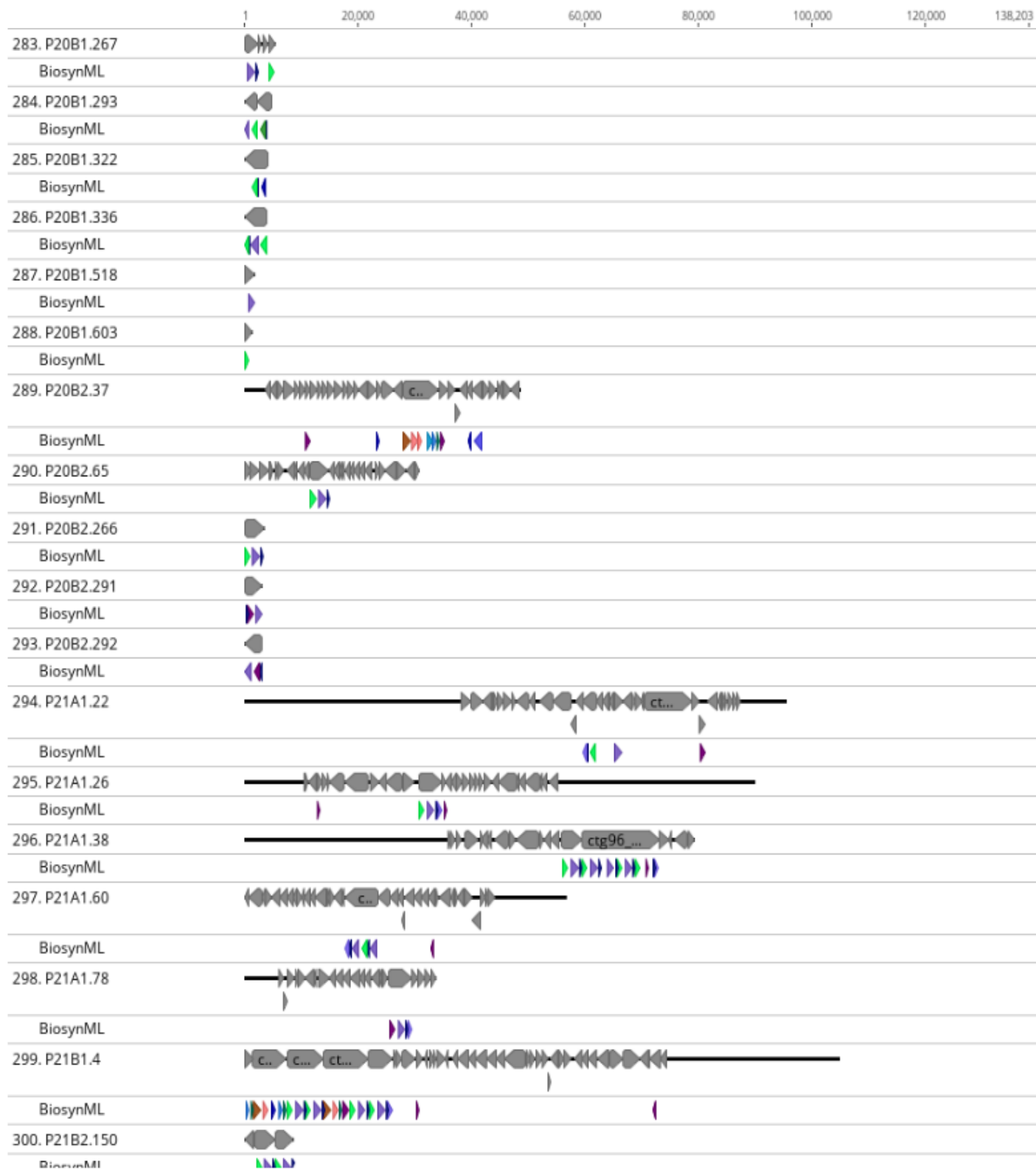

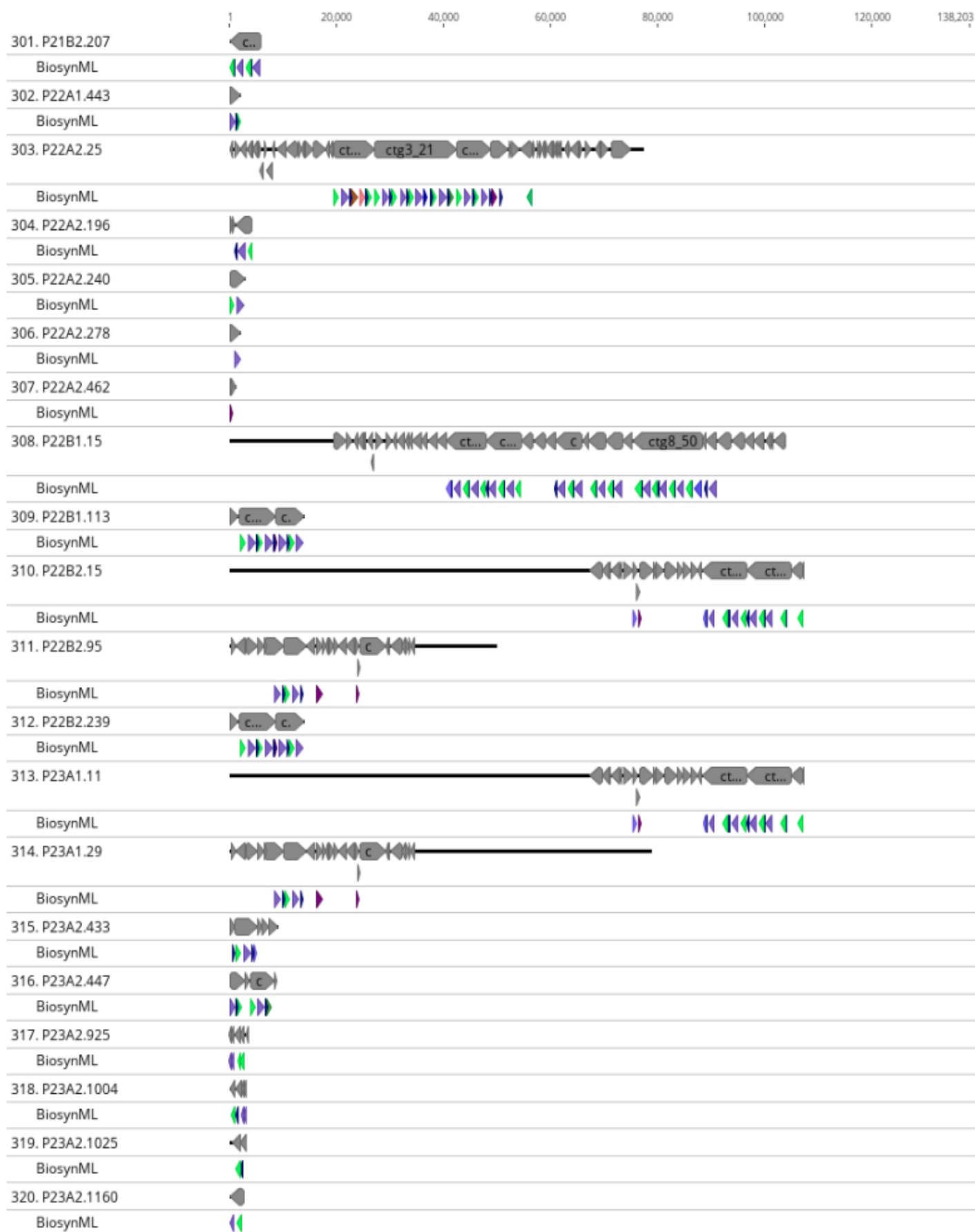

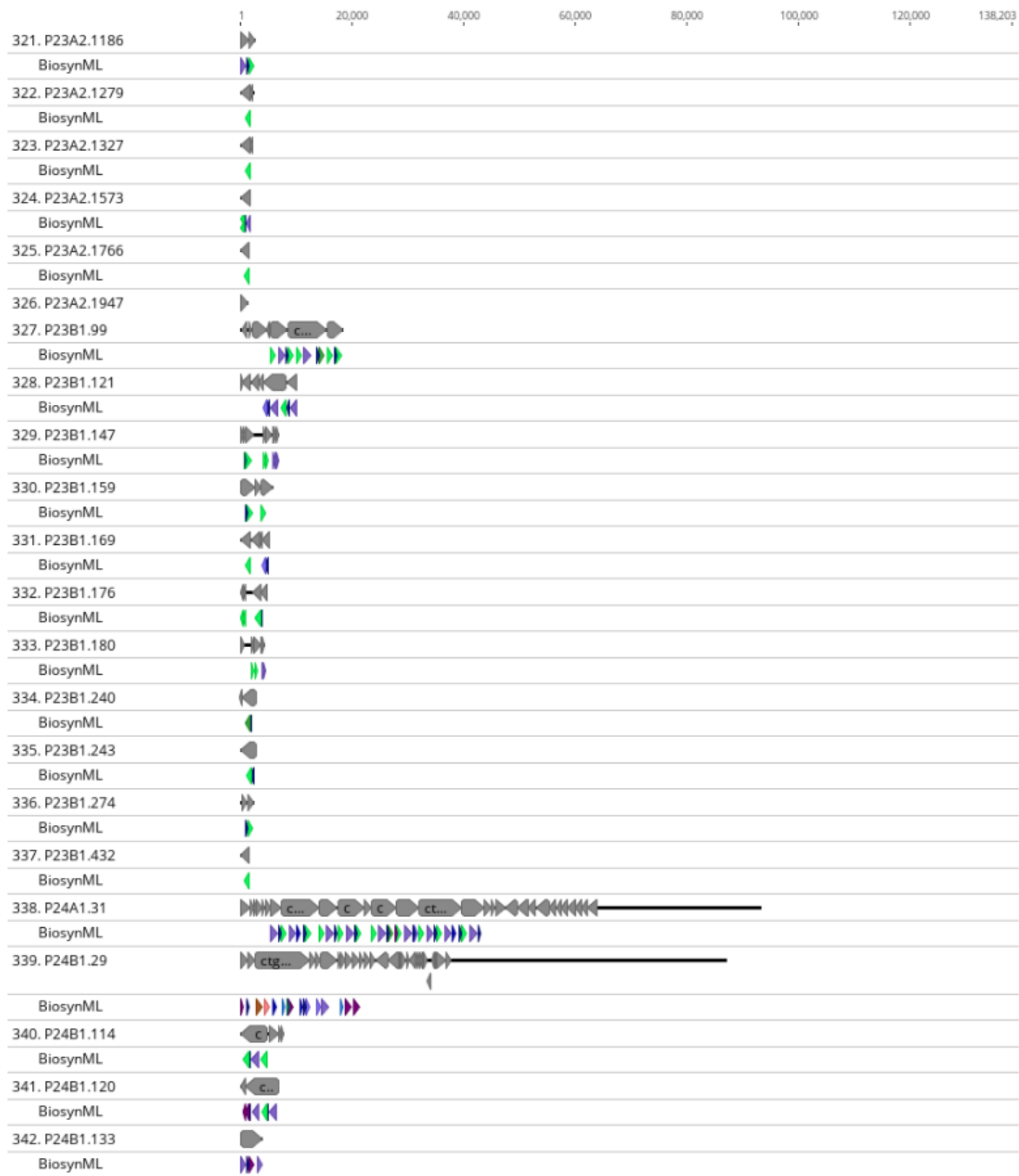

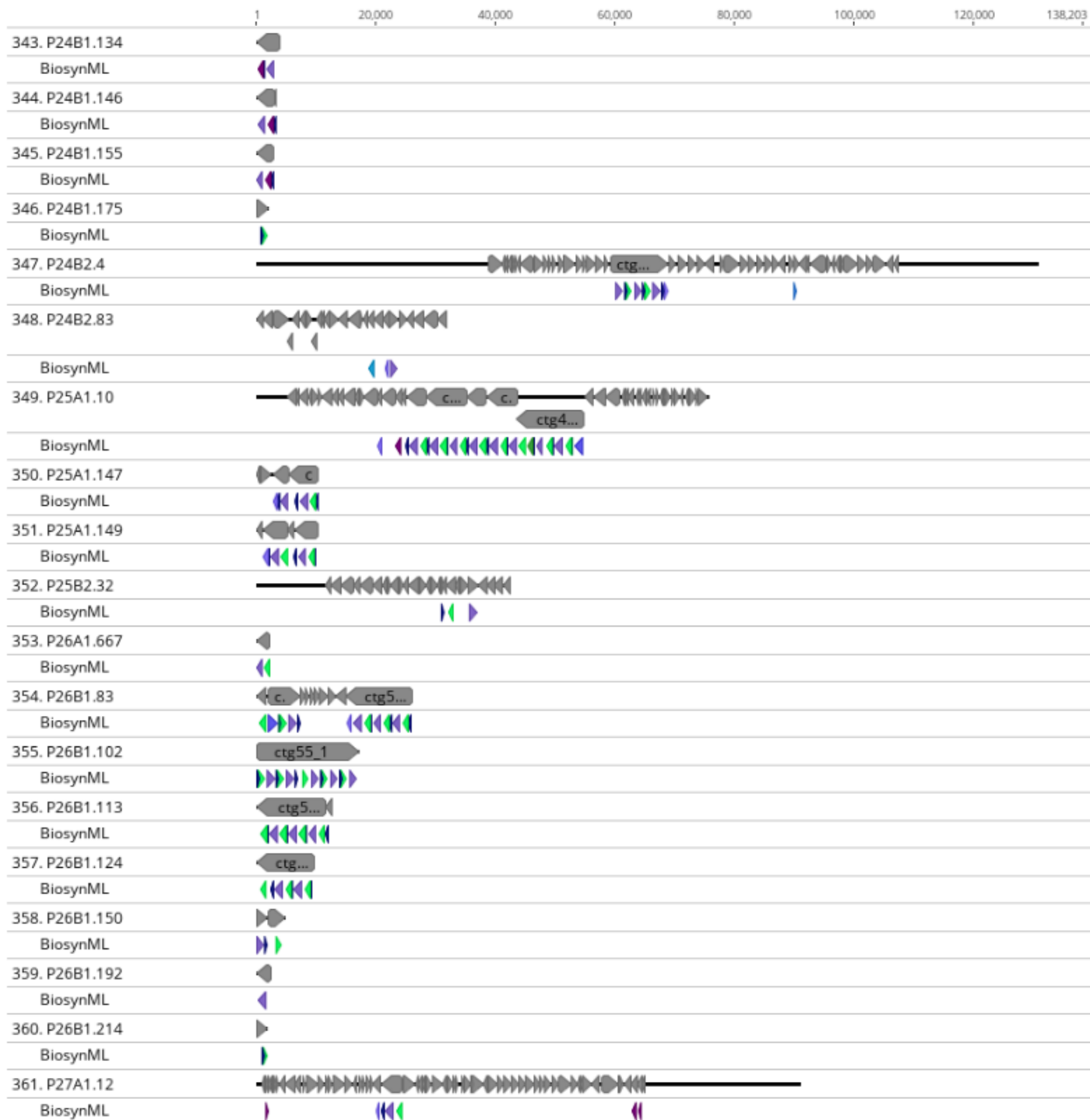

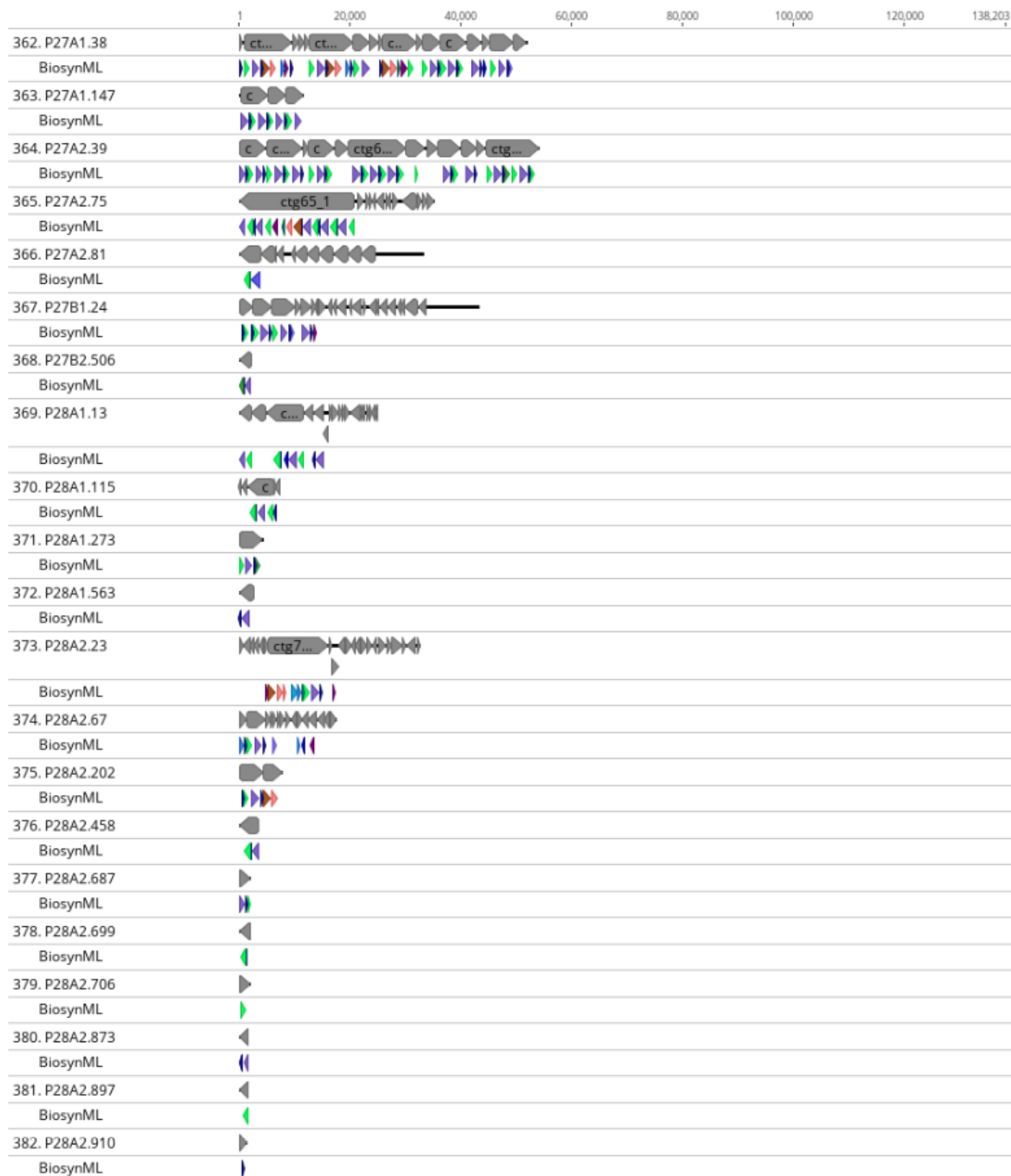

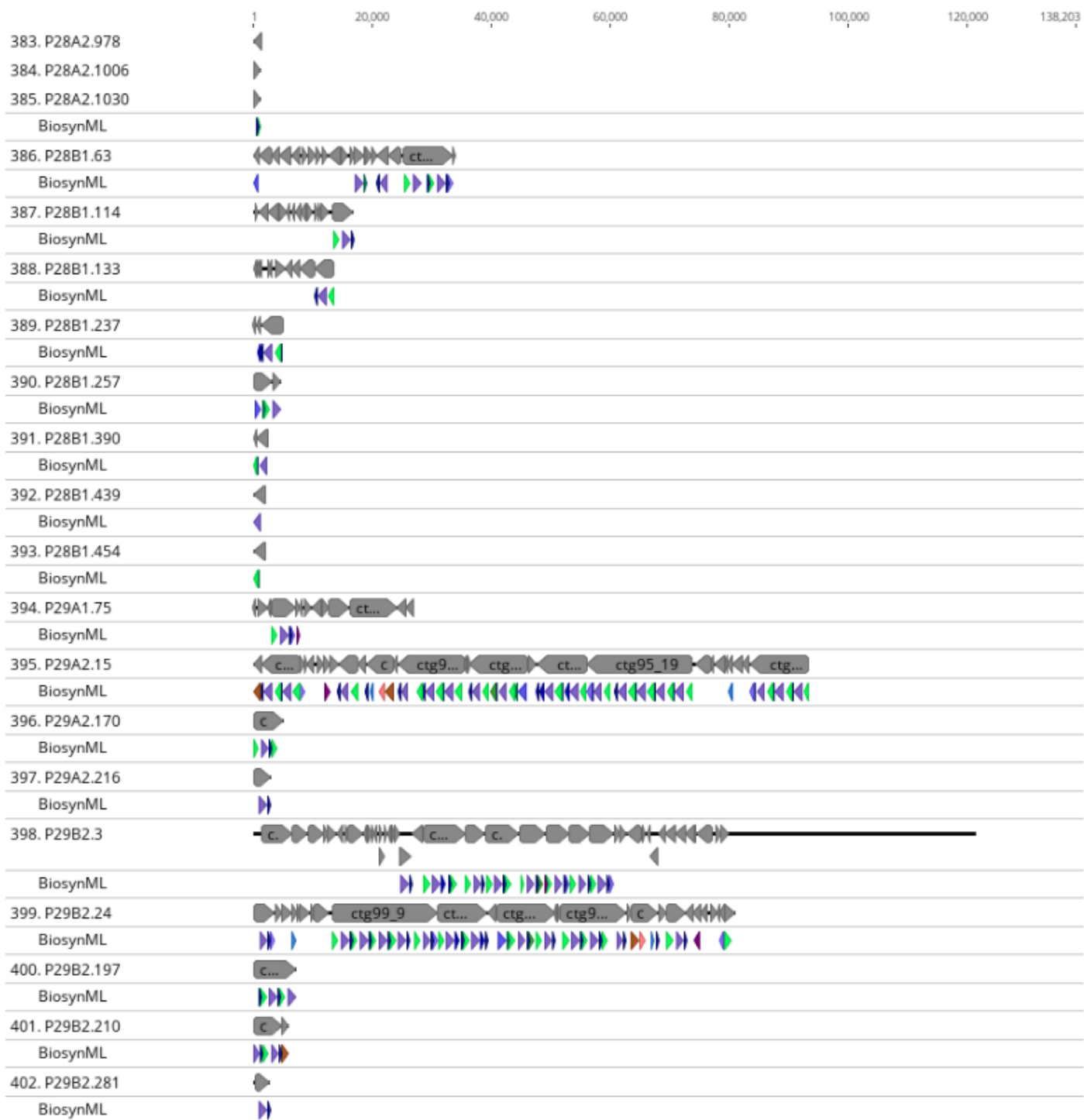

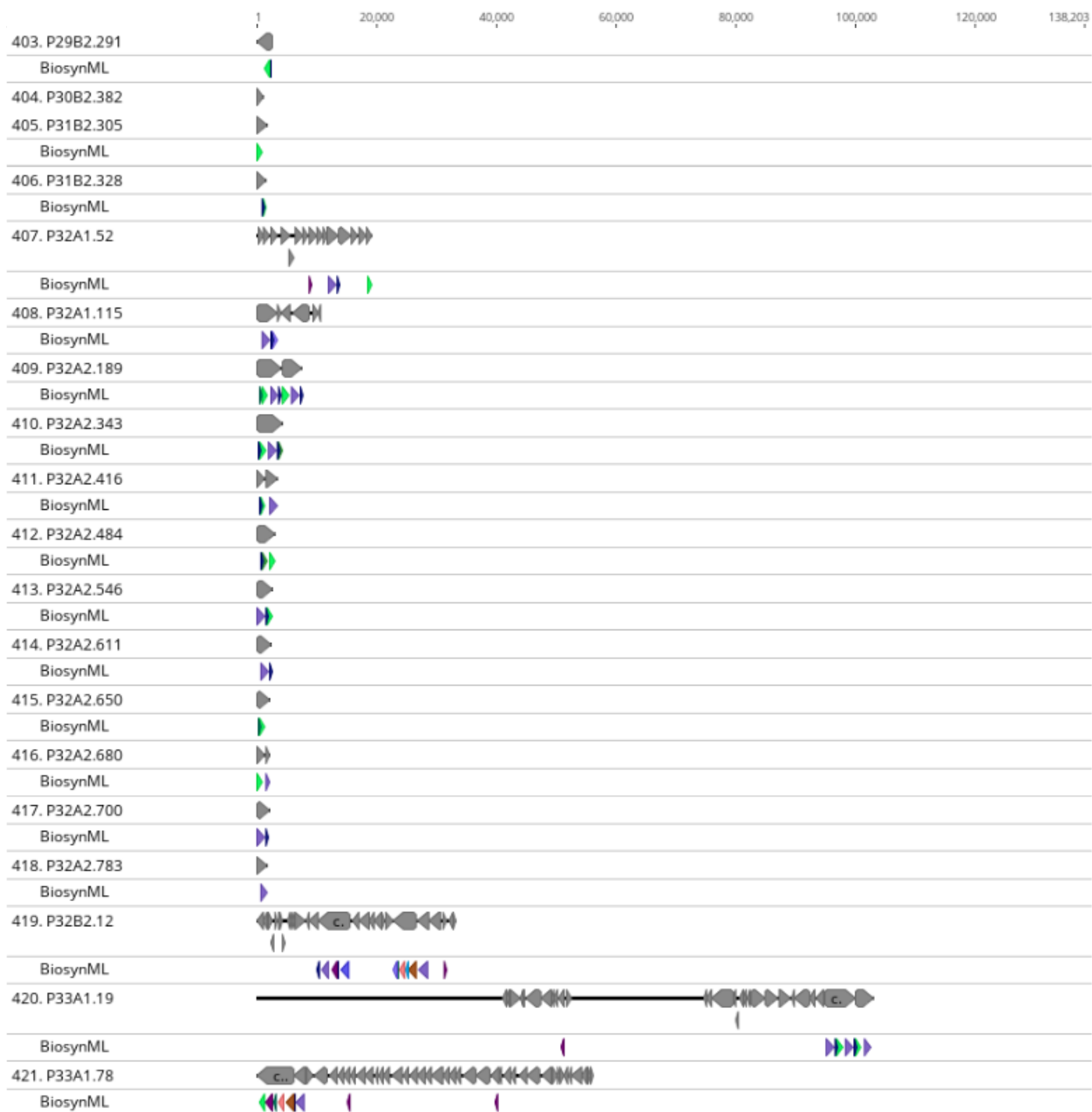

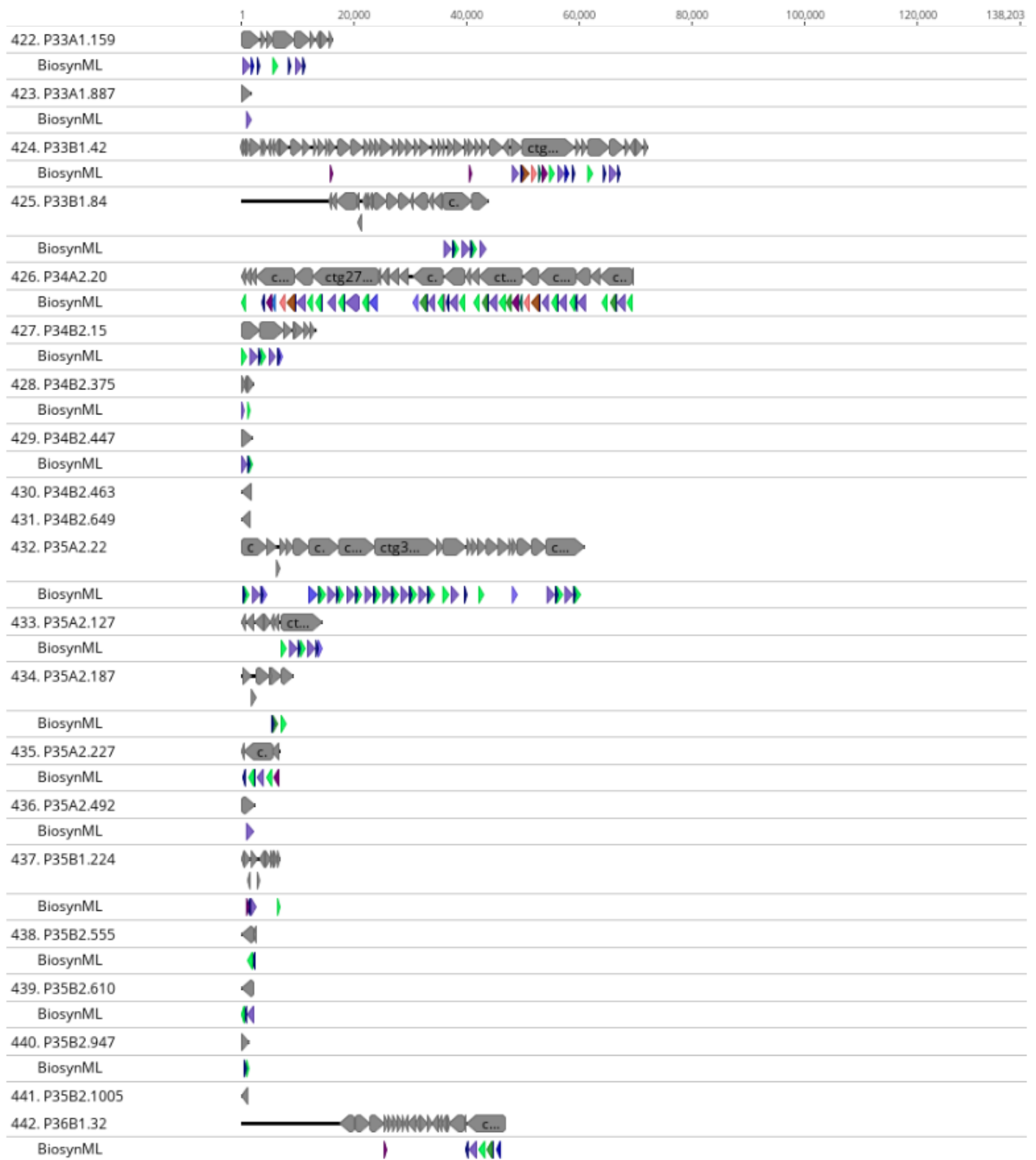

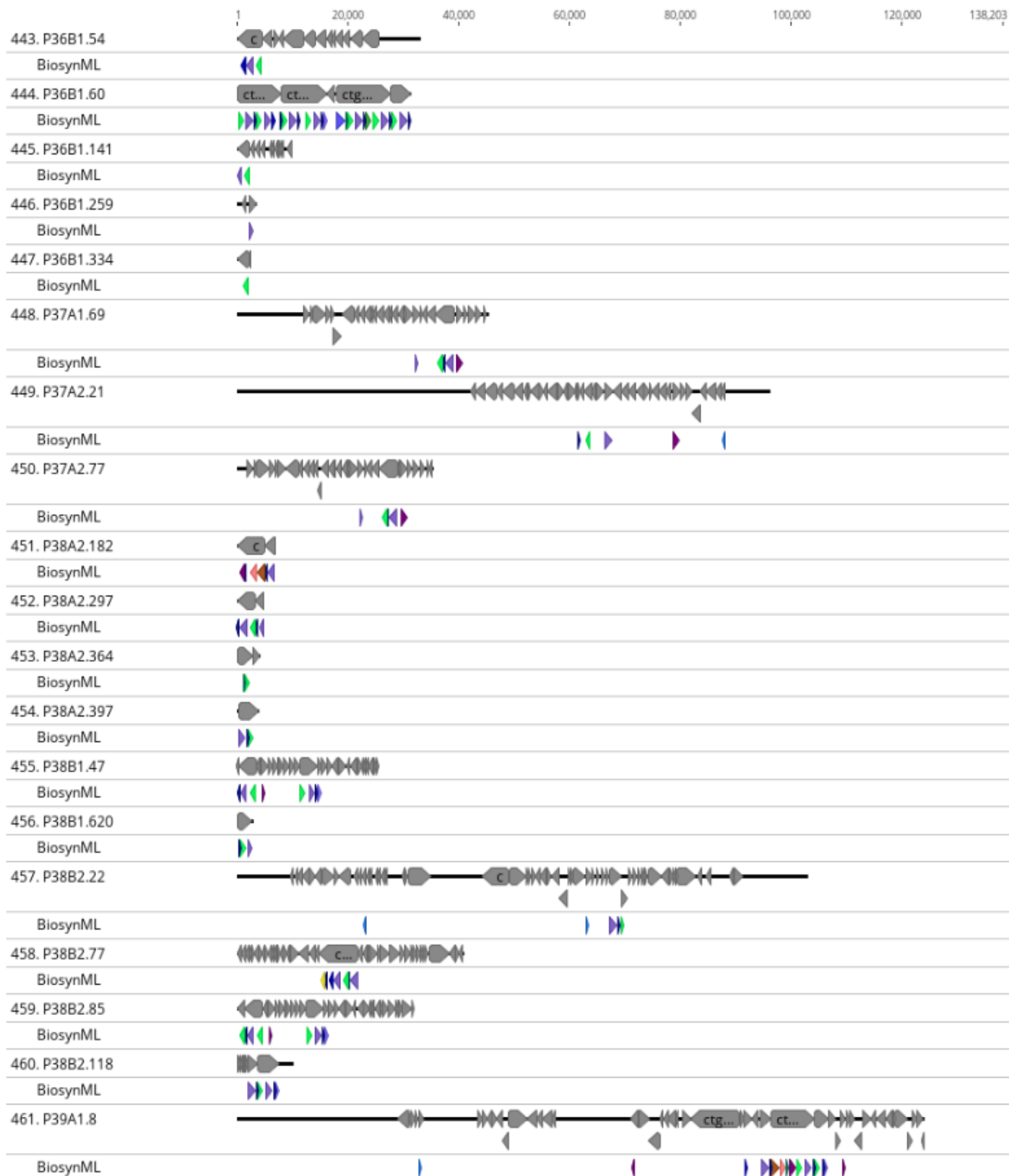

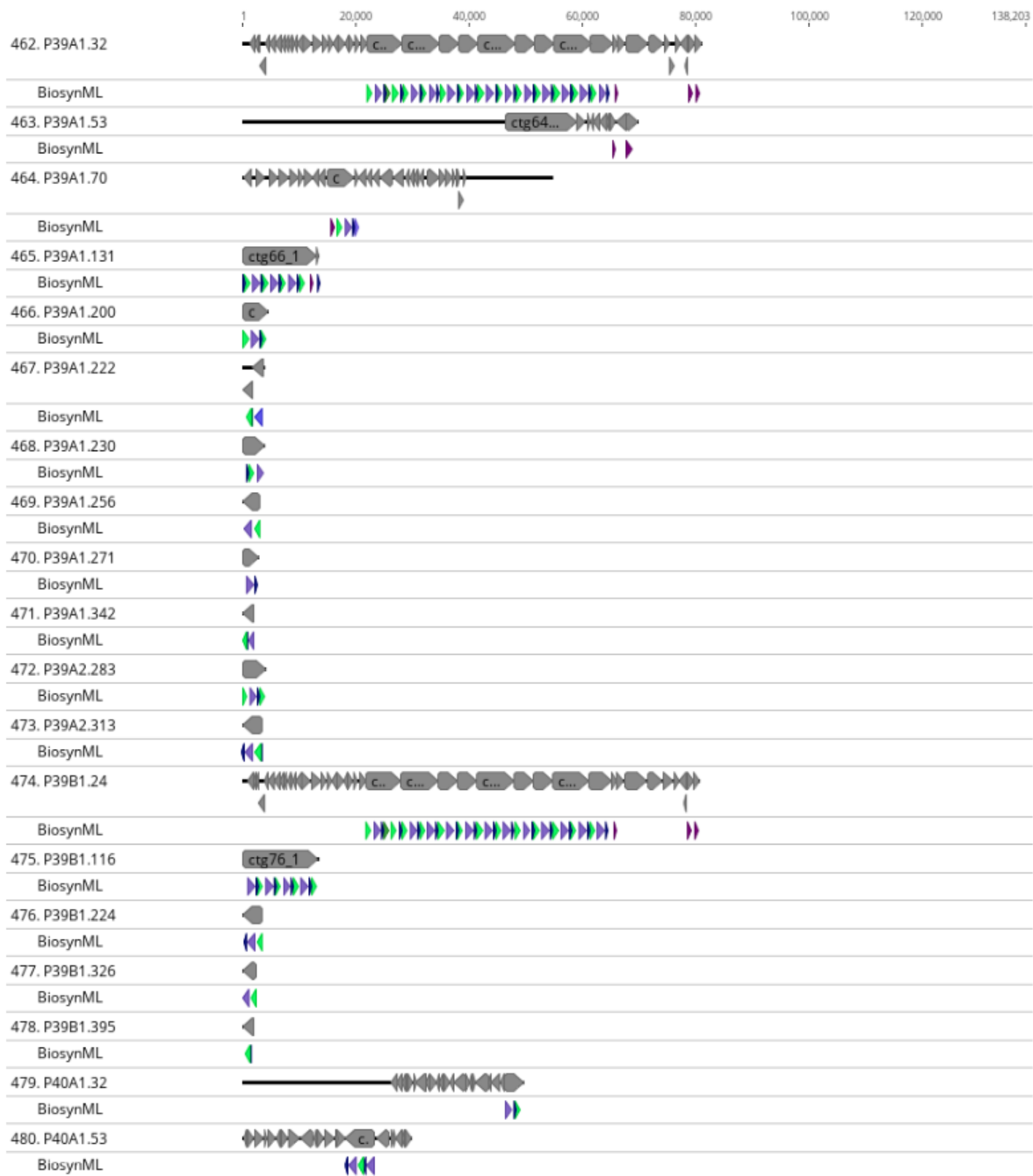

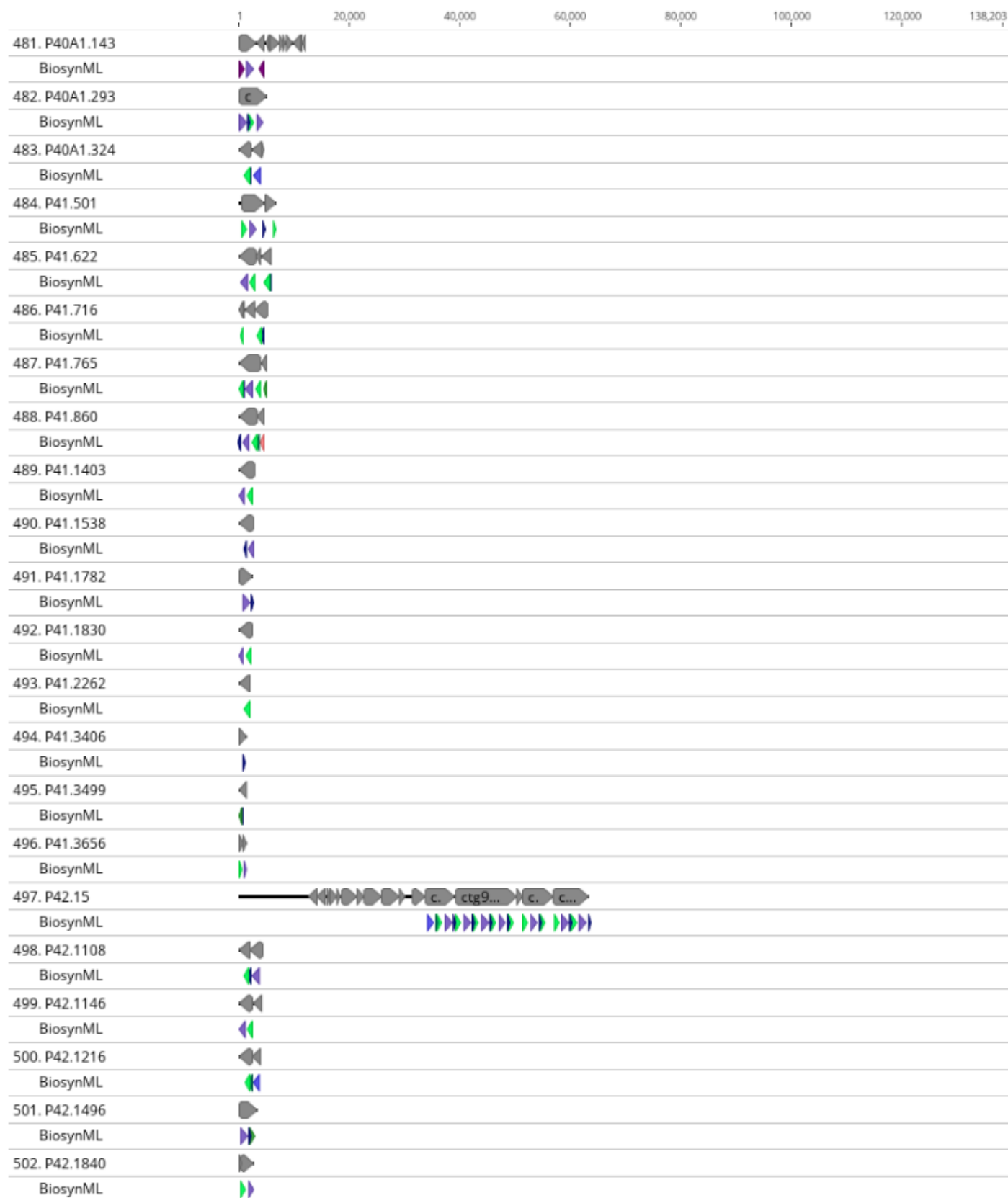

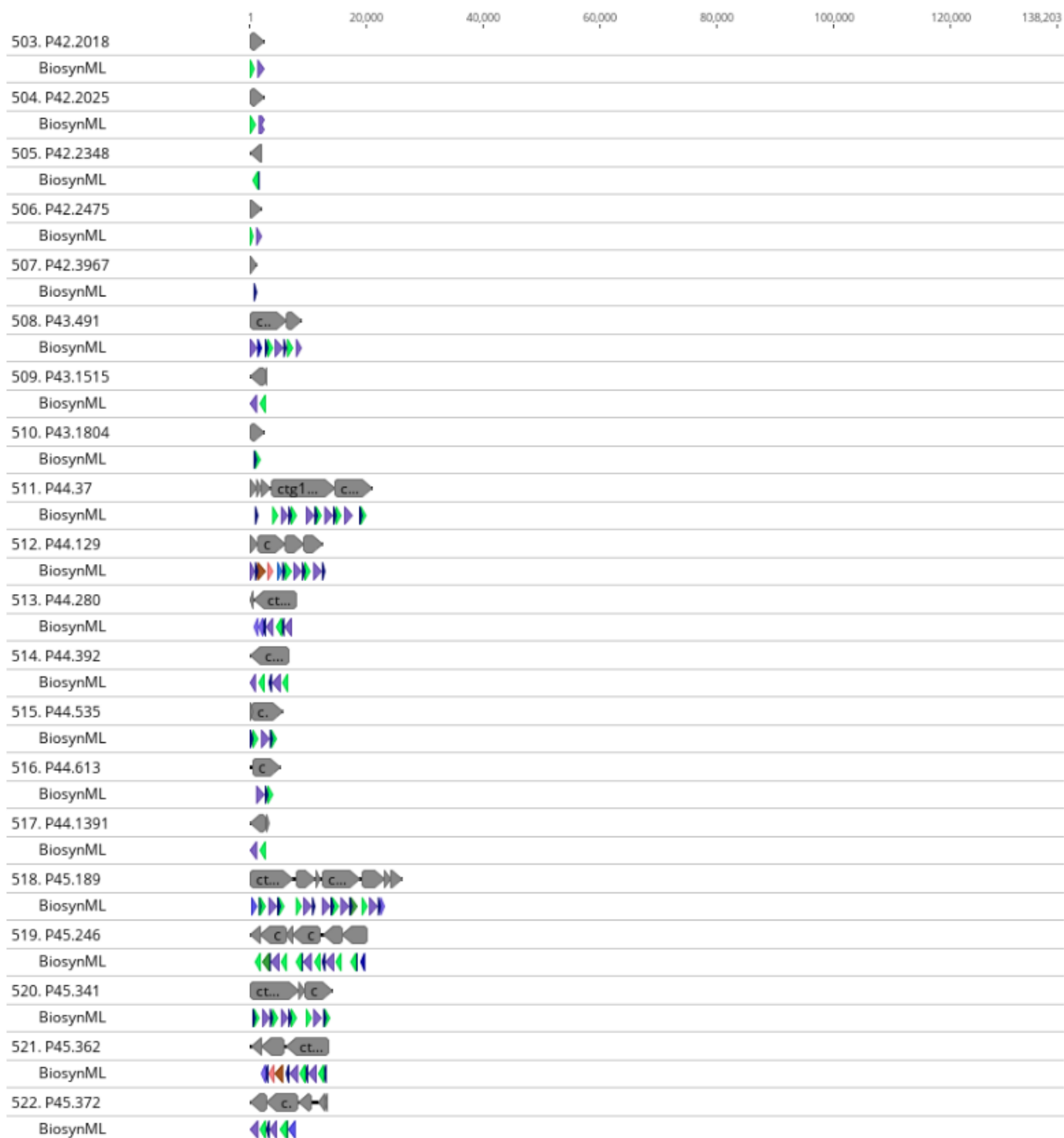

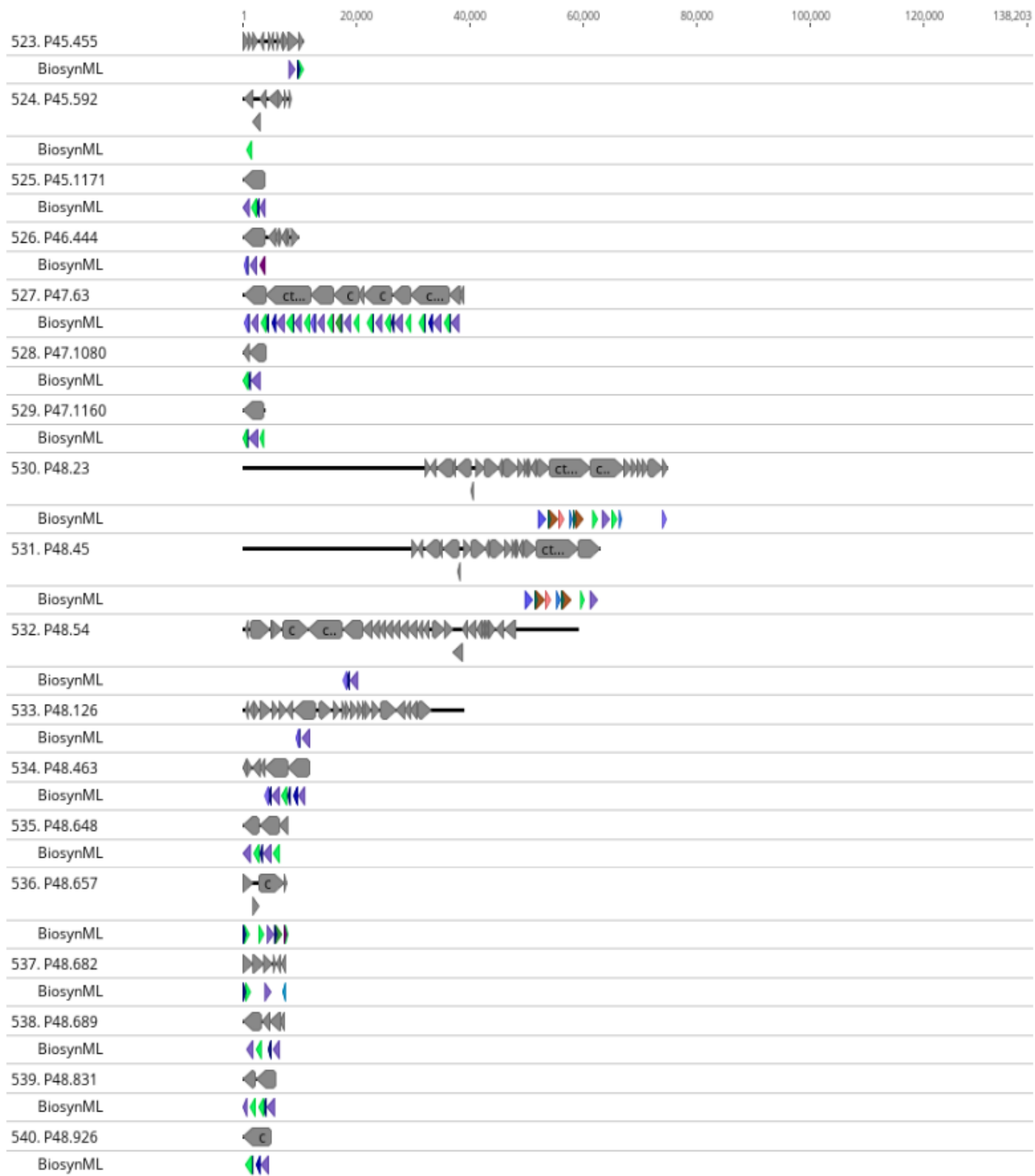

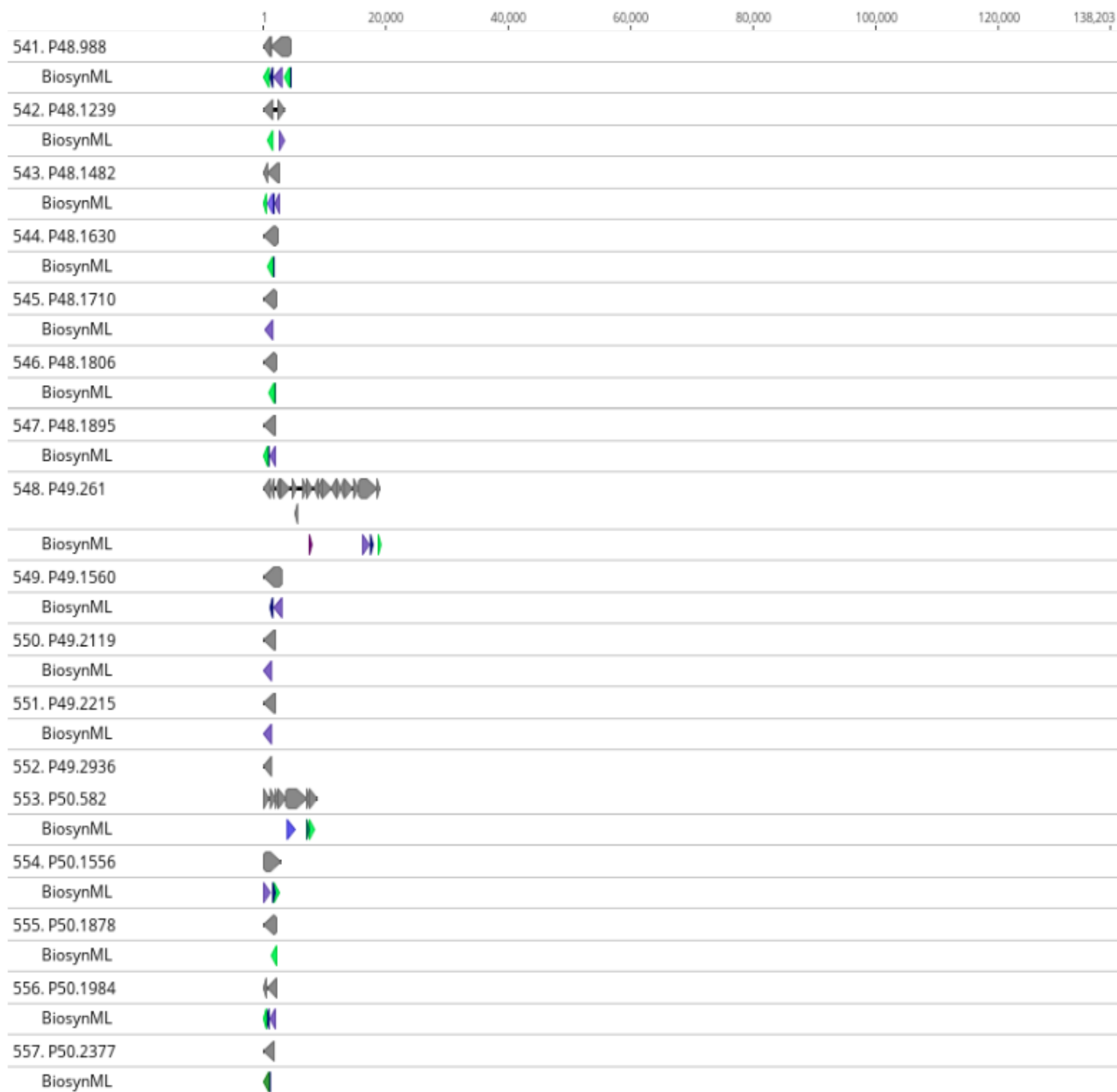

b.

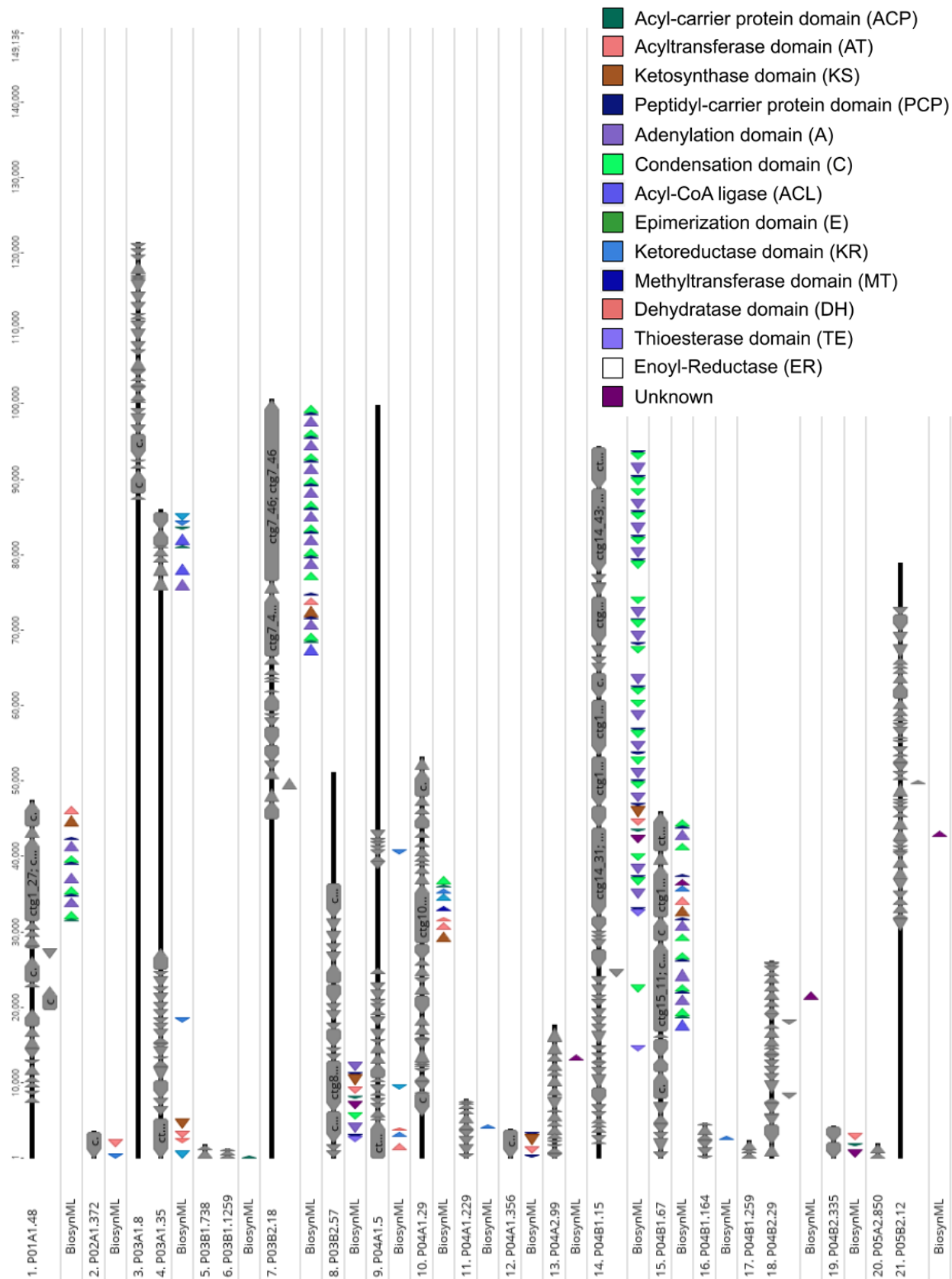

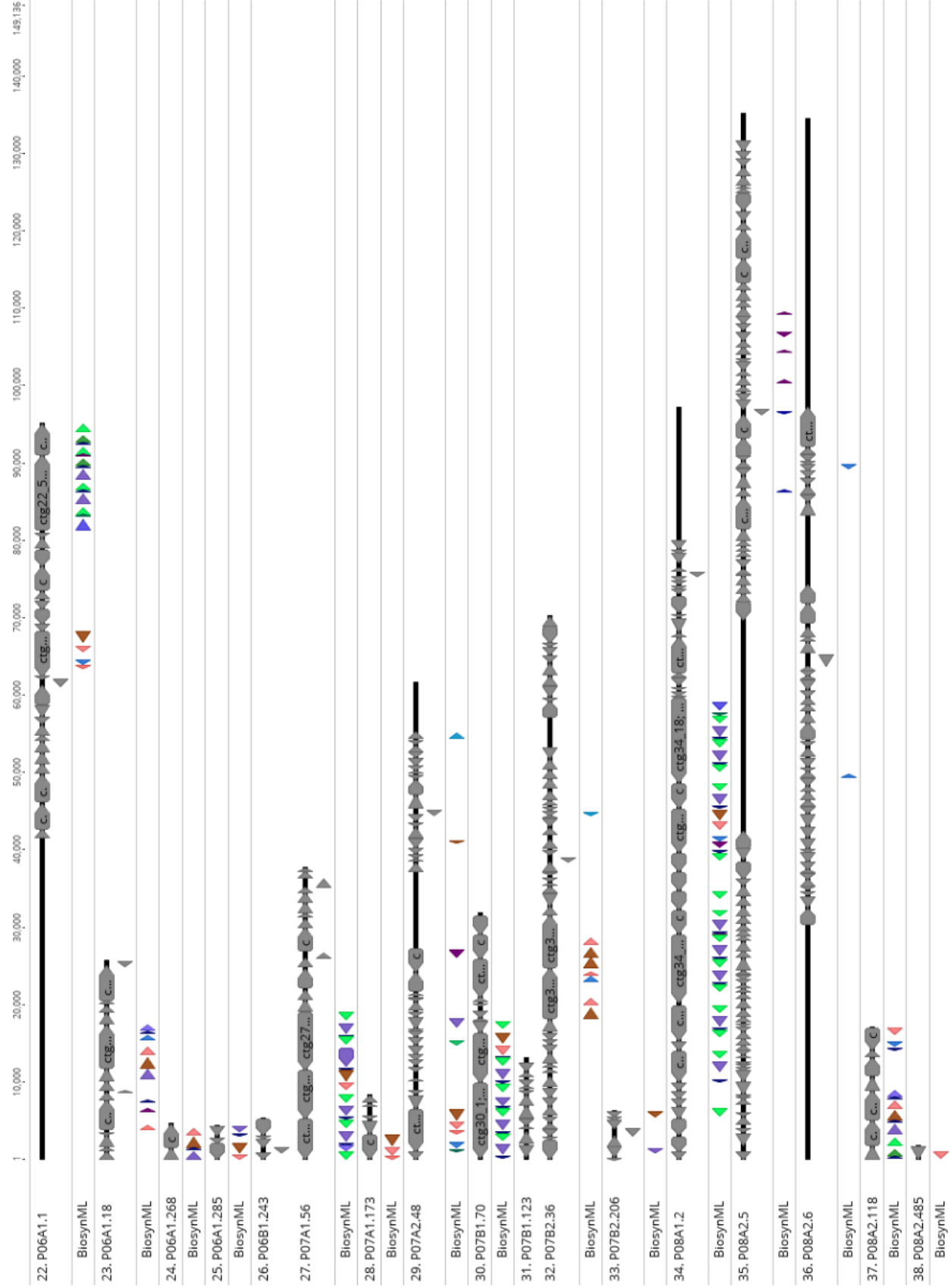

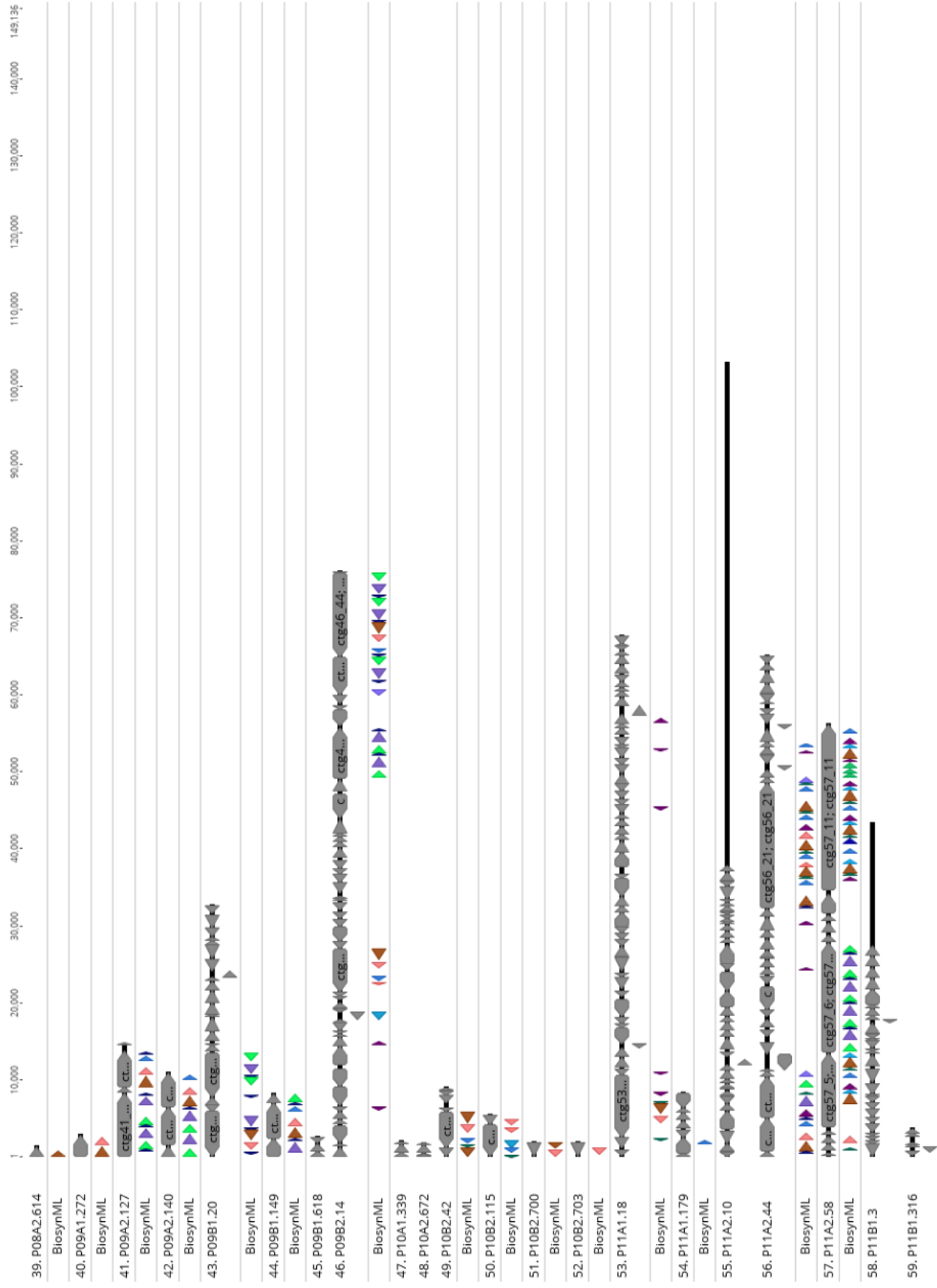

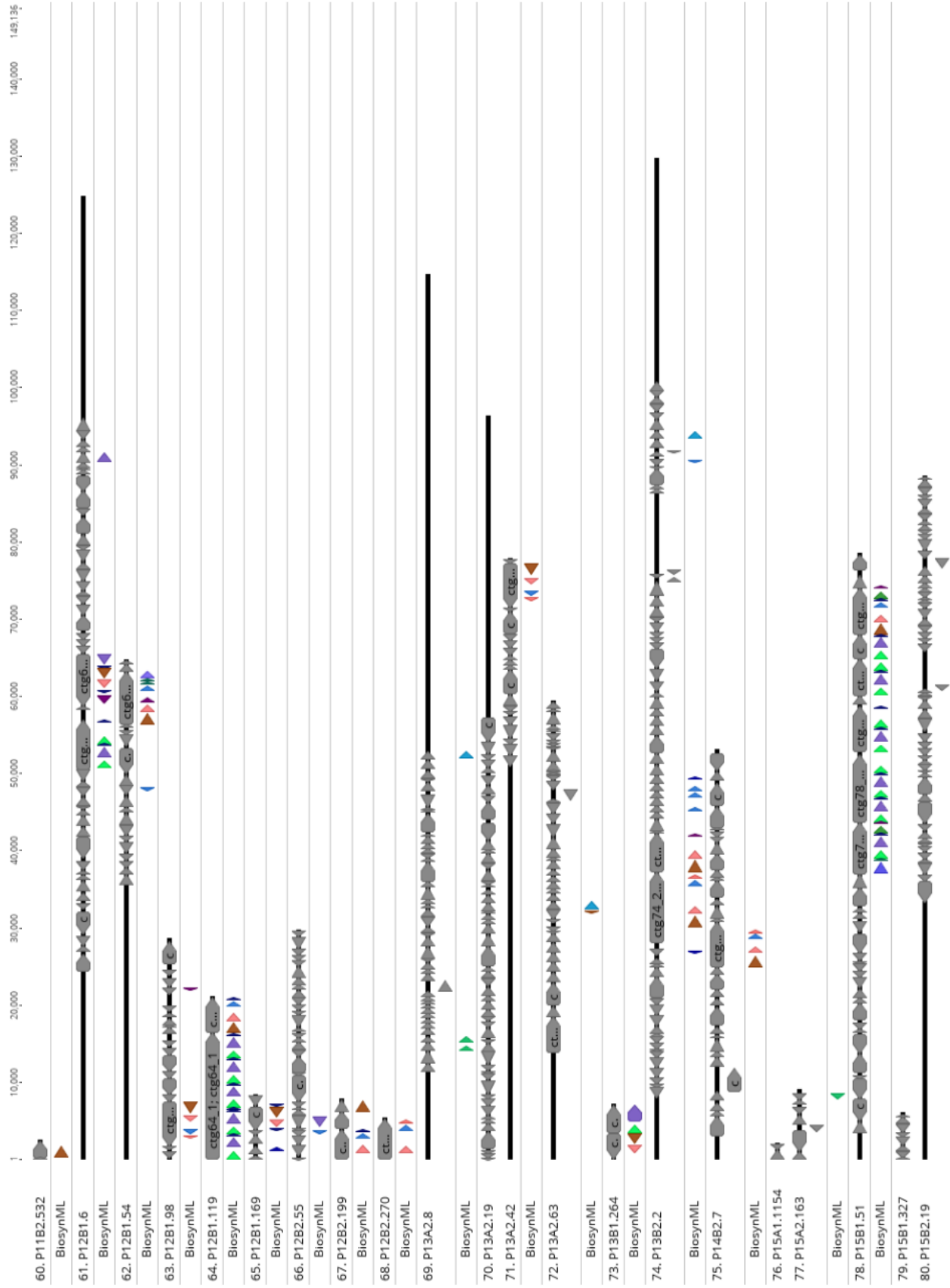

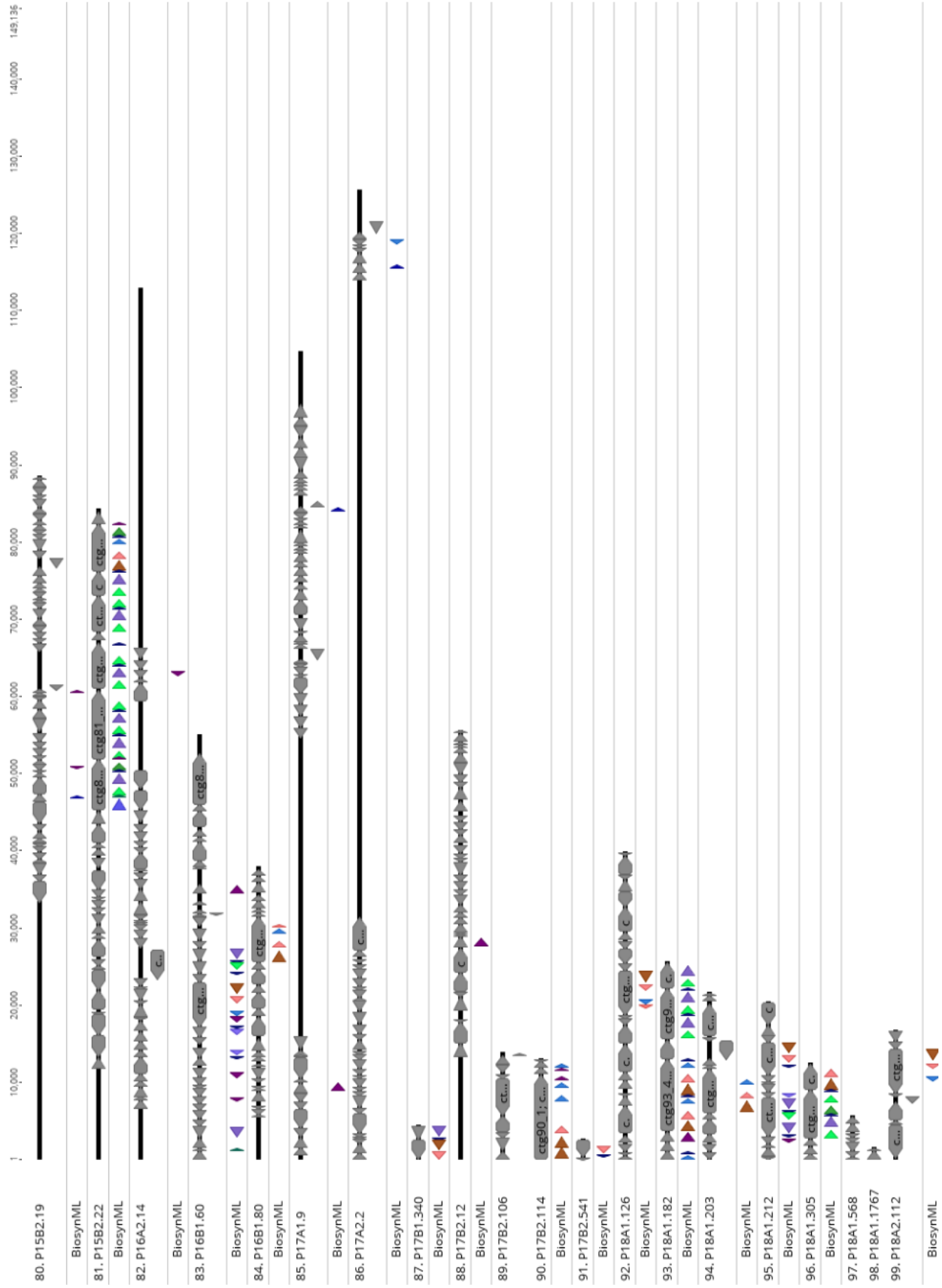

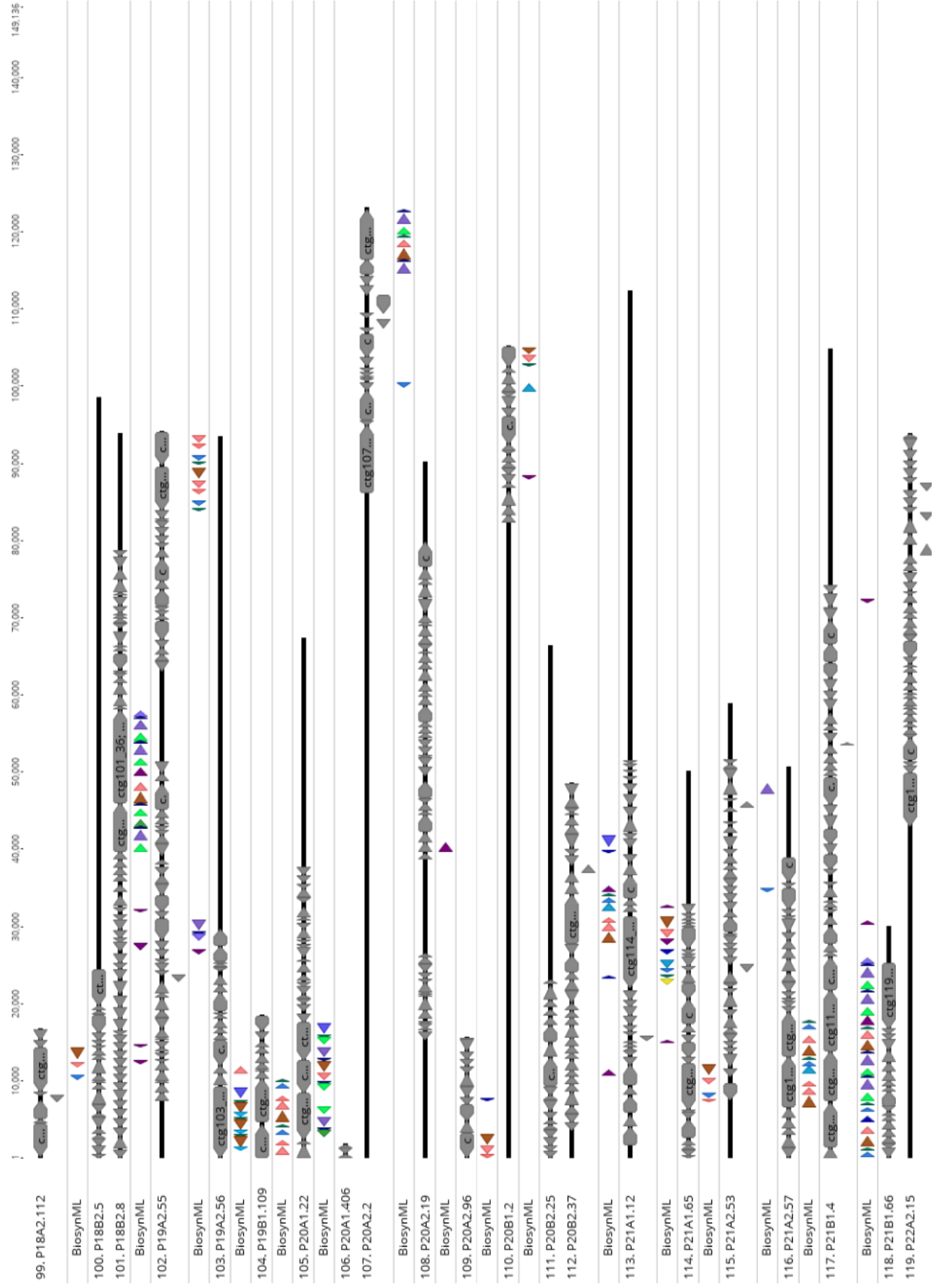

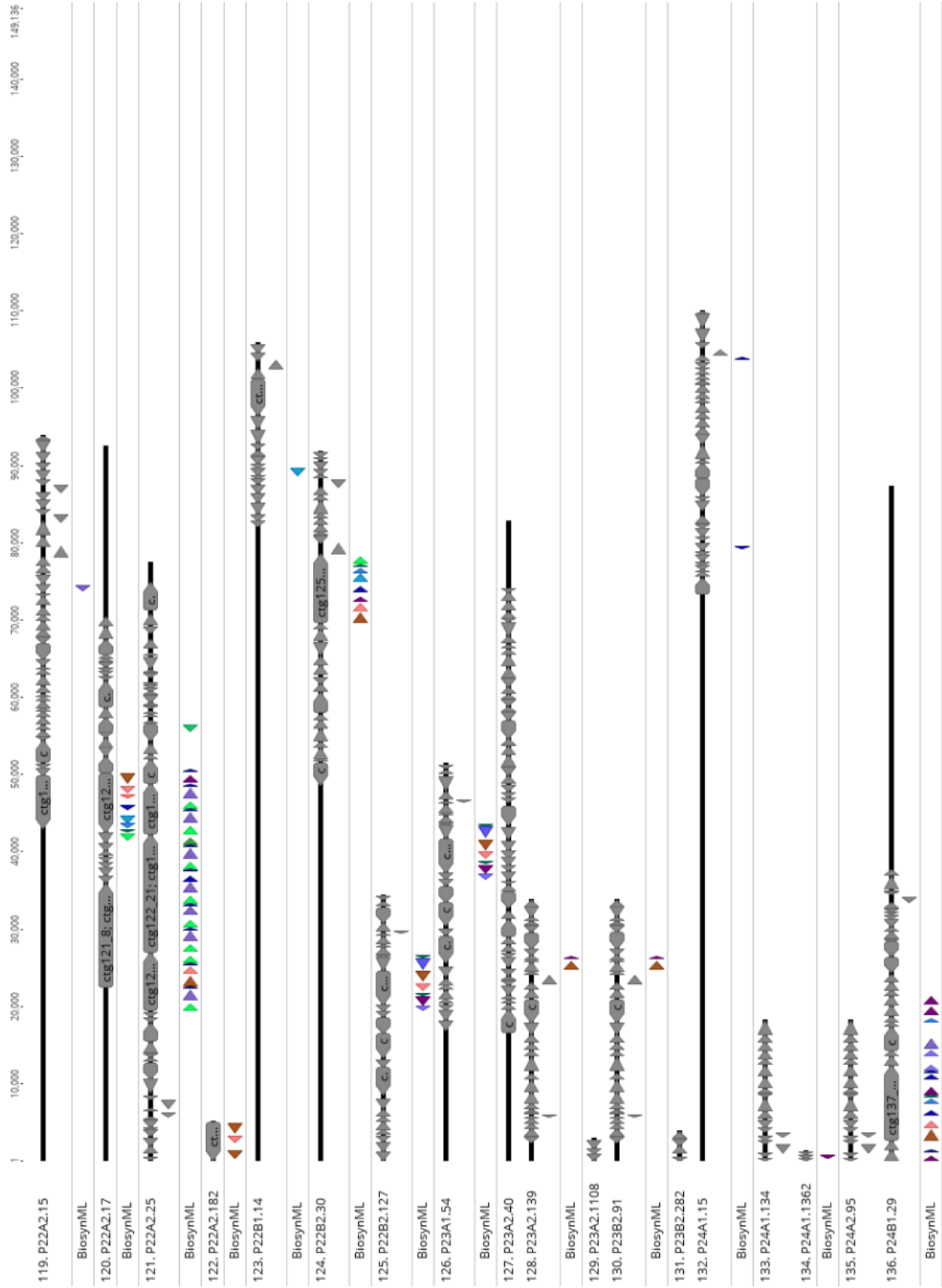

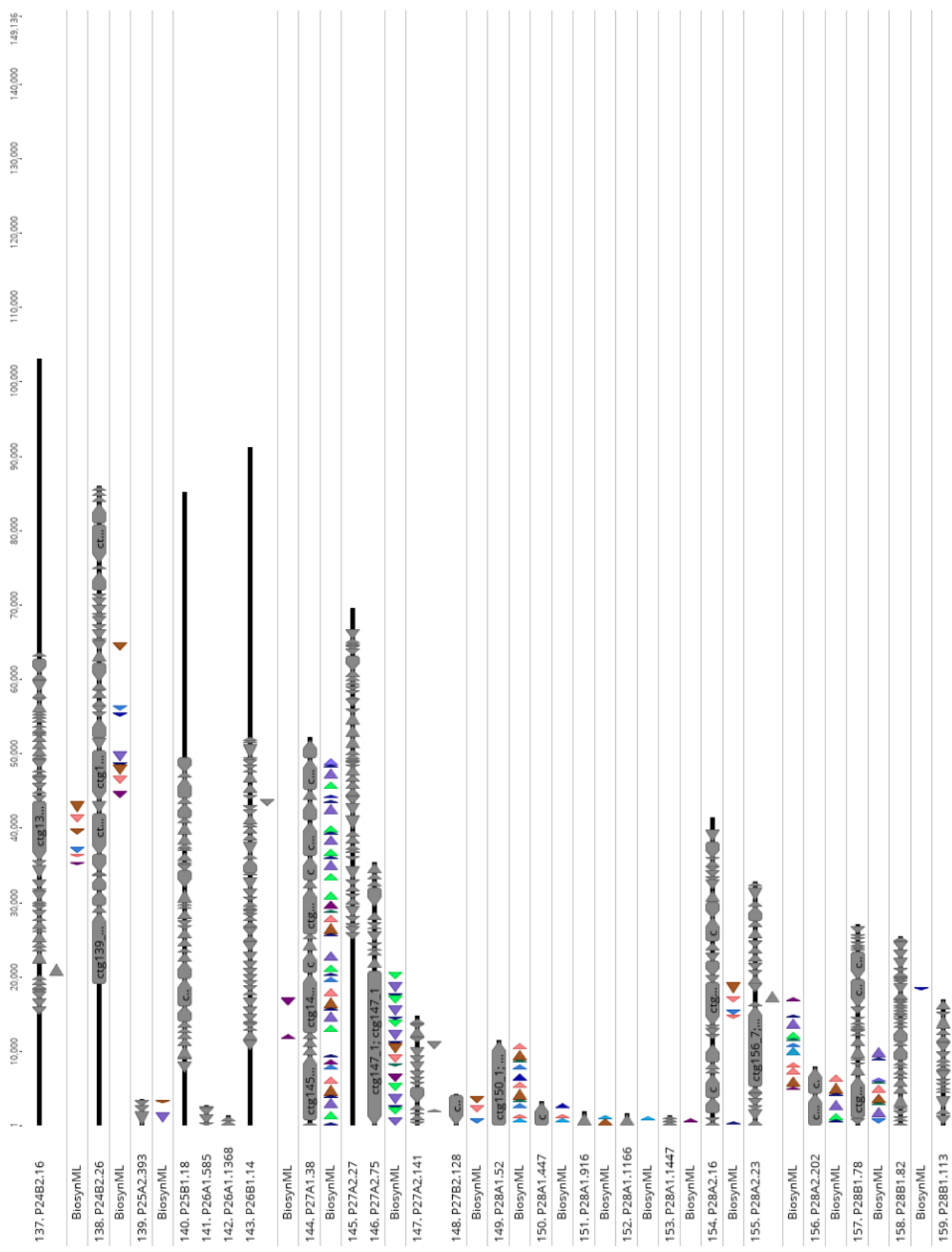

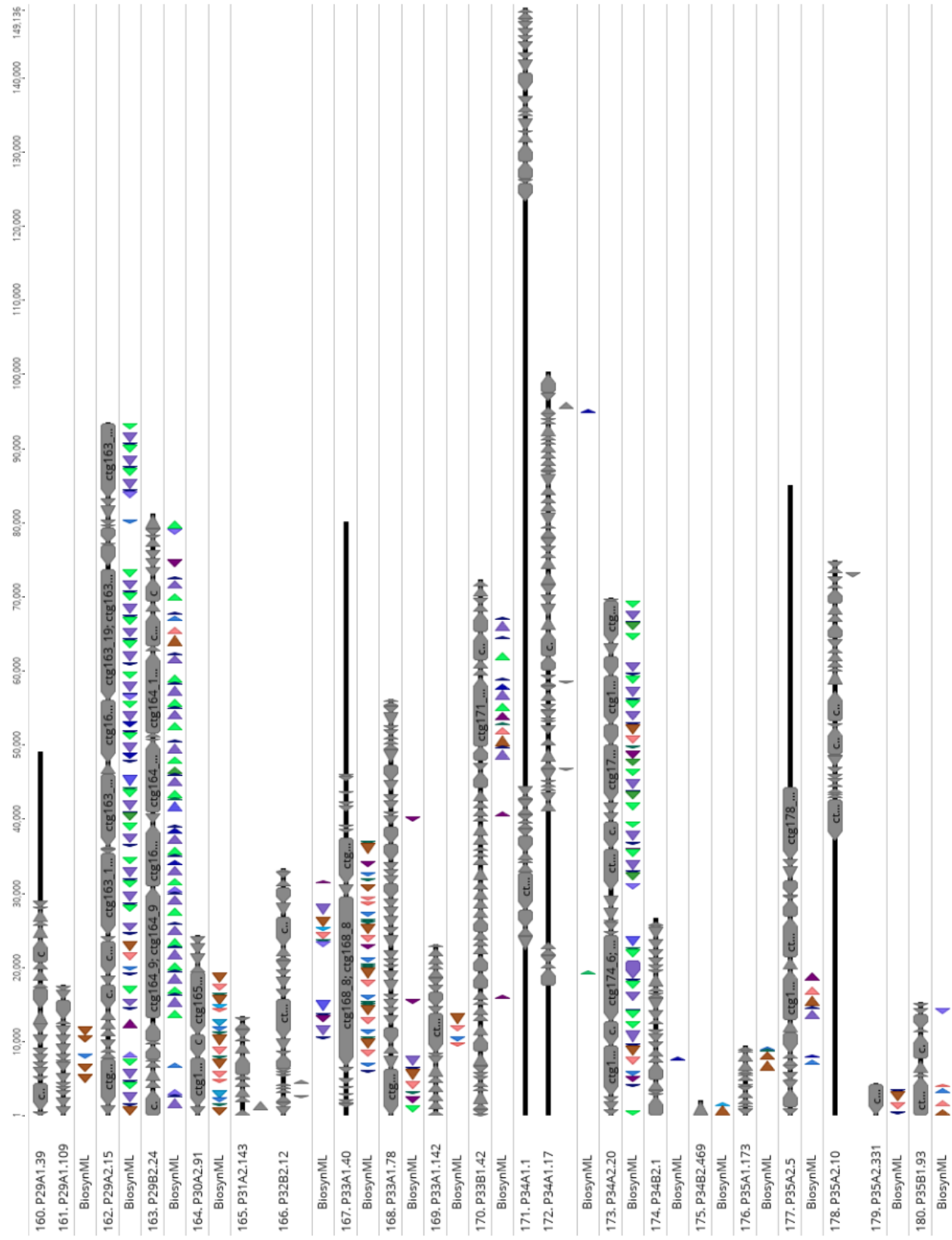

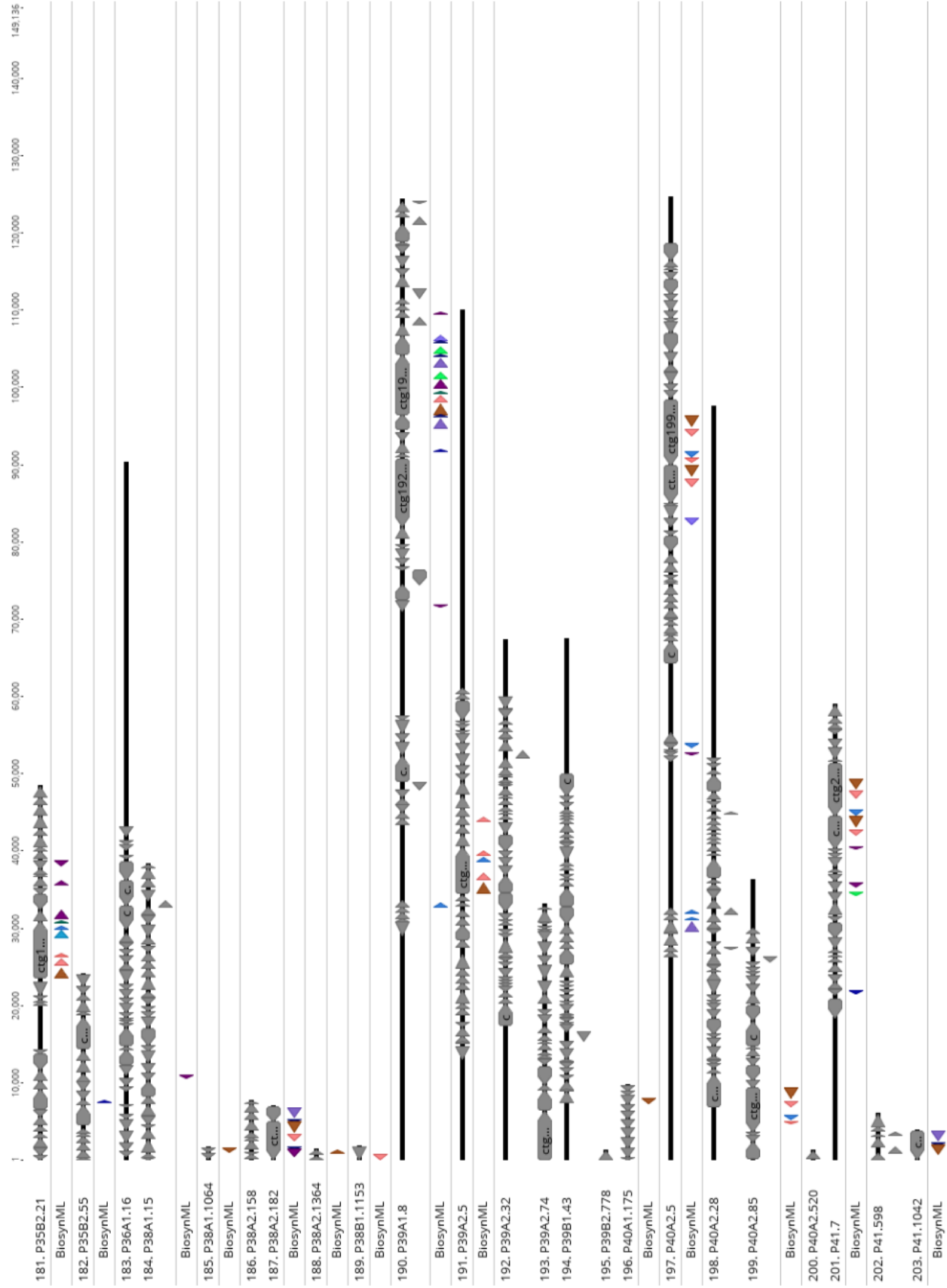

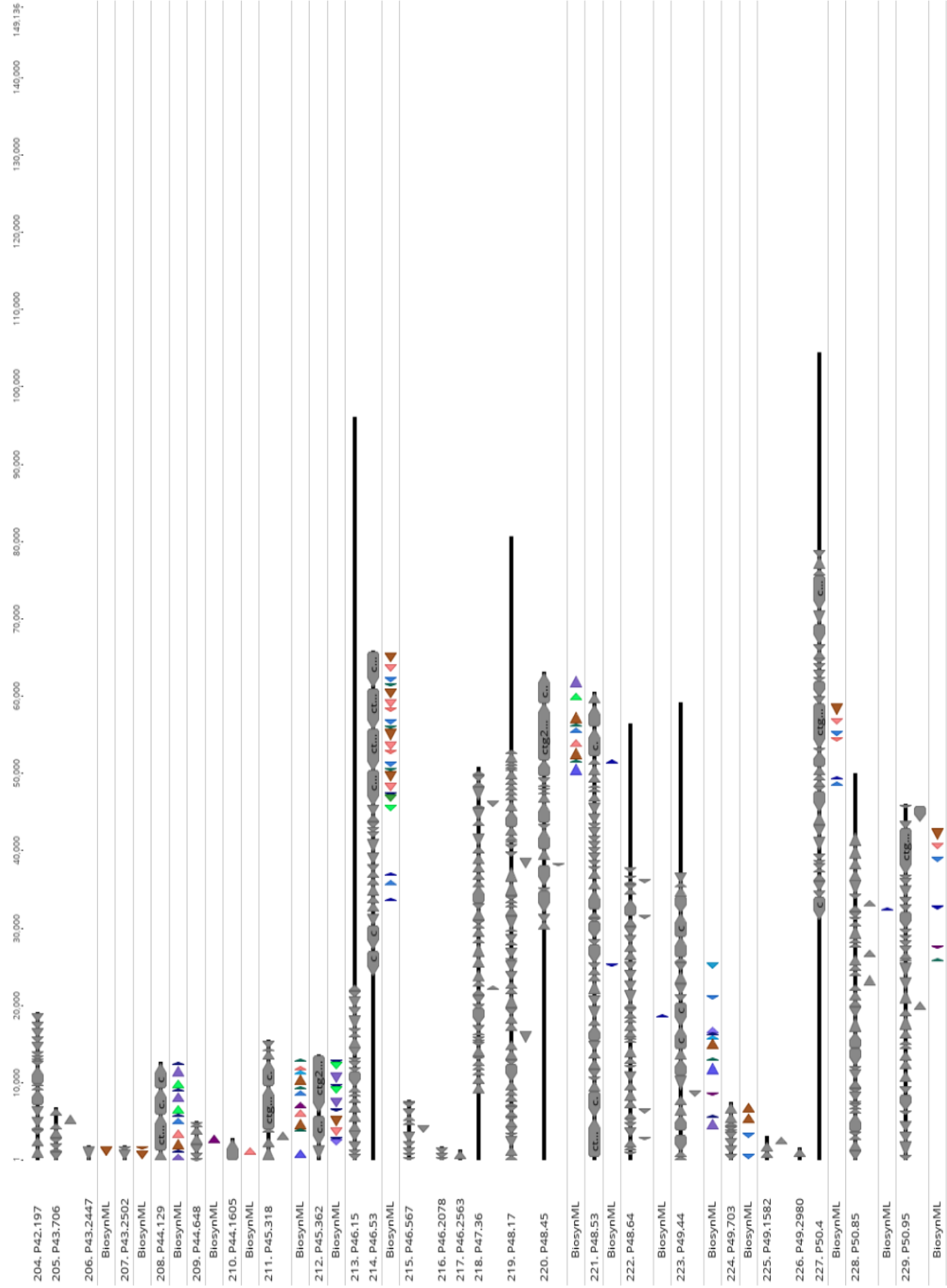

c.

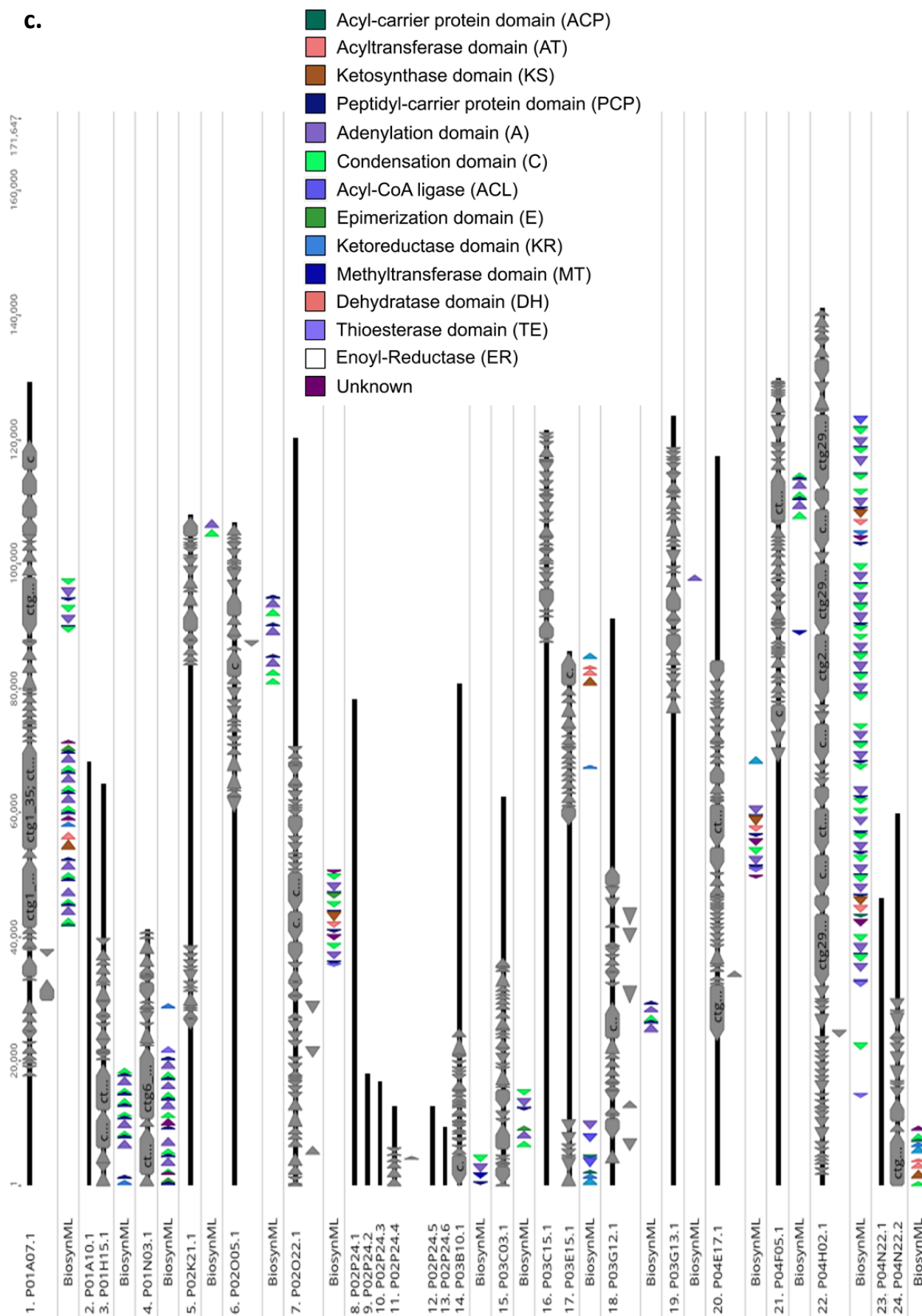

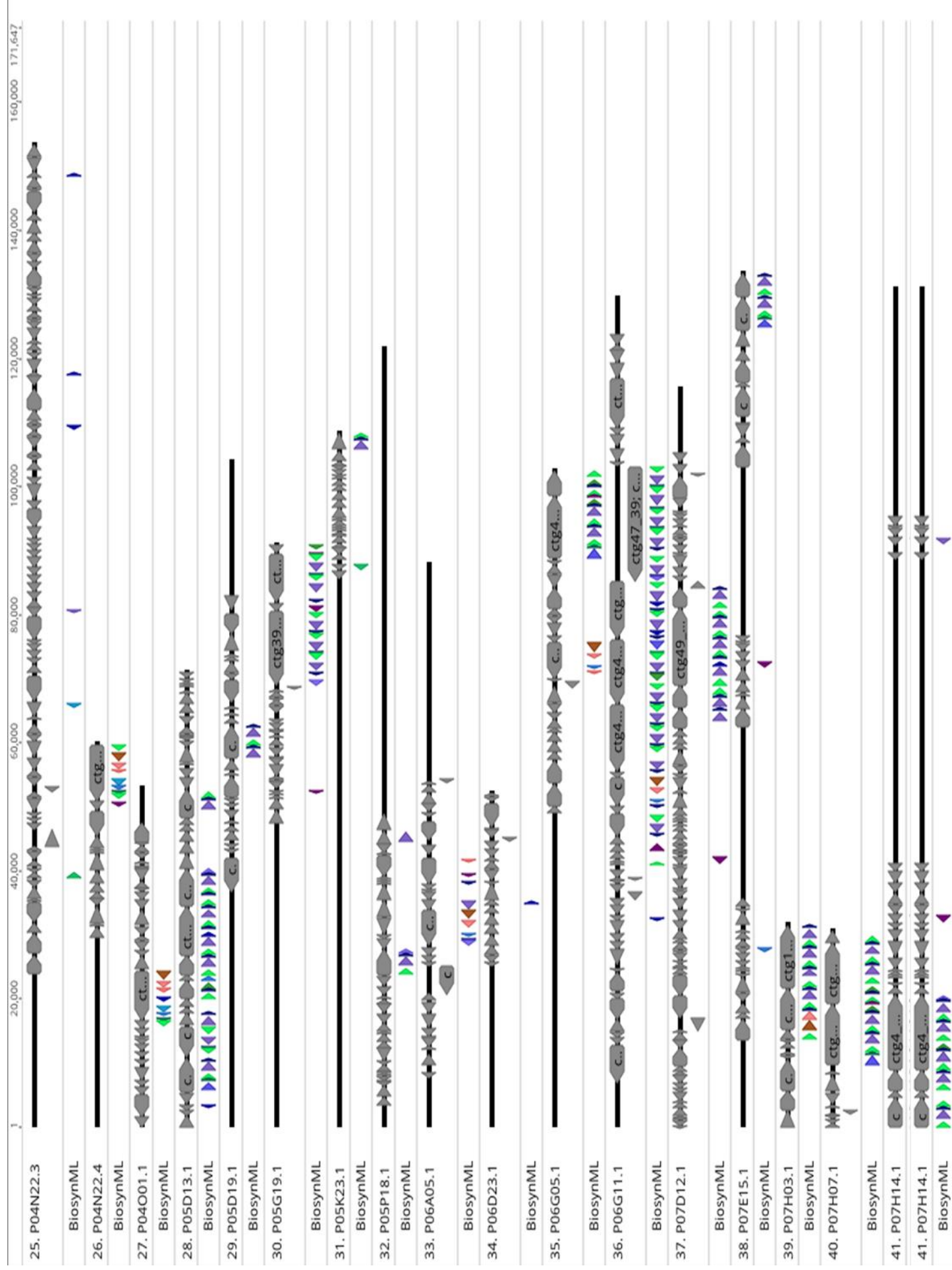

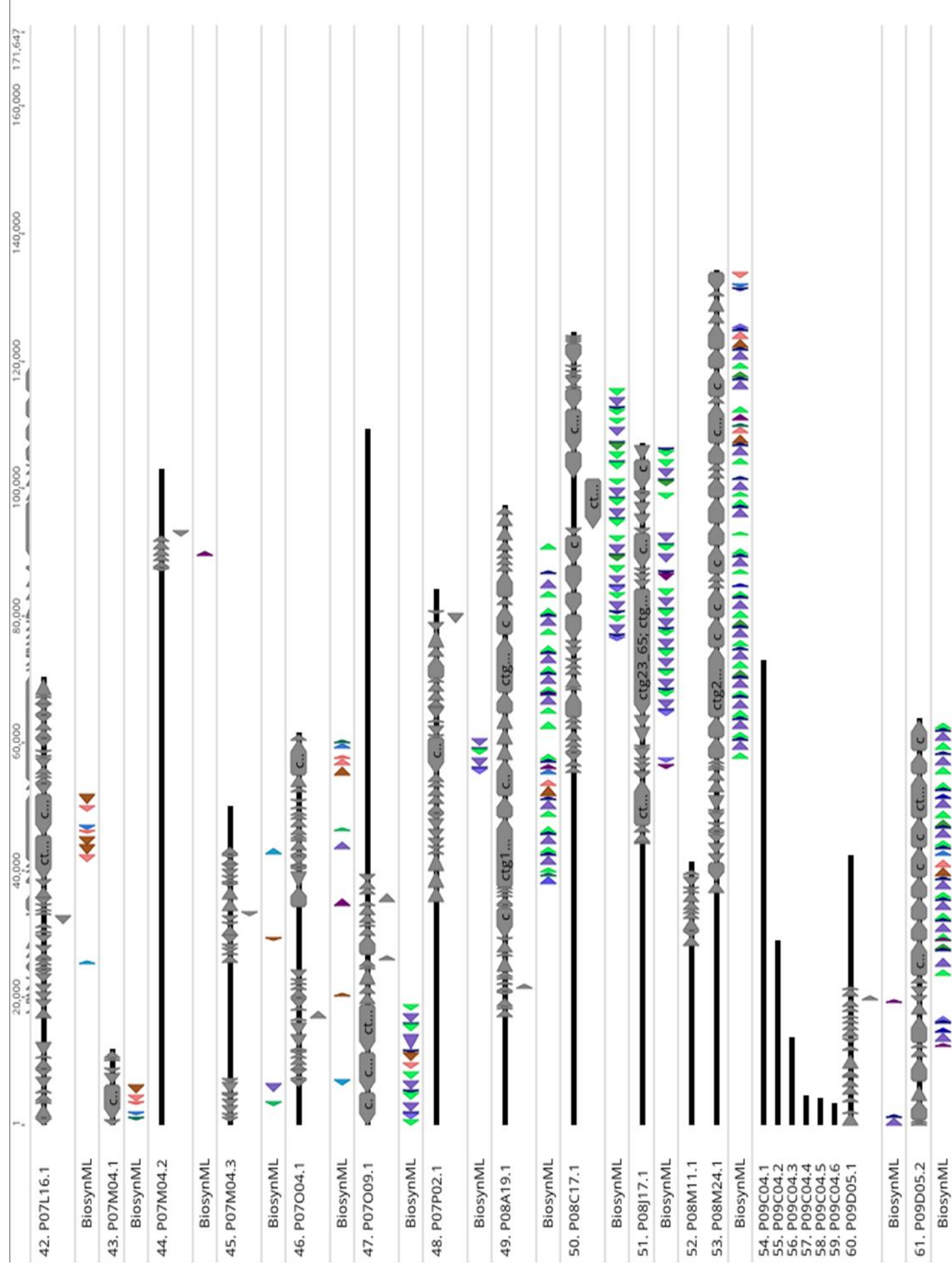

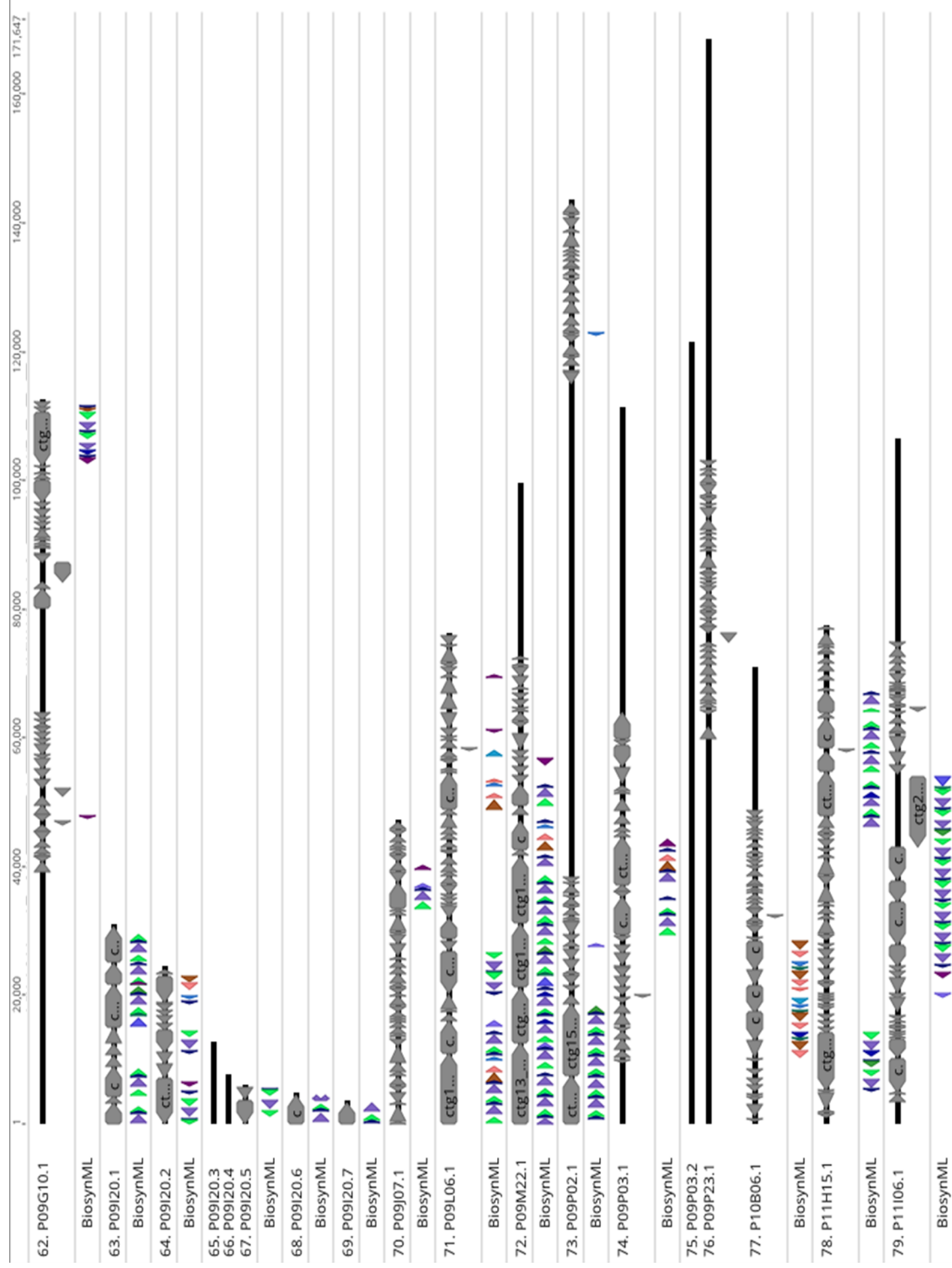

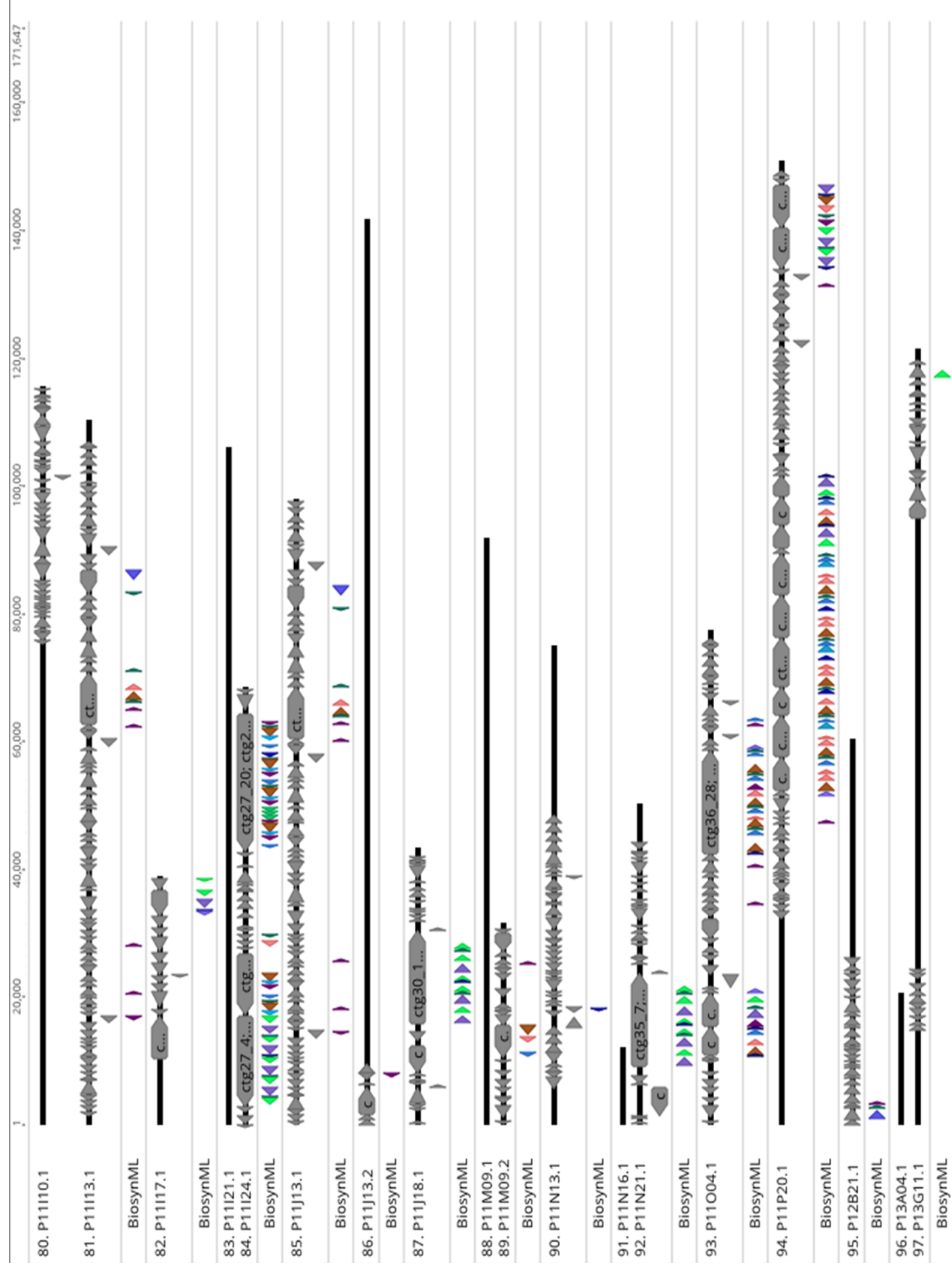

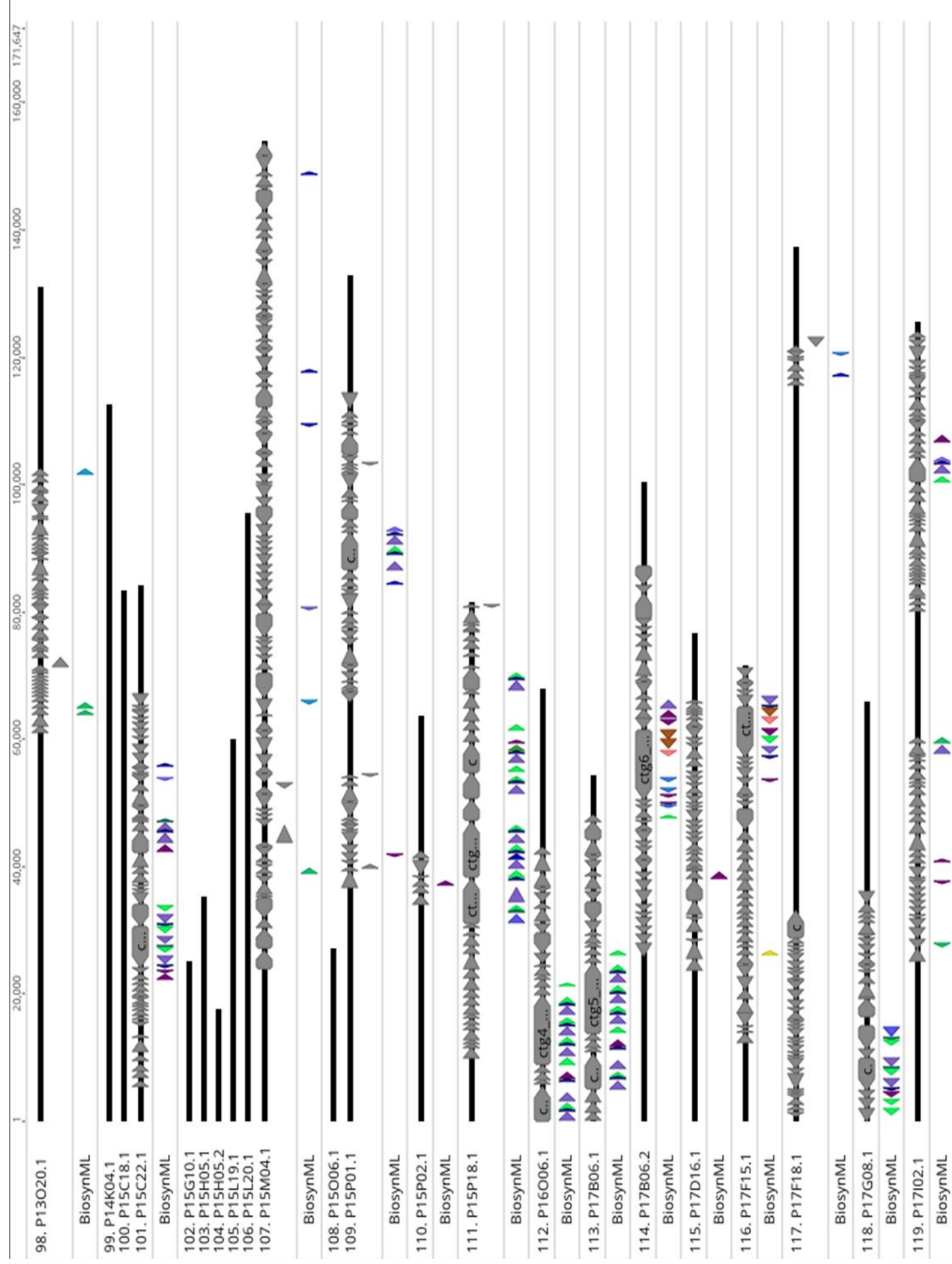

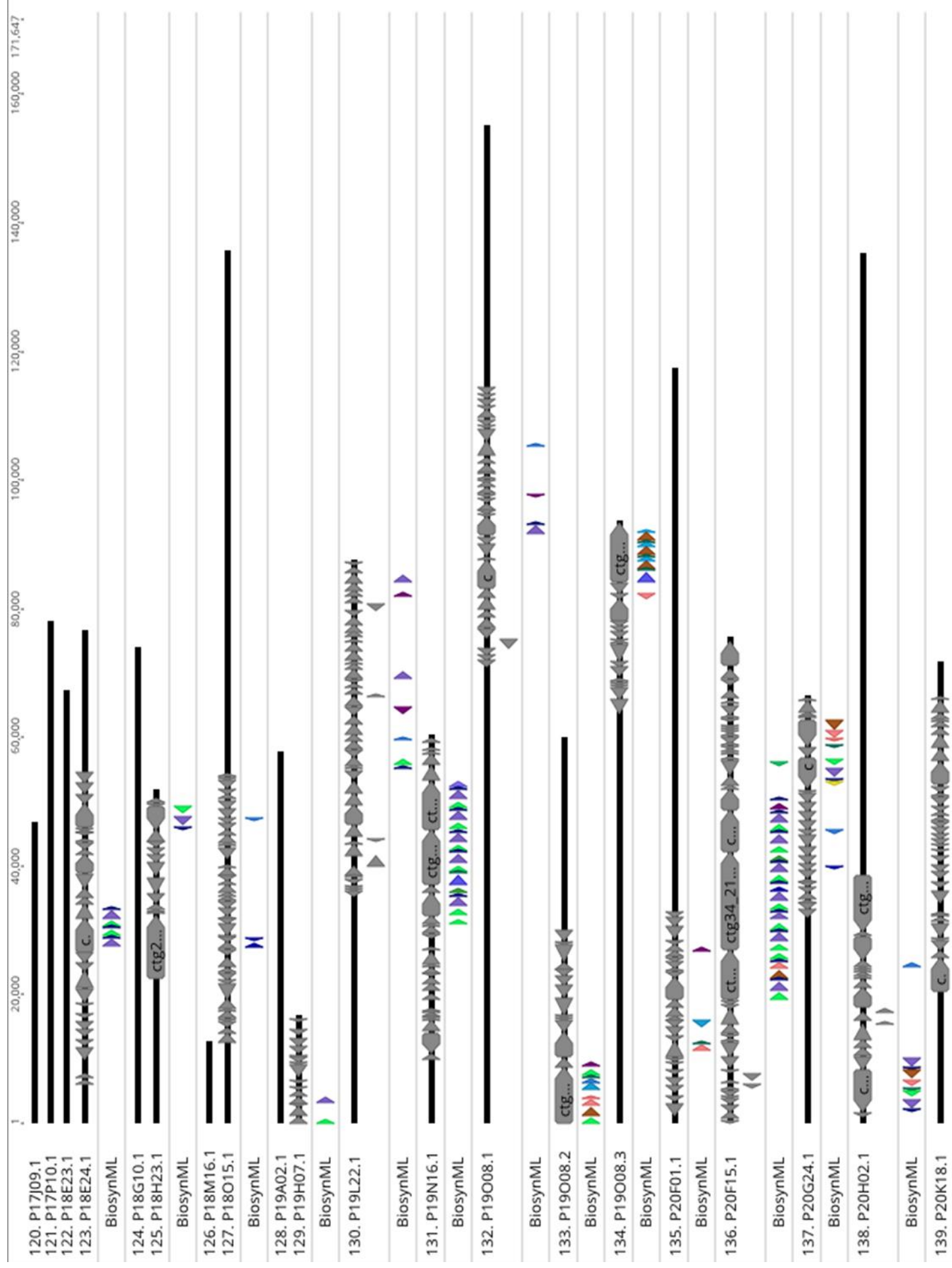

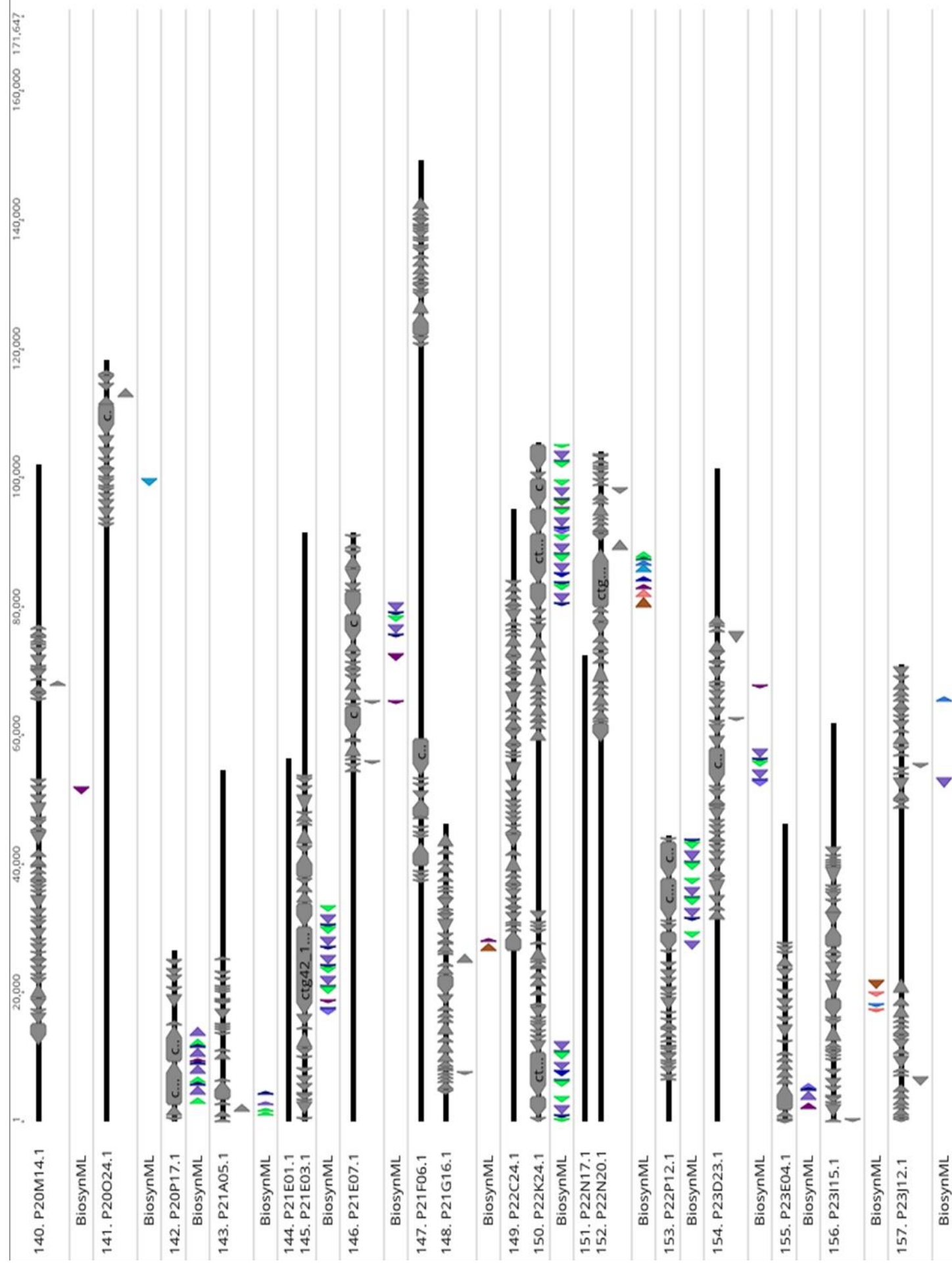

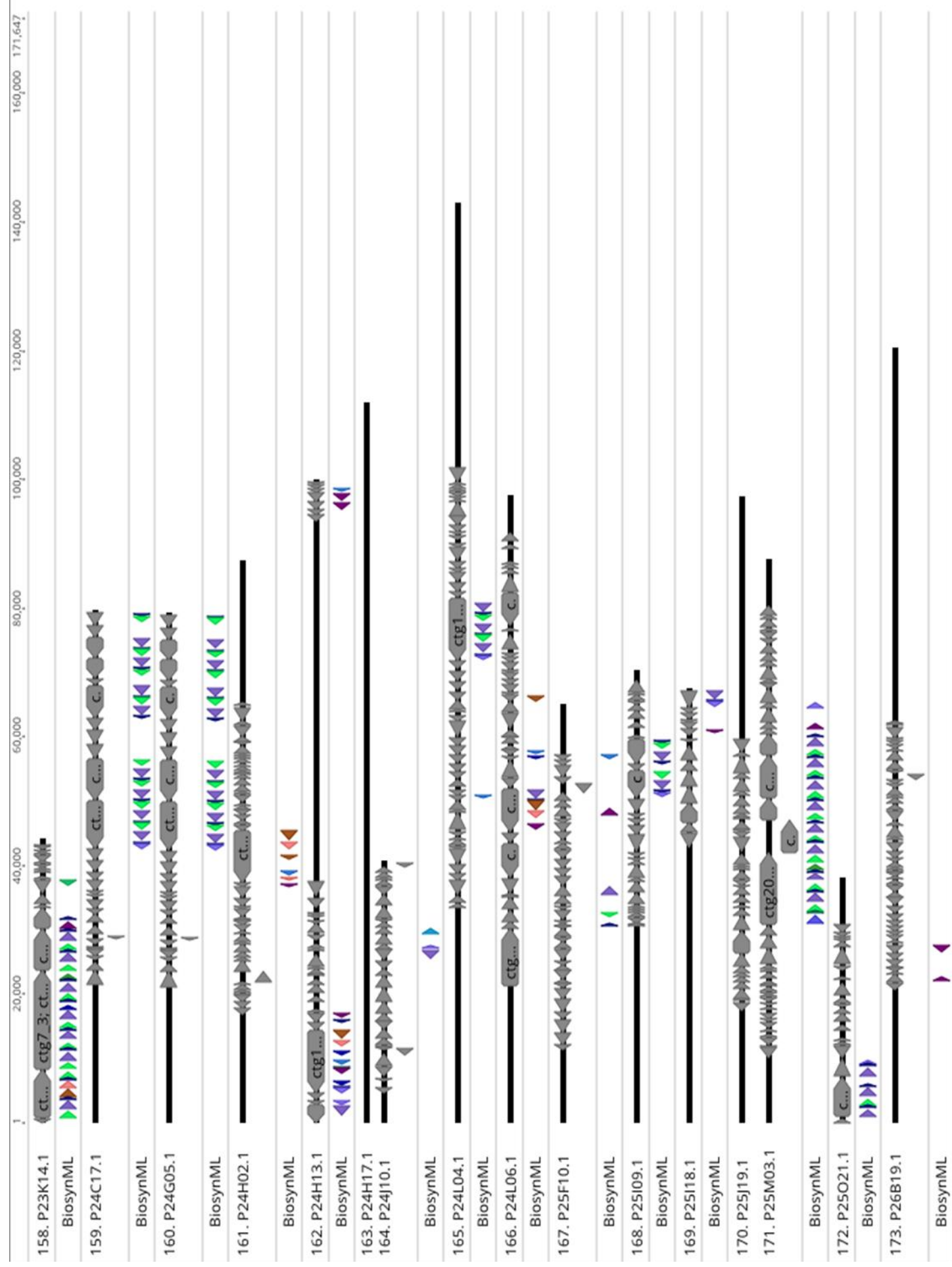

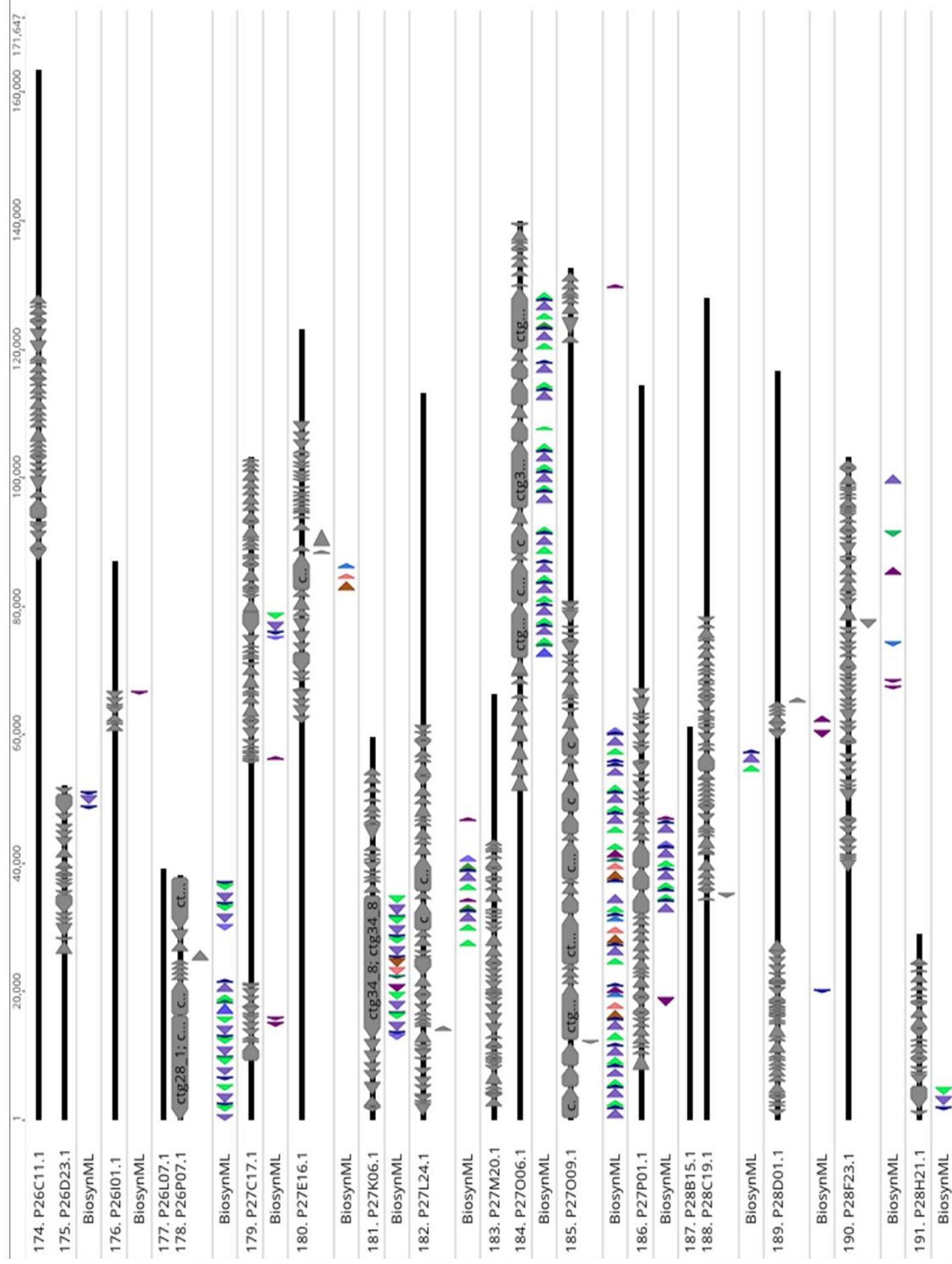

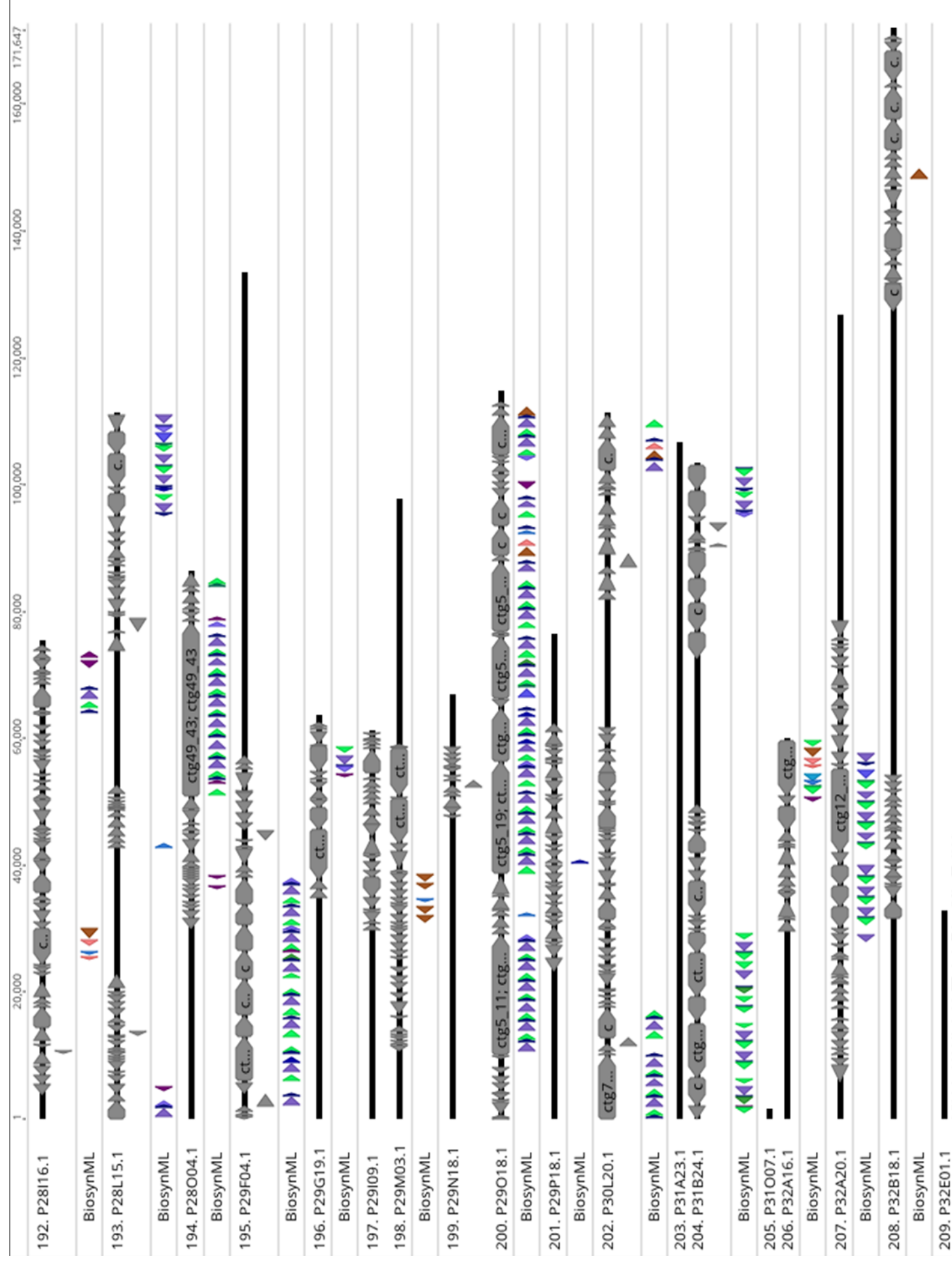

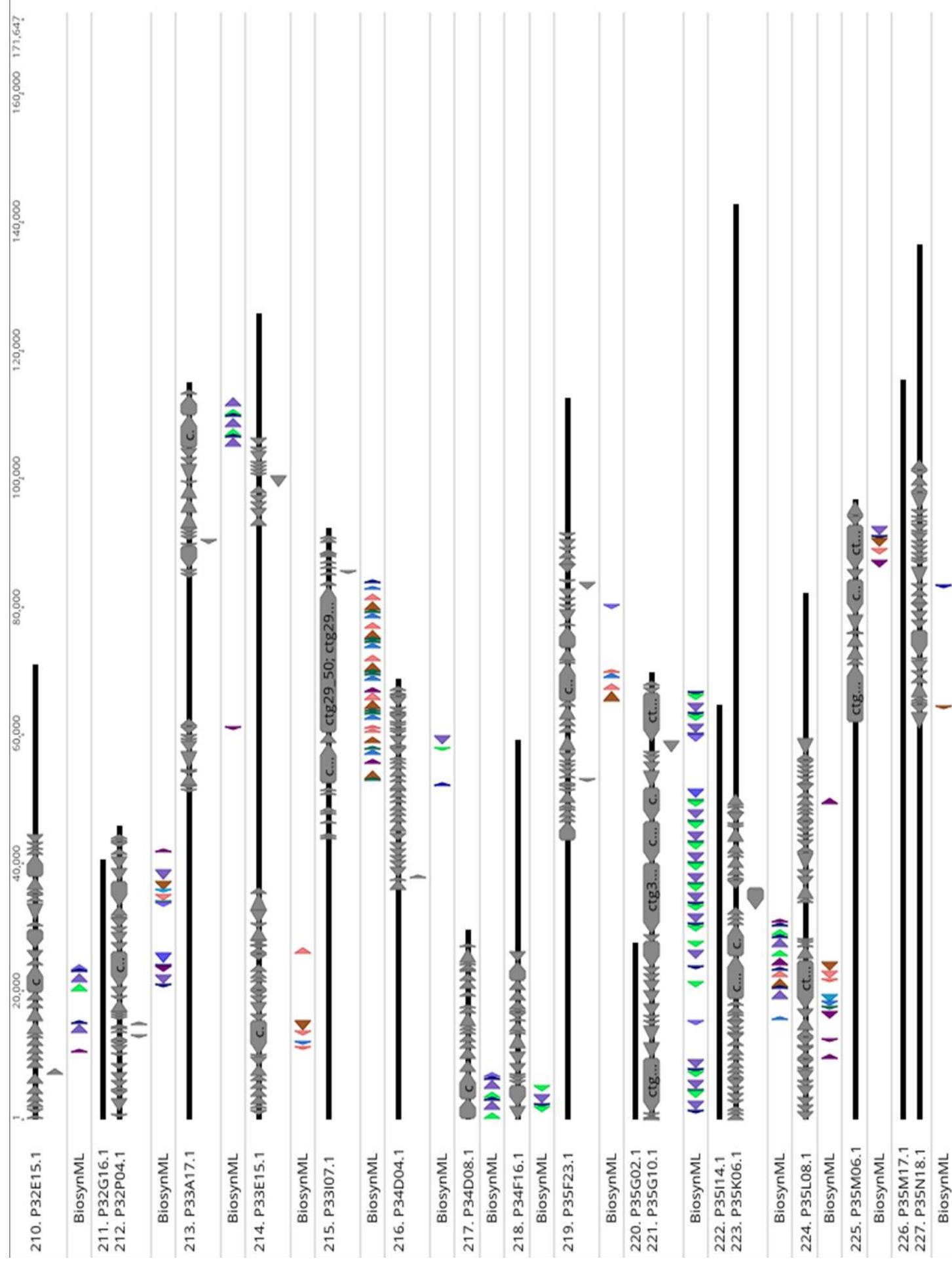

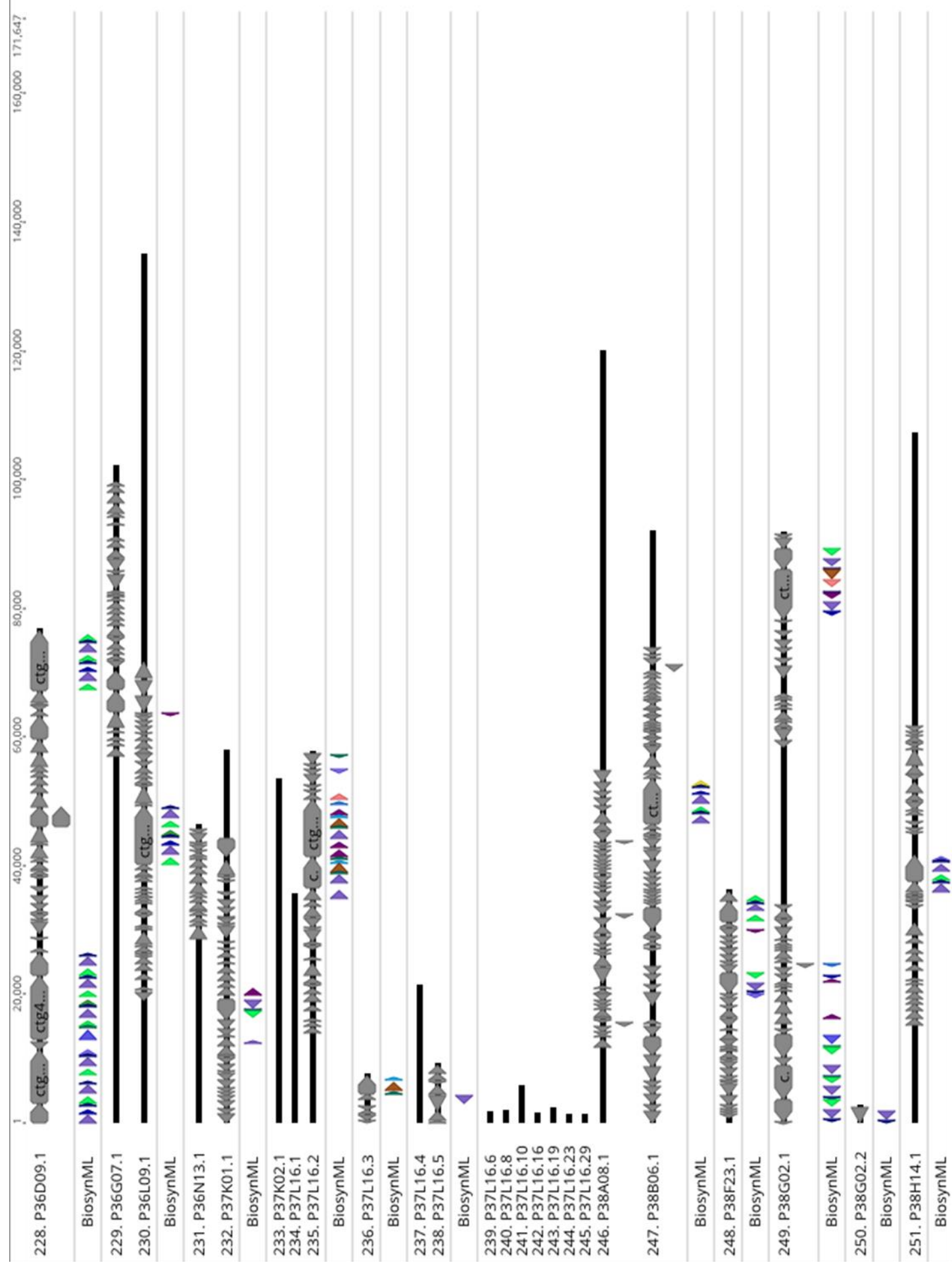

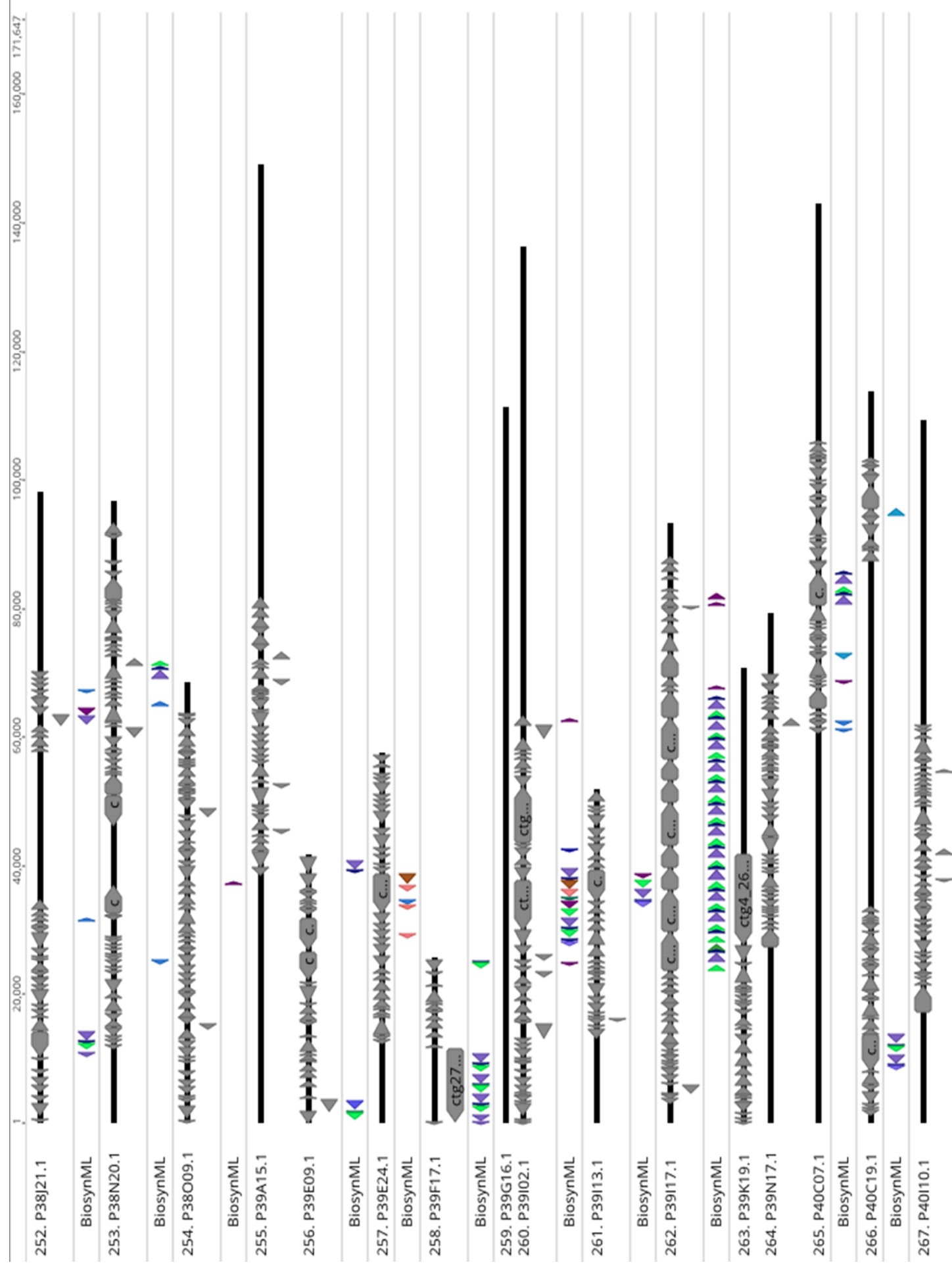

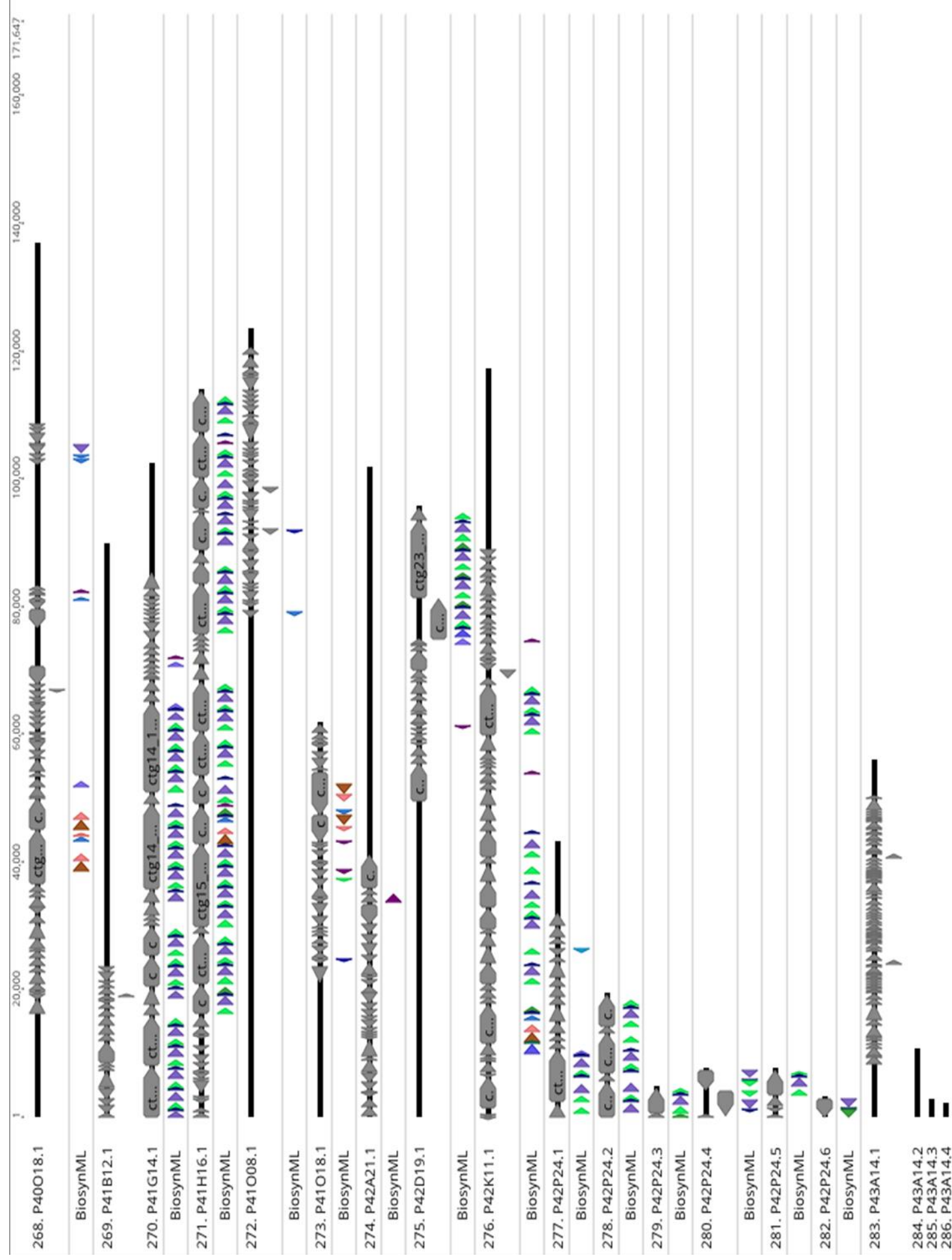

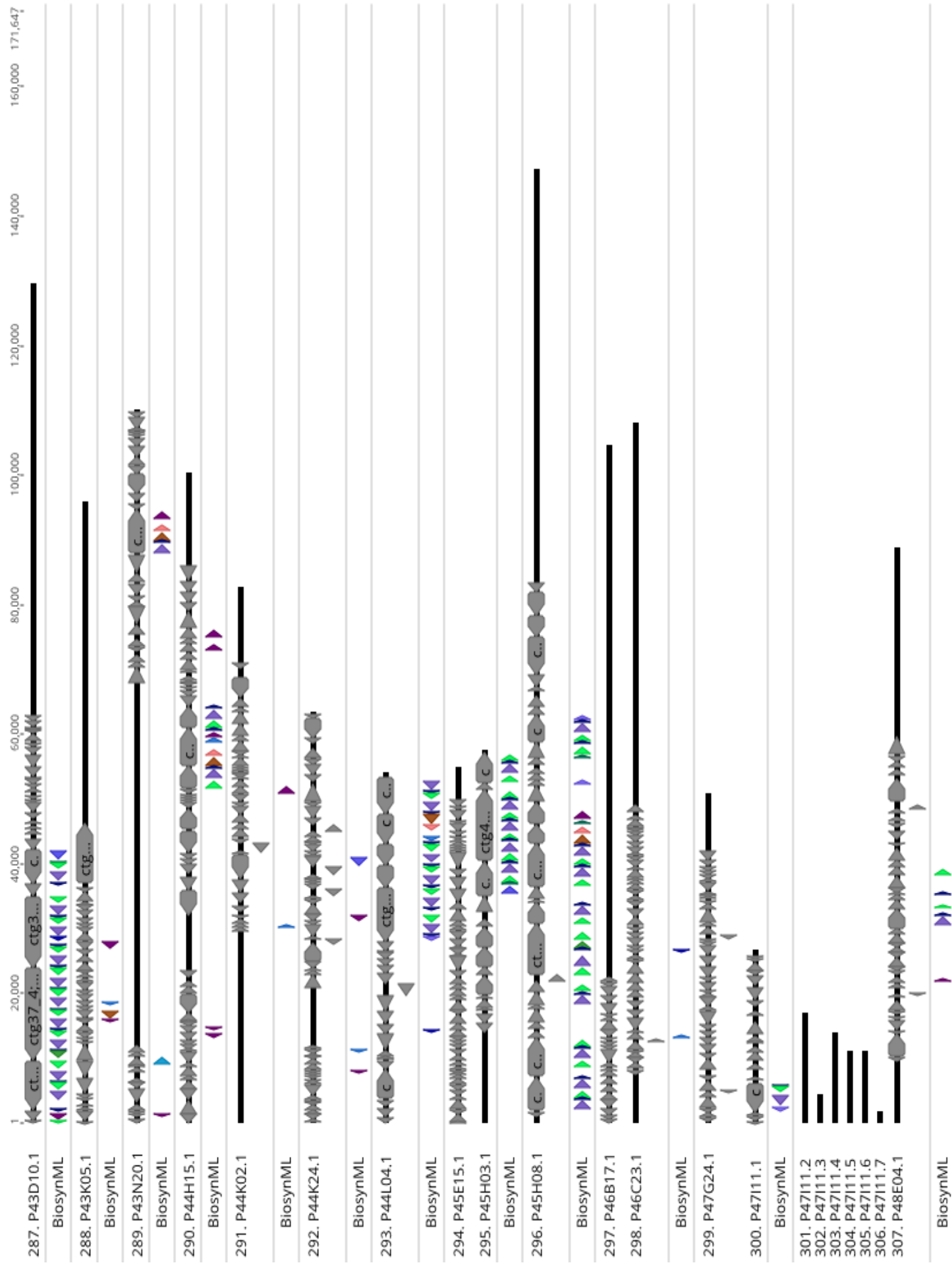

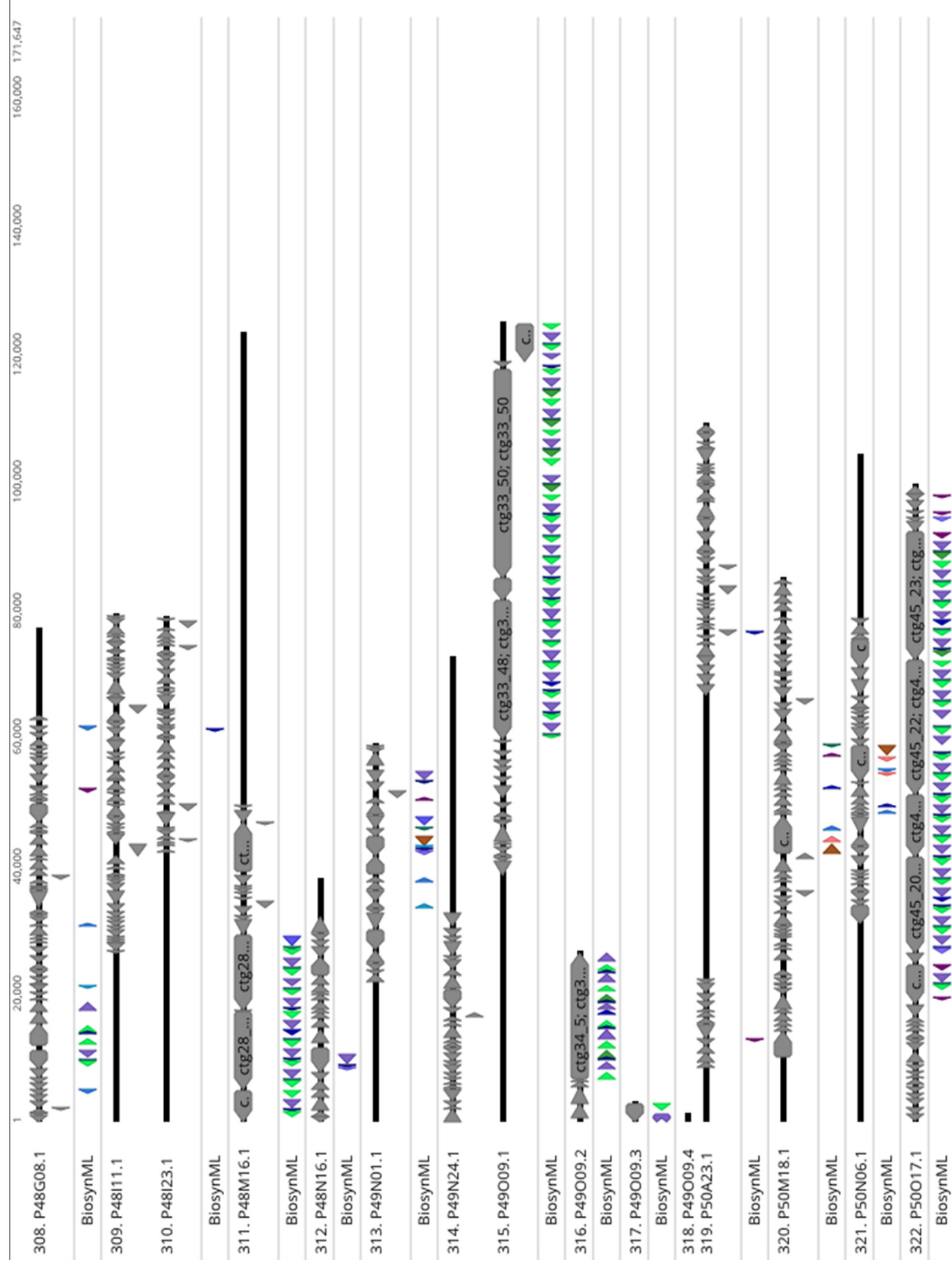

Supplement: Supplementary Figure 6 — AntiSMASH 4.0 annotations for each contig and clones predicted to carry a PKS and/or NRPS pathway. (A) Contigs predicted to carry NRPS pathways; (B) Contigs predicted to carry PKS pathways; (C) Resequenced clones. [file Data_Sheet_1.PDF]
