# Supplementary material for: Discovery of Novel Biosynthetic Gene Cluster Diversity From a Soil Metagenomic Library
Source: Front Microbiol. 2020 Dec 7;11:585398. doi: 10.3389/fmicb.2020.585398 (PMC7750434; doi:10.3389/fmicb.2020.585398)
Supplement: Supplementary Table 1 — List of oligonucleotides sequences used in this study. [file Table_1.DOCX]

| Biosynthetic Gene Cluster Type | PCR Screening | NGS Screening | NGS Screening Deduplicated | Deconvoluted (%) |
| --- | --- | --- | --- | --- |
| Type I PKS | 17 | 75 | 39 | 73.3 |
| Type I PKS-NRPS | 27 | 100 | 33 | 86.0 |
| Type II PKS | 0 | 22 | 12 | 90.9 |
| Type III PKS | 1 | 114 | 71 | 84.2 |
| Transatpks | 1 | 6 | 3 | 100.0 |
| Other KS | 4 | 14 | 9 | 78.6 |
| NRPS | 0 | 509 | 160 | 68.4 |
| Other | 0 | 213 | 110 | 70.0 |
| Terpene | 0 | 371 | 201 | 80.9 |
| Bacteriocin | 0 | 224 | 127 | 77.2 |
| Arylpolyene | 0 | 70 | 36 | 67.1 |
| Lassopeptide | 0 | 61 | 32 | 83.6 |
| Lantipeptide | 0 | 33 | 23 | 84.8 |
| Hserlactone | 0 | 27 | 16 | 74.1 |
| Resorcinol | 0 | 17 | 14 | 88.2 |
| Phosphonate | 0 | 15 | 11 | 93.3 |
| Indole | 0 | 13 | 5 | 46.2 |
| Ladderane | 0 | 17 | 16 | 82.4 |
| Acyl Aminoacids | 0 | 10 | 8 | 90.0 |
| Butyrolactone | 0 | 4 | 3 | 75.0 |
| Microviridin | 0 | 8 | 4 | 100.0 |
| Siderophore | 0 | 4 | 4 | 100.0 |
| Cyanobactin | 0 | 2 | 1 | 100.0 |
| Thiopeptide | 0 | 3 | 2 | 100.0 |
| Linaridin | 0 | 1 | 1 | 100.0 |
| Phenazine | 0 | 1 | 1 | 100.0 |
| Ectoine | 0 | 1 | 1 | 100.0 |
| Hybrid Pathways* | 0 | 147 | 72 | 100.0 |
| Total | 50 | 2082 | 1015 | 77.5 |
