# Supplementary material for: Discovery of Novel Biosynthetic Gene Cluster Diversity From a Soil Metagenomic Library
Source: Front Microbiol. 2020 Dec 7;11:585398. doi: 10.3389/fmicb.2020.585398 (PMC7750434; doi:10.3389/fmicb.2020.585398)
Supplement: Supplementary Table 2 — Basic statistics of contig lengths according to deconvolution status. [file Table_2.DOCX]

| Oligonucleotide name | Oligonucleotide sequence |
| --- | --- |
| PKSc1OligoA | CGAGCATGCCGTGTCGATCGCCA |
| PKSc1OligoB | TCAAAGGTCCGTGCATGGCGATC |
| PKSc2OligoA | GGGCCTCTATATATGTCACGTCT |
| PKSc2OligoB | CGAAGTCCATGCTCAAGACGTGA |
| PKSc3OligoA | CTCCAGTTGTGCATTCAGCAGCG |
| PKSc3OligoB | GGTTAATTCGGGAAGCGCTGCTG |
| PKSc4OligoA | TCGCGCAACGCGTCGGAAAGCCT |
| PKSc4OligoB | ATCCTCGTGCTCAAGAGGCTTTC |
| PKSc9OligoA | CGTATAGCCGACTTTCGCCGACC |
| PKSc9OligoB | CCATCAACAACGACGGGTCGGCG |
| PKSc11OligoA | ACATAGCCGATTTCATTGGGGTT |
| PKSc11OligoB | GCTCGCAACGGCATCAACCCCAA |
| PKSc20OligoA | TGGCCATCCGGAGAAAGGATCAT |
| PKSc20OligoB | CTTTTCCAGGAAGGGATGATCCT |
| 5LL | GGRTCNCCIARYTGIGTICCIGTICCRTGIGC |
| 4UU | MGIGARGCIYTICARATGGAYCCICARCARMG |
