# Supplementary material for: Discovery of Novel Biosynthetic Gene Cluster Diversity From a Soil Metagenomic Library
Source: Front Microbiol. 2020 Dec 7;11:585398. doi: 10.3389/fmicb.2020.585398 (PMC7750434; doi:10.3389/fmicb.2020.585398)
Supplement: Supplementary Table 3 — PKS and/or NRPS contigs and resequenced clones information. “BL mean” refers to the mean Branch Length of all KS and/or A domains from the BGC; “Domain Ident” is the mean percent identity for the top BLASTp hit for all A domain and/or KS domains identified from re-sequenced metagenomic clones compared to the NCBI nr/nt database.“Dom. Div” refers to domain divergence calculated as the opposite of Domain %ID (1-Domain %ID). [file Table_3.DOCX]

| **DECONVSTAT** | **Mean** | **Standard Deviation** | **Median** |
| --- | --- | --- | --- |
| Complete | 1860.8287 | 7879.012 | 393 |
| Partial | 936.8280 | 4614.699 | 319 |
| Singleton | 540.4453 | 2234.693 | 299 |
