# Supplementary material for: Discovery of Novel Biosynthetic Gene Cluster Diversity From a Soil Metagenomic Library
Source: Front Microbiol. 2020 Dec 7;11:585398. doi: 10.3389/fmicb.2020.585398 (PMC7750434; doi:10.3389/fmicb.2020.585398)
Supplement: Supplementary file 10 [file Table_4.DOCX]

| Clone | Contig | BLmean | Identified | Domains | Insert Length | BGC Length | GC (%) | Set | Type | Truncated | Domain Ident | Dom Div | DivT |
| --- | --- | --- | --- | --- | --- | --- | --- | --- | --- | --- | --- | --- | --- |
| P01H15 | P01A1.21 | 2.59749 | NGS | 15 | 64339 | 4,722 | 71.3 | Set1 | PKS-NRPS | Complete | 27.70 | 72.30 | 94.84 |
| P01N03 | P01B1.33 P01B1.73 | 2.5038 | NGS | 21 | 40763 | 41,349 | 58.1 | Set1 | NRPS | Complete | 24.41 | 75.59 | 99.15 |
| P02O05 | P02A1.12 | 2.715957 | NGS | 9 | 106305 | 13,762 | 56.3 | Set1 | NRPS | Complete | 27.94 | 72.06 | 94.53 |
| P04H02 | P04B1.15 P04B1.67 | 1.374 | NGS | 72 | 140742 | 97,614 | 54.8 | Set1 | PKS-NRPS | Complete | 60 | 40.00 | 52.47 |
| P05D13 | P05B1.29 | 2.631503 | NGS | 35 | 71074 | 51,314 | 54.7 | Set1 | NRPS | Complete | 26.99 | 73.01 | 95.77 |
| P05G19 | P05A1.676 | 3.38761 | NGS | 20 | 90869 | 39,049 | 54.2 | Set1 | NRPS | Complete | 24.84 | 75.16 | 98.59 |
| P06G05 | P06A1.1 | 2.8049 | NGS | 18 | 102475 | 68,150 | 56 | Set1 | PKS-NRPS | Complete | 52.69 | 47.31 | 62.06 |
| P06G11 | P06A1.285 | 1.9081 | NGS | 52 | 129397 | 52,348 | 55.8 | Set1 | PKS-NRPS | Complete | 28.95 | 71.05 | 93.20 |
| P07D12 | P07B2.21 | 2.805976 | NGS | 20 | 115226 | 24,317 | 57.2 | Set1 | NRPS | Complete | 31.72 | 68.28 | 89.57 |
| P07H07 | P07B1.72 | 2.750188 | NGS | 20 | 30585 | 37,239 | 54.2 | Set1 | NRPS | Complete | 28.01 | 71.99 | 94.44 |
| P07H14 | P07B2.159 P07B2.164 | 2.688972 | NGS | 20 | 130799 | 114,768 | 61.9 | Set1 | NRPS | Complete | 34.45 | 65.55 | 85.99 |
| P07O09 | P07A1.56 | 1.67399 | NGS | 16 | 108907 | 87,047 | 55.6 | Set1 | PKS-NRPS | Complete | 58.64 | 41.36 | 54.25 |
| P08A19 | P08A1.2 | 1.7239 | NGS | 38 | 96916 | 43,697 | 56 | Set1 | PKS-NRPS | Complete | 55.84 | 44.16 | 57.93 |
| P08C17 | P08A2.46 P08A1.540 P08A1.626 P08A1.683 | 3.096427 | NGS | 33 | 124248 | 6,693 | 55.2 | Set1 | NRPS | Complete | 67.09 | 32.91 | 43.18 |
| P08J17 | P08A2.29 | 2.714854 | NGS | 34 | 106688 | 42,699 | 54.1 | Set1 | NRPS | Complete | 57.13 | 42.87 | 56.24 |
| P08M24 | P08A2.142 | 2.85275 | NGS | 57 | 133951 | 43,032 | 58.2 | Set1 | PKS-NRPS | Complete | 61.46 | 38.54 | 50.56 |
| P09L06 | P09B2.14 | 1.899525 | NGS | 27 | 75828 | 26,416 | 54.8 | Set1 | PKS-NRPS | Complete | 61.22 | 38.78 | 50.87 |
| P09M22 | P09A2.103 | 2.558026 | NGS | 46 | 99134 | 1,624 | 54.7 | Set1 | PKS-NRPS | Complete | 58.84 | 41.16 | 53.99 |
| P09P02 | P09B2.417 P09B2.194 P09B2.157 | 2.667553 | NGS | 19 | 143164 | 1,040 | 70.4 | Set1 | NRPS | Complete | 58.17 | 41.83 | 54.87 |
| P11H15 | P11B1.15 | 4.09459 | NGS | 25 | 77085 | 66,479 | 54.8 | Set2 | NRPS | Complete | 54.90 | 45.10 | 59.16 |
| P11I24 | P11A2.58 | 1.700657 | NGS | 45 | 68208 | 57,478 | 54 | Set2 | PKS-NRPS | Complete | 64.22 | 35.78 | 46.94 |
| P11J18 | P11B2.361 P11B2.716 P11B2.149 P11B2.133 P11B2.172 P11B2.474 | 2.727884 | NGS | 11 | 42994 | 87,614 | 50.6 | Set2 | NRPS | Complete | 55.59 | 44.41 | 58.26 |
| P11N21 | P11B1.351 P11B1.159 | 3.406645 | NGS | 11 | 49818 | 31,688 | 50.8 | Set2 | NRPS | Complete | 65.23 | 34.77 | 45.61 |
| P15C22 | P15A1.77 P15A2.23 | 3.071734 | NGS | 18 | 83712 | 39,703 | 62.8 | Set2 | NRPS | Complete | 63.31 | 36.69 | 48.13 |
| P17G08 | P17A2.40 | 2.65185 | NGS | 11 | 65444 | 2,884 | 56.5 | Set2 | NRPS | Complete | 52.67 | 47.33 | 62.08 |
| P19N16 | P19B2.404 | 2.59971 | NGS | 20 | 60112 | 2,302 | 58.3 | Set2 | NRPS | Complete | 58.39 | 41.61 | 54.59 |
| P20P17 | P20B1.264 P20B1.518 | 2.589025 | NGS | 11 | 26094 | 49,110 | 63.6 | Set2 | NRPS | Complete | 54.24 | 45.76 | 60.03 |
| P22K24 | P22A2.182 | 1.8373 | NGS | 34 | 105147 | 149,469 | 54 | Set3 | NRPS | Complete | 56.29 | 43.71 | 57.34 |
| P22P12 | P22B2.239 | 2.652798 | NGS | 13 | 44041 | 3,014 | 53.2 | Set3 | NRPS | Complete | 59.41 | 40.59 | 53.24 |
| P23K14 | P20B1.242 | 2.51521 | NGS | 30 | 43775 | 48,479 | 71.7 | Set3 | PKS-NRPS | Complete | 59.63 | 40.37 | 52.95 |
| P24C17 | P24A1.31 | 2.667338 | NGS | 26 | 79331 | 77,061 | 53.6 | Set3 | NRPS | Complete | 51.55 | 48.45 | 63.55 |
| P25M03 | P25A1.10 | 2.607858 | NGS | 29 | 87165 | 71,332 | 54.8 | Set3 | NRPS | Complete | 56.34 | 43.66 | 57.28 |
| P26P07 | P26B1.150 P26B1.124 P26B1.102 P26B1.83 P26B1.192 | 2.66871 | NGS | 26 | 37748 | 25,461 | 58.4 | Set3 | NRPS | Complete | 55.06 | 44.94 | 58.96 |
| P27K06 | P27A2.75 | 1.9834 | NGS | 41 | 139647 | 20,072 | 57 | Set3 | NRPS | Complete | 58.92 | 41.08 | 53.89 |
| P27O06 | P27A2.39 | 2.733196 | NGS | 41 | 139647 | 2,970 | 57.4 | Set3 | NRPS | Complete | 56.45 | 43.55 | 57.13 |
| P27O09 | P27A1.38 | 1.60135 | NGS | 49 | 132241 | 20,272 | 54.8 | Set3 | PKS-NRPS | Complete | 56.00 | 44.00 | 57.72 |
| P27P01 | P27B1.24 | 2.588137 | NGS | 15 | 113937 | 10,545 | 52.9 | Set3 | NRPS | Complete | 57.04 | 42.97 | 56.36 |
| P28L15 | P28B1.439 P28B1.133 P28B1.257 P28B1.63 P28B1.237 P28B1.390 | 2.837996 | NGS | 20 | 110926 | 23,644 | 55.7 | Set3 | NRPS | Complete | 53.37 | 46.63 | 61.17 |
| P28O04 | P28A2.202 | 1.64563 | NGS | 30 | 85938 | 77,087 | 61 | Set3 | NRPS | Complete | 56.14 | 43.86 | 57.53 |
| P29F04 | P29B2.3 | 2.672458 | NGS | 30 | 133026 | 48,625 | 53.1 | Set3 | NRPS | Complete | 58.91 | 41.09 | 53.90 |
| P29O18 | P29A2.15 | 1.80395 | NGS | 77 | 114397 | 117,998 | 56.6 | Set3 | PKS-NRPS | Complete | 54.79 | 45.21 | 59.30 |
| P35G10 | P35A2.492 P35A2.22 | 2.596979 | NGS | 42 | 69371 | 5,532 | 56.7 | Set4 | NRPS | Complete | 55.83 | 44.17 | 57.94 |
| P39F17 | P39B1.116 | 2.641278 | NGS | 13 | 25361 | 50,960 | 58.6 | Set4 | NRPS | Complete | 55.45 | 44.55 | 58.44 |
| P39I02 | P39A1.8 | 1.79775 | NGS | 15 | 135991 | 70,579 | 57.6 | Set4 | PKS-NRPS | Complete | 57.94 | 42.06 | 55.17 |
| P39I17 | P39A1.32 P39B1.24 | 2.844745 | NGS | 42 | 92944 | 73,906 | 64.7 | Set4 | NRPS | Complete | 53.72 | 46.28 | 60.72 |
| P42D19 | P42.1469 P42.1108 P42.2018 P42.1840 | 2.888025 | NGS | 20 | 95303 | 112,607 | 55 | Set5 | NRPS | Complete | 51.27 | 48.73 | 63.93 |
| P42K11 | P42.2025 | 3.30237 | NGS | 30 | 116916 | 30,675 | 56.5 | Set5 | NRPS | Complete | 54.19 | 45.81 | 60.09 |
| P42P24 | P42.2475 | 3.95117 | NGS | 13 | 19065 | 65,394 | 53.9 | Set5 | NRPS | Complete | 51.40 | 48.60 | 63.75 |
| P43D10 | P43.1515 | 2.558 | NGS | 36 | 129280 | 99,228 | 53.9 | Set5 | NRPS | Complete | 57.25 | 42.75 | 56.08 |
| P44L04 | P44.129 | 1.1188 | NGS | 23 | 53866 | 101,858 | 56 | Set5 | PKS-NRPS | Complete | 56.14 | 43.86 | 57.54 |
| P45H03 | P45.341 | 2.668237 | NGS | 19 | 57229 | 72,087 | 59.2 | Set5 | NRPS | Complete | 52.39 | 47.61 | 62.45 |
| P48M16 | P48.926 P48.1895 P48.1710 P48.988 P48.831 | 2.738415 | NGS | 26 | 123238 | 14,386 | 53.9 | Set5 | NRPS | Complete | 56.64 | 43.36 | 56.88 |
| P49O09 | P49.2215 P49.1560 P49.2119 | 2.70316 | NGS | 57 | 124799 | 2,251 | 71.9 | Set5 | NRPS | Complete | 53.84 | 46.16 | 60.56 |
| P50O17 | P50.1556 P50.1984 | 2.75925 | NGS | 67 | 99486 | 105,370 | 42.3 | Set5 | NRPS | Complete | 54.06 | 45.94 | 60.27 |
| P11I06 | P11A2.14 | 2.606004 | NGS | 29 | 106068 | 69,570 | 52.8 | Set2 | NRPS | Complete | 71 | 29.00 | 38.04 |
| P15P18 | P15B2.5 | 2.600735 | NGS | 25 | 81084 | 70,052 | 54.6 | Set2 | NRPS | Complete | 58 | 42.00 | 55.10 |
| P16O06 | P17B2.38 | 2.754474 | NGS | 17 | 67482 | 42,261 | 54.6 | Set2 | NRPS | Complete | 56 | 44.00 | 57.72 |
| P20H02 | P20A2.2 | 1.66405 | NGS | 9 | 134998 | 30,345 | 57 | Set2 | PKS-NRPS | Complete | 83 | 17.00 | 22.30 |
| P21E03 | P21A1.38 | 2.818178 | NGS | 15 | 91188 | 53,798 | 54.8 | Set3 | NRPS | Complete | 57 | 43.00 | 56.41 |
| P28O18 | P28A2.23 | 0.950076 | NGS | 30 | 85938 |  | 61 | Set3 | NRPS | Complete | 55 | 45.00 | 59.03 |
| P32P04 | P32B2.12 | 1.2293 | NGS | 12 | 45372 | 44,670 | 61 | Set4 | PKS-NRPS | Complete | 70 | 30.00 | 39.35 |
| P33I07 | P33A1.40 | 2.031962 | NGS | 30 | 91908 | 53,131 | 40 | Set4 | PKS | Complete | 44 | 56.00 | 73.46 |
| P35K06 | P35A2.331 | 1.81645 | NGS | 13 | 142359 | 50,486 | 66 | Set4 | PKS-NRPS | Complete | 62 | 38.00 | 49.85 |
| P36D09 | P36B1.54 P36B1.60 | 2.83628 | NGS | 30 | 76453 | 75,565 | 58.8 | Set4 | NRPS | Complete | 55 | 45.00 | 59.03 |
| P01A07 | P01A1.48 | 1.4943 | NGS-PCR | 33 | 128761 | 96,149 | 69 | Set1 | PKS-NRPS | Complete | 38.51 | 61.49 | 80.67 |
| P07H03 | P07B1.70 | 1.103 | NGS-PCR | 16 | 31569 | 79,592 | 70.7 | Set1 | PKS-NRPS | Complete | 43.77 | 56.23 | 73.76 |
| P09D05 | P09B1.149 | 1.1741 | NGS-PCR | 39 | 63459 | 1,116 | 59 | Set1 | PKS-NRPS | Complete | 62.38 | 37.62 | 49.36 |
| P09I20 | P09B2.14 | 1.899525 | NGS-PCR | 21 | 30553 | 73,864 | 59.6 | Set1 | NRPS | Complete | 56.71 | 43.29 | 56.79 |
| P10B06 | P10B2.42 | 1.87925 | NGS-PCR | 16 | 70533 | 49,433 | 62 | Set1 | PKS | Complete | 69.23 | 30.77 | 40.37 |
| P11O04 | P11A2.44 | 1.32454 | NGS-PCR | 31 | 77067 | 74,462 | 67.7 | Set2 | PKS-NRPS | Complete | 58.19 | 41.81 | 54.85 |
| P11P20 | P11B2.532 | 1.808 | NGS-PCR | 59 | 150553 | 31,118 | 65 | Set2 | PKS-NRPS | Complete | 65.98 | 34.02 | 44.62 |
| P17F15 | P17B1.340 | 1.1501 | NGS-PCR | 10 | 71189 | 88,261 | 70 | Set2 | PKS-NRPS | Complete | 64.33 | 35.67 | 46.79 |
| P37L16 | P37L16 | 1.614667 | NGS-PCR | 17 | 57334 | 4,799 | 63.8 | Set4 | PKS-NRPS | Complete | 54.39 | 45.61 | 59.83 |
| P38G02 | P38G02 | 1.9029 | NGS-PCR | 24 | 91476 | 3,192 | 54.6 | Set4 | PKS-NRPS | Complete | 56.40 | 43.60 | 57.19 |
| P45H08 | P45.362 | 1.6673 | NGS-PCR | 38 | 146800 | 70,241 | 53 | Set5 | PKS-NRPS | Complete | 59.02 | 40.98 | 53.76 |
| P02K21 | P02A1.196 | 2.55024 | NGS | 2 | 107433 | 14,523 | 65.5 | Set1 | NRPS | Truncated | 23.78 | 76.22 | 99.99 |
| P03C03 | P03A1.52 | 2.82462 | NGS | 6 | 62165 | 118,628 | 54.6 | Set1 | NRPS | Truncated | 23.77 | 76.23 | 100.00 |
| P03G12 | P03A2.665 | 2.37516 | NGS | 5 | 90962 | 104,170 | 65.1 | Set1 | NRPS | Truncated | 28.06 | 71.94 | 94.37 |
| P03G13 | P03A1.91 | 2.90367 | NGS | 1 | 123646 | 1,510 | 51.6 | Set1 | NRPS | Truncated | 64.59 | 35.41 | 46.45 |
| P04F05 | P04B1.68 | 2.896505 | NGS | 8 | 129359 | 98,035 | 71.1 | Set1 | NRPS | Truncated | 28.59 | 71.41 | 93.68 |
| P04O01 | P04A1.29 | 1.1822 | NGS | 8 | 53001 | 2,609 | 57.7 | Set1 | PKS | Truncated | 51.06 | 48.94 | 64.20 |
| P05D19 | P05A1.115 P05A1.255 | 2.652045 | NGS | 5 | 103884 | 153,088 | 52.1 | Set1 | NRPS | Truncated | 26.35 | 73.65 | 96.62 |
| P05K23 | P05A1.752 | 3.65239 | NGS | 4 | 108335 | 1,278 | 54.6 | Set1 | NRPS | Truncated | 24.32 | 75.68 | 99.27 |
| P05P18 | P05B2.4 | 2.68926 | NGS | 5 | 121566 | 58,216 | 57 | Set1 | NRPS | Truncated | 47.34 | 52.66 | 69.08 |
| P06A05 | P06A1.18 | 1.85877 | NGS | 9 | 87898 | 83,133 | 57 | Set1 | PKS-NRPS | Truncated | 38.07 | 61.93 | 81.24 |
| P07E15 | P07A1.117 | 2.88421 | NGS | 10 | 133243 | 66,517 | 54.1 | Set1 | NRPS | Truncated | 26.01 | 73.99 | 97.07 |
| P07L16 | P07B2.36 | 2.562933 | NGS | 8 | 70028 | 93,770 | 65 | Set1 | PKS | Truncated | 67.76 | 32.24 | 42.29 |
| P07O04 | P07A2.48 | 0.972874 | NGS | 10 | 61286 | 106,177 | 66 | Set1 | PKS-NRPS | Truncated | 81.67 | 18.33 | 24.05 |
| P07P02 | P07B2.19 | 2.437705 | NGS | 6 | 83740 | 145,642 | 66.1 | Set1 | NRPS | Truncated | 60.60 | 39.40 | 51.69 |
| P09G10 | P09A2.159 | 2.64345 | NGS | 11 | 112164 | 15,283 | 67.7 | Set1 | PKS-NRPS | Truncated | 50.24 | 49.76 | 65.27 |
| P09J07 | P09A1.116 P09B1.70 | 2.86057 | NGS | 5 | 46842 | 80,512 | 70.5 | Set1 | NRPS | Truncated | 60.80 | 39.20 | 51.42 |
| P11I13 | P11A1.18 | 2.02242 | NGS | 11 | 110052 | 78,569 | 63 | Set2 | PKS | Truncated | 47.14 | 52.86 | 69.34 |
| P19O08 | P19A2.56 | 1.228433 | NGS | 11 | 93369 | 58,388 | 41.9 | Set2 | PKS | Truncated | 63.87 | 36.13 | 47.39 |
| P20F15 | P20B1.242 P20B1.293 | 2.721065 | NGS | 30 | 75278 | 53,104 | 68.5 | Set2 | PKS-NRPS | Truncated | 59.63 | 40.37 | 52.95 |
| P24H02 | P24B2.16 | 1.98759 | NGS | 6 | 87129 | 67,260 | 47.8 | Set3 | PKS | Truncated | 62.06 | 37.94 | 49.77 |
| P24J10 | P24B2.83 | 2.11339 | NGS | 3 | 40322 | 24,265 | 67.7 | Set3 | NRPS | Truncated | 58.61 | 41.39 | 54.29 |
| P24L04 | P24B2.4 | 3.008657 | NGS | 10 | 142547 | 1,664 | 41.7 | Set3 | NRPS | Truncated | 55.41 | 44.59 | 58.49 |
| P24L06 | P24B2.26 | 1.79834 | NGS | 8 | 97272 | 97,771 | 40 | Set3 | PKS | Truncated | 59.07 | 40.93 | 53.69 |
| P25I09 | P25A1.149 | 2.5978 | NGS | 8 | 70076 | 4,483 | 55.5 | Set3 | NRPS | Truncated | 55.03 | 44.97 | 58.99 |
| P27L24 | P27B2.506 | 2.71706 | NGS | 12 | 112758 | 5,913 | 55.1 | Set3 | NRPS | Truncated | 54.73 | 45.27 | 59.38 |
| P28I16 | P28A2.16 | 2.1338 | NGS | 10 | 74990 | 38,208 | 55 | Set3 | PKS-NRPS | Truncated | 64.65 | 35.35 | 46.37 |
| P29G19 | P29A1.75 | 2.5445 | NGS | 5 | 63402 | 2,653 | 63.9 | Set3 | NRPS | Truncated | 53.79 | 46.21 | 60.62 |
| P32A16 | P32A2.546 | 2.66837 | NGS | 9 | 59698 | 83,726 | 68.7 | Set4 | NRPS | Truncated | 55.84 | 44.16 | 57.93 |
| P33A17 | P33A1.19 | 2.755103 | NGS | 8 | 114632 | 34,816 | 54.2 | Set4 | NRPS | Truncated | 54.59 | 45.41 | 59.57 |
| P33E15 | P33A1.78 P33A1.142 | 2.141635 | NGS | 5 | 125482 | 36,538 | 63.9 | Set4 | PKS | Truncated | 83.99 | 16.01 | 21.01 |
| P38F23 | P38B1.47 | 2.8209 | NGS | 9 | 35834 | 3,270 | 64.8 | Set4 | NRPS | Truncated | 55.15 | 44.85 | 58.84 |
| P38H14 | P38B2.118 | 2.41933 | NGS | 6 | 106831 | 32,332 | 53.8 | Set4 | NRPS | Truncated | 52.42 | 47.58 | 62.41 |
| P38N20 | P38B2.22 | 2.57864 | NGS | 5 | 96293 | 5,942 | 54 | Set4 | NRPS | Truncated | 47.90 | 52.10 | 68.34 |
| P39E24 | P39A2.5 P39A1.8 | 2.648825 | NGS | 5 | 57207 | 5,643 | 62 | Set4 | PKS-NRPS | Truncated | 57.73 | 42.27 | 55.45 |
| P39I13 | P39A1.70 | 3.20332 | NGS | 5 | 51616 | 26,123 | 58.1 | Set4 | NRPS | Truncated | 50.75 | 49.25 | 64.61 |
| P40C07 | P40A1.53 | 2.680755 | NGS | 9 | 142632 | 103,818 | 58.3 | Set4 | NRPS | Truncated | 57.64 | 42.36 | 55.56 |
| P40C19 | P40A1.32 | 1.7382 | NGS | 5 | 113503 | 9,815 | 65.9 | Set4 | NRPS | Truncated | 54.23 | 45.77 | 60.04 |
| P40O18 | P40A2.5 | 2.004605 | NGS | 12 | 136500 | 1,137 | 69 | Set4 | NRPS | Truncated | 76.74 | 23.26 | 30.51 |
| P41O18 | P41.7 | 1.92096 | NGS | 9 | 61544 | 52,280 | 62.7 | Set5 | PKS | Truncated | 62.82 | 37.18 | 48.77 |
| P44H15 | P44.1391 | 2.70924 | NGS | 15 | 100008 | 41,973 | 53.5 | Set5 | PKS-NRPS | Truncated | 58.85 | 41.15 | 53.98 |
| P47I11 | P47.1080 | 2.55545 | NGS | 4 | 26351 | 76,463 | 58.4 | Set5 | NRPS | Truncated | 37.71 | 62.30 | 81.72 |
| P48G08 | P48.1239 P48.682 | 3.42013 | NGS | 12 | 76978 | 46,342 | 71 | Set5 | NRPS | Truncated | 52.15 | 47.86 | 62.78 |
| P48N16 | P48.126 | 2.67936 | NGS | 3 | 37665 | 12,723 | 57.7 | Set5 | NRPS | Truncated | 49.78 | 50.22 | 65.88 |
| P49N01 | P49.44 | 0.885196 | NGS | 11 | 58826 | 10,790 | 62 | Set5 | PKS-NRPS | Truncated | 57.67 | 42.33 | 55.53 |
| P50M18 | P50.95 | 2.18771 | NGS | 6 | 84718 | 1,755 | 57.1 | Set5 | PKS | Truncated | 90.23 | 9.77 | 12.82 |
| P50N06 | P50.4 | 2.0896 | NGS | 6 | 104104 | 59,363 | 37.8 | Set5 | PKS | Truncated | 78.34 | 21.66 | 28.42 |
| P03B10 | P03B2.241 | 2.67331 | NGS | 4 | 80352 | 24,854 | 52.8 | Set1 | NRPS | Truncated | 55 | 45.00 | 59.03 |
| P15P01 | P15B1.63 | 2.68009 | NGS | 8 | 132498 | 45,875 | 56.1 | Set2 | NRPS | Truncated | 67 | 33.00 | 43.29 |
| P17B06 | P17B2.114 | 2.07415 | NGS | 12 | 100101 | 105,964 | 60 | Set2 | NRPS | Truncated | 47 | 53.00 | 69.53 |
| P17I02 | P17A2.30 | 2.586536 | NGS | 10 | 125265 | 43,874 | 66.2 | Set2 | NRPS | Truncated | 67 | 33.00 | 43.29 |
| P18H23 | P18A1.398 | 2.67571 | NGS | 3 | 51470 | 25,813 | 61.6 | Set2 | NRPS | Truncated | 45 | 55.00 | 72.15 |
| P19H07 | P19B1.221 | 2.83341 | NGS | 2 | 16484 | 16,484 | 52.7 | Set2 | NRPS | Truncated | 53 | 47.00 | 61.66 |
| P19L22 | P19A2.82 P19B2.34 | 3.16285 | NGS | 7 | 87188 | 51,653 | 56.3 | Set2 | NRPS | Truncated | 35 | 65.00 | 85.27 |
| P21A05 | P21A1.60 | 2.960735 | NGS | 4 | 54244 | 26,263 | 31.3 | Set3 | NRPS | Truncated | 57 | 43.00 | 56.41 |
| P23I15 | P21A1.65 | 2.9474 | NGS | 4 | 61549 | 42,000 | 55 | Set3 | PKS | Truncated | 70 | 30.00 | 39.35 |
| P25F10 | P25B2.32 | 3.17367 | NGS | 5 | 64797 | 45,350 | 62.7 | Set3 | NRPS | Truncated | 37 | 63.00 | 82.64 |
| P25O21 | P25A1.147 | 2.758465 | NGS | 8 | 37775 | 29,896 | 62.4 | Set3 | NRPS | Truncated | 63 | 37.00 | 48.54 |
| P26D23 | P26B1.113 | 2.728533 | NGS | 3 | 51817 | 24,124 | 57.3 | Set3 | NRPS | Truncated | 59 | 41.00 | 53.78 |
| P27C17 | P27A1.12 | 3.39678 | NGS | 7 | 102918 | 47,322 | 41.2 | Set3 | NRPS | Truncated | 33 | 67.00 | 87.89 |
| P28C19 | P28A1.563 | 2.72299 | NGS | 3 | 127612 | 43,223 | 64 | Set3 | NRPS | Truncated | 47 | 53.00 | 69.53 |
| P28H21 | P28B1.114 | 2.84304 | NGS | 3 | 28688 | 25,219 | 55.4 | Set3 | NRPS | Truncated | 56 | 44.00 | 57.72 |
| P29M03 | P29A1.109 | 3.7476 | NGS | 5 | 97291 | 47,644 | 63.9 | Set3 | PKS | Truncated | 49 | 51.00 | 66.90 |
| P33F15 | P33B1.42 | 1.78757 | NGS | 5 | 125482 | 35,404 | 63.9 | Set4 | NRPS | Truncated | 57 | 43.00 | 56.41 |
| P34D08 | P34B2.15 | 2.780065 | NGS | 7 | 29301 | 27,435 | 53.8 | Set4 | NRPS | Truncated | 61 | 39.00 | 51.16 |
| P34F16 | P34B2.447 | 3.01348 | NGS | 4 | 58820 | 25,347 | 56.1 | Set4 | NRPS | Truncated | 58 | 42.00 | 55.10 |
| P35M06 | P35A2.5 | 1.79834 | NGS | 5 | 96287 | 30,405 | 40 | Set4 | PKS | Truncated | 84 | 16.00 | 20.99 |
| P38B06 | P38B2.77 | 2.54513 | NGS | 7 | 91764 | 47,012 | 70.1 | Set4 | NRPS | Truncated | 56 | 44.00 | 57.72 |
| P38J21 | P38B1.620 | 3.05963 | NGS | 8 | 97705 | 34,263 | 62.5 | Set4 | NRPS | Truncated | 95 | 5.00 | 6.56 |
| P48E04 | P49.261 | 2.61969 | NGS | 6 | 88481 | 49,571 | 63.6 | Set5 | NRPS | Truncated | 44 | 56.00 | 73.46 |
| P04E17 | P04A1.356 | 1.74144 | NGS-PCR | 12 | 117009 | 37,401 | 47.9 | Set1 | PKS-NRPS | Truncated | 43.94 | 56.06 | 73.54 |
| P07M04 | P07A1.173 | 0.977724 | NGS-PCR | 5 | 11583 | 39,662 | 64.8 | Set1 | PKS-NRPS | Truncated | 71.17 | 28.83 | 37.82 |
| P09P03 | P09B1.20 | 1.66243 | NGS-PCR | 11 | 110970 | 73,677 | 63.8 | Set1 | PKS-NRPS | Truncated | 56.22 | 43.78 | 57.43 |
| P22N20 | P22B2.30 | 1.2852 | NGS-PCR | 8 | 103714 | 98,719 | 52 | Set3 | PKS | Truncated | 78.12 | 21.88 | 28.71 |
| P24H13 | P24B1.29 | 1.83181 | NGS-PCR | 16 | 99579 | 43,697 | 51.6 | Set3 | PKS-NRPS | Truncated | 81.82 | 18.18 | 23.85 |
| P35L08 | P35B2.21 | 1 | NGS-PCR | 10 | 81817 | 4,886 | 59 | Set4 | PKS | Truncated | 62.95 | 37.05 | 48.60 |
| P34D04 | P34B2.375 | 2.45349 | NGS-PCR | 3 | 68400 | 31,669 | 59.9 | Set4 | NRPS | Truncated | 43 | 57.00 | 74.77 |
